# Bioinspired Framework Catalysts: From Enzyme Immobilization to Biomimetic Catalysis

**DOI:** 10.1021/acs.chemrev.2c00879

**Published:** 2023-04-12

**Authors:** Kun-Yu Wang, Jiaqi Zhang, Yu-Chuan Hsu, Hengyu Lin, Zongsu Han, Jiandong Pang, Zhentao Yang, Rong-Ran Liang, Wei Shi, Hong-Cai Zhou

**Affiliations:** † Department of Chemistry, Texas A&M University, College Station, Texas 77843, United States; ⊥ Department of Chemistry, Key Laboratory of Advanced Energy Materials Chemistry (MOE) and Renewable Energy Conversion and Storage Center (RECAST), College of Chemistry, Nankai University, Tianjin 300071, China; § School of Materials Science and Engineering, Tianjin Key Laboratory of Metal and Molecule-Based Material Chemistry, Nankai University, Tianjin 300350, China

## Abstract

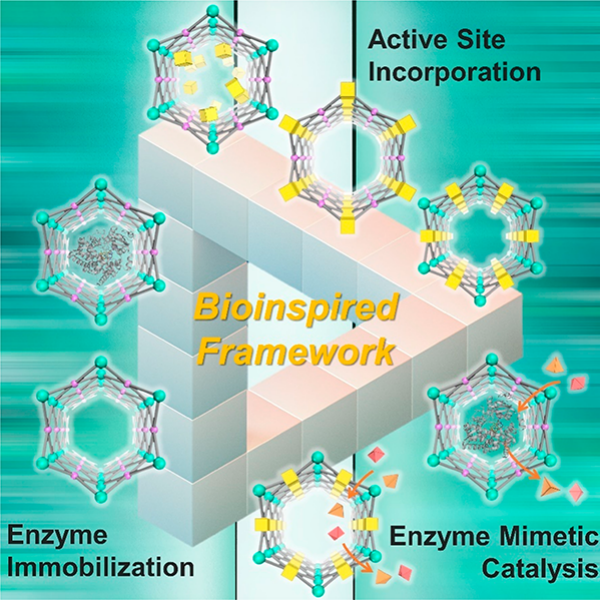

Enzymatic catalysis has fueled considerable interest from chemists due to its high efficiency and selectivity. However, the structural complexity and vulnerability hamper the application potentials of enzymes. Driven by the practical demand for chemical conversion, there is a long-sought quest for bioinspired catalysts reproducing and even surpassing the functions of natural enzymes. As nanoporous materials with high surface areas and crystallinity, metal–organic frameworks (MOFs) represent an exquisite case of how natural enzymes and their active sites are integrated into porous solids, affording bioinspired heterogeneous catalysts with superior stability and customizable structures. In this review, we comprehensively summarize the advances of bioinspired MOFs for catalysis, discuss the design principle of various MOF-based catalysts, such as MOF–enzyme composites and MOFs embedded with active sites, and explore the utility of these catalysts in different reactions. The advantages of MOFs as enzyme mimetics are also highlighted, including confinement, templating effects, and functionality, in comparison with homogeneous supramolecular catalysts. A perspective is provided to discuss potential solutions addressing current challenges in MOF catalysis.

## 1 Introduction

Enzymes are highly evolved biological catalysts that play a vital role in biological processes and industrial production. Through accelerating the rate of chemical reactions, enzymatic catalysis enables efficient and reversible biosynthesis under mild conditions. Some enzymes such as cytochrome P450 can selectively catalyze the cleavage of inert chemical bonds, realizing essential metabolic processes such as liver detoxification.^1−4^ Interestingly, the synergy between enzymes can occur, coupling multiple catalytic reaction pathways to synthesize complex and valuable compounds. Today, more and more enzymes have been intensively applied in industries, such as pharmaceutical synthesis, detergent manufacturing, and wastewater treatment. The delicate structure and powerful functions of enzymes have fueled the intense interest of researchers. However, due to their structural complexity, the specific active sites of many enzymes are still vague and researchers have debates about the functions of components in some enzymes. In addition, the structural vulnerability of enzymes also limits their application in broader fields. Finally, how to design and synthesize artificial catalysts beyond natural enzymes is also an exciting challenge.

In order to understand and reproduce the function of enzymes, bioinorganic chemists have begun to synthesize model compounds as molecular analogues of enzymes’ active sites. In 1970, Breslow and Overman prompted the concept of “artificial enzyme”,^5^ and researchers such as Holm,^6−10^ Lippard,^11−13^ Gray,^14^ Groves,^15−17^ Rauchfuss,^18^ and Darensboug achieved fruitful results in mimicking enzymes and developed a series of highly active catalysts resembling the topologies of enzymes’ actives sites.^19−21^ To date, tremendous efforts have been devoted to assembling diverse model compounds and regulating their secondary coordination spheres. These encouraging results help uncover the mechanism of enzymatic reactions and natural biosynthesis of active sites.

With the advent of synthetic chemistry, supramolecular enzyme mimics, or synzymes, have been developed as biomimetic platforms, which take advantage of host–guest interactions to improve reaction efficiency and selectivity.^22−28^ These supramolecular catalysts feature inherent cavities or pockets to accommodate substrates and stabilize transition states, providing unique chemical environments to lower the reaction barrier. Compared with small-molecule catalysts, supramolecular enzyme mimics adopt a binding mechanism similar to natural enzyme behavior. Due to the confinement of inherent cavities, supramolecular catalysts enable intermolecular reactions akin to an intramolecular mode.

Metal–organic frameworks (MOFs) represent a new class of organic–inorganic hybrid materials, periodically linked by organic ligands and metal nodes to form two-dimensional or three-dimensional ordered networks.^29−31^ MOFs have characteristics of chemical tunability, high surface area, permanent porosity, crystallinity, and characterizable structures. Most reported MOFs are microporous materials with pore sizes smaller than 2 nm, while mesopores (2–50 nm) and even macropores (>50 nm) are sometimes presented in MOFs constructed through isoreticular expansion,^32^ topological design,^33^ and postsynthetic modification.^34−36^ Given their porous nature, MOFs are viewed as versatile platforms to encapsulate various guests, including gas molecules,^37^ organic molecules,^38^ cations,^39^ anions,^40^ and even enzymes.^41^ In addition, the structures of MOFs can be modularly engineered. With the advance in postsynthetic modifications, the organic linkers in MOFs can be readily replaced,^42^ removed, or functionalized.^34,43^ Metalation,^44^ transmetalation,^45^ and redox reaction can occur on the metal nodes,^46^ resulting in a framework with distinguished stability and reactivity. The functionalization of the organic ligands and metal nodes can further change the pore sizes and pore environments of MOFs, customizing the materials for targeting applications.

**Figure 1:**
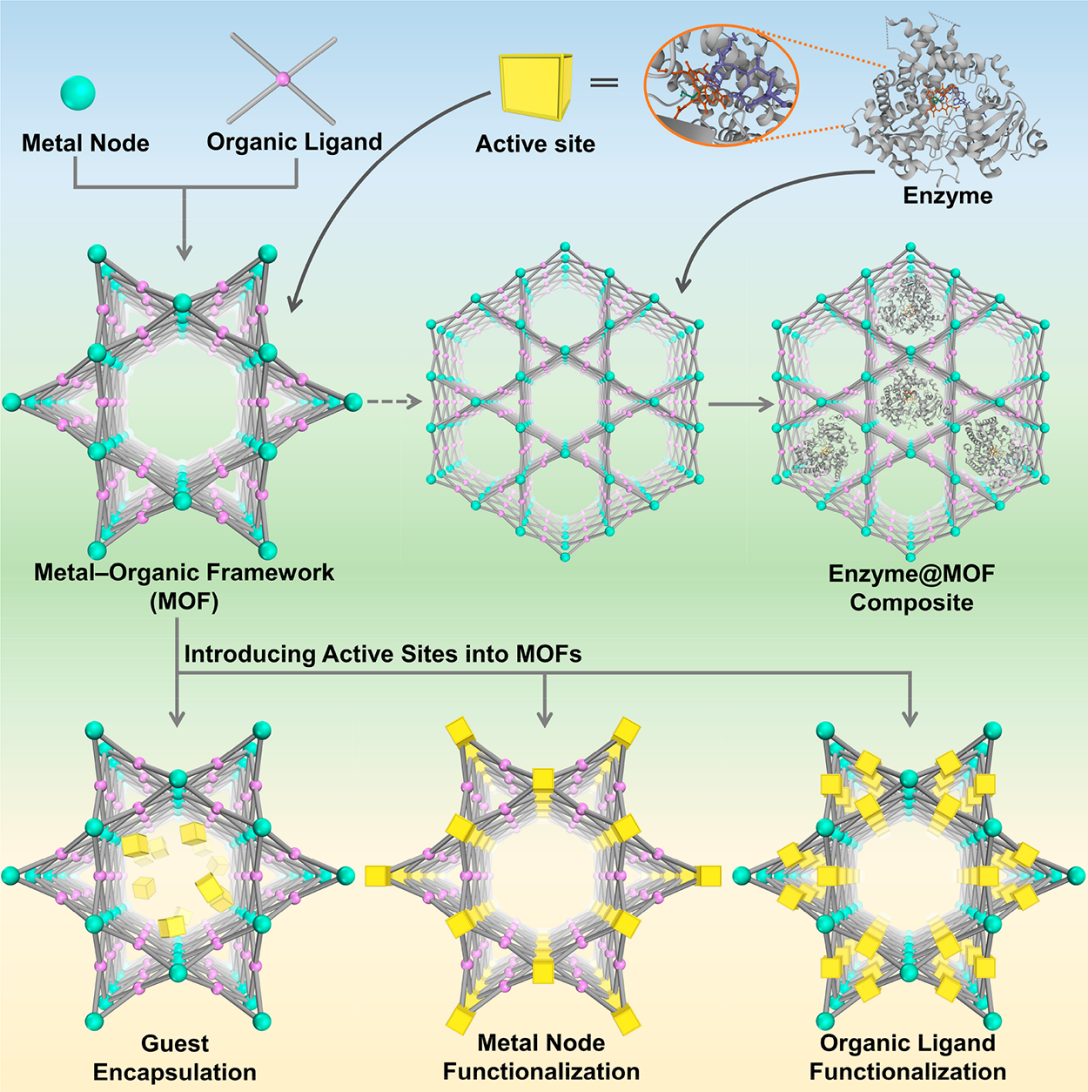
Overview of strategies to synthesize bioinspired MOF catalysts. (top) Enzymes are incorporated into MOFs to afford biocomposites. (bottom) Model compounds emulating enzyme’s active sites can be introduced into MOFs through guest encapsulation, metal node functionalization, and organic ligand functionalization.

In more than two decades of MOF development, researchers have noticed the significant potential of MOFs in catalysis.^47,48^ As heterogeneous catalysts with superior recyclability and large turnover number (TON), MOFs also feature high structural tunability and functionality similar to molecular catalysts. Taking a page from nature, diverse bioinspired MOF catalysts have been designed to reproduce or even surpass the functions of natural enzymes. In general, there are mainly two approaches to constructing bioinspired MOF catalysts, enzyme immobilization and active site installation (Figure 1). Enzyme immobilization indicates integrating enzymes into MOFs’ pores or surfaces to produce composites. Herein, the enzymes are immobilized within the framework through covalent bonding or noncovalent interactions, such as hydrophobic interactions, van der Waals forces, and electrostatic forces. The enzyme immobilization can be conducted through one-pot and postsynthetic approaches. The one-pot synthesis of enzyme@MOF composites involves coprecipitation of MOFs and enzymes under mild synthetic conditions, which enables strong interconnections between MOFs and enzymes.^49^ Yet, given the vulnerability of enzymes, the MOF scopes are usually limited in coprecipitation.^50,51^ The postsynthetic approaches embed enzymes in presynthesized MOFs, significantly expanding the types of MOF–enzyme composites in the advent of methodologies, such as surface attachment, pore encapsulation, and covalent linkage. The presence of the framework can not only maintain enzymes’ activity under harsh conditions but also allow enzymes to cooperate in a cascade. In addition, encapsulating enzymes in mesoporous MOFs can make the enzymes fully accessible to substrates, facilitating mass transfer and maintaining efficiency during catalysis. For instance, additives such as silica can be used to provide protection to enzyme in MOF, maintaining both high stability and recyclability.^28,52−55^ Compared with other materials as enzyme supporters, the programmability of MOFs in terms of ligand functionality and pore apertures could support a wide range of enzymes. Hierarchical pores within MOFs also could be harnessed to immobilize enzymes. Despite the solid and stable structure, microporous materials like zeolite might not possess pores large enough to accommodate enzymes.^56^ The limited pores would also hamper substrate diffusion. While pore apertures of mesoporous silica are large enough to encapsulate enzymes, the material’s microenvironment is required to be modified to enhance interactions to prevent enzyme leaching or denaturation.^57−59^ To sum up, MOFs provide functional and suitable pore spaces to immobilize enzymes, meanwhile advancing reusability and catalytic performances.

As emerging enzyme mimics, MOFs embedded with active sites combine the advantages of molecular and supramolecular catalysts. The porous frameworks emulate the role of the protein pocket in confining the active sites from the external environment, which provides protection as well as size-dependent substrate accessibility.^60,61^ The MOF can be considered macromolecules like the protein but with a much higher active site density. Model compounds mimicking the enzyme active sites can be introduced into MOFs mainly through three approaches: guest encapsulation,^62^ metal node functionalization,^63^ and organic ligand functionalization.^64,65^ In contrast to the other two approaches, the guest encapsulation demonstrates immobilizing active sites through physical or chemical adsorption, in which the framework provides a decorated and confined cavity for chemical transformations. Nevertheless, aggregation and leaching of active sites are often encountered with such an approach. Functionalization on metal nodes and ligands introduces active sites into MOFs’ building blocks. As a result, the infinite network of uniform pores provides rigid immobilization and spatial separation of active sites, precluding bimolecular contact and dimerization.^66^ The MOF’s crystallinity allows detailed structural properties to be determined, unlike amorphous solid-supports like polymers, silica, and alumina, where the structural disorder hinders the identification of catalytic intermediates. MOFs provide a modular crystalline environment to potentially identify and characterize reactive intermediates in one unified system, where previous mechanism studies are highly fragmented among a diverse number of enzymes. Together, the MOFs’ distinguished features describe an ideal environment for building enzyme mimetic catalysts.

This review presents a systematic summary of state-of-the-art research crossing the boundary between homogeneous and heterogeneous catalysis, leveraging natural enzymes to design innovative bioinspired MOF catalysts. Such practice includes enzyme@MOF composites and MOFs embedded with catalytic active sites.^67−71^ While previous literature provides detailed summaries of immobilizing enzymes and specific types of catalytic sites, such as porphyrin and Zr_6_-oxo clusters,^72−75^ in MOFs, the potential of MOFs as enzyme mimics and their similarities with other supramolecular catalysts have been largely underestimated. Herein, we outline the development history of bioinspired MOF catalysts and discuss the pros and cons of each synthetic approach. Additionally, the behavior of MOFs as enzyme mimics has been summarized and compared with classical supramolecular catalysts. At the end of the review, key challenges in the field of MOF catalysis were identified and an outlook for future development was provided, with the goal of advancing the fields of MOF-based catalysts.

## 2 MOFS as Enzyme Supporters

**Figure 2:**
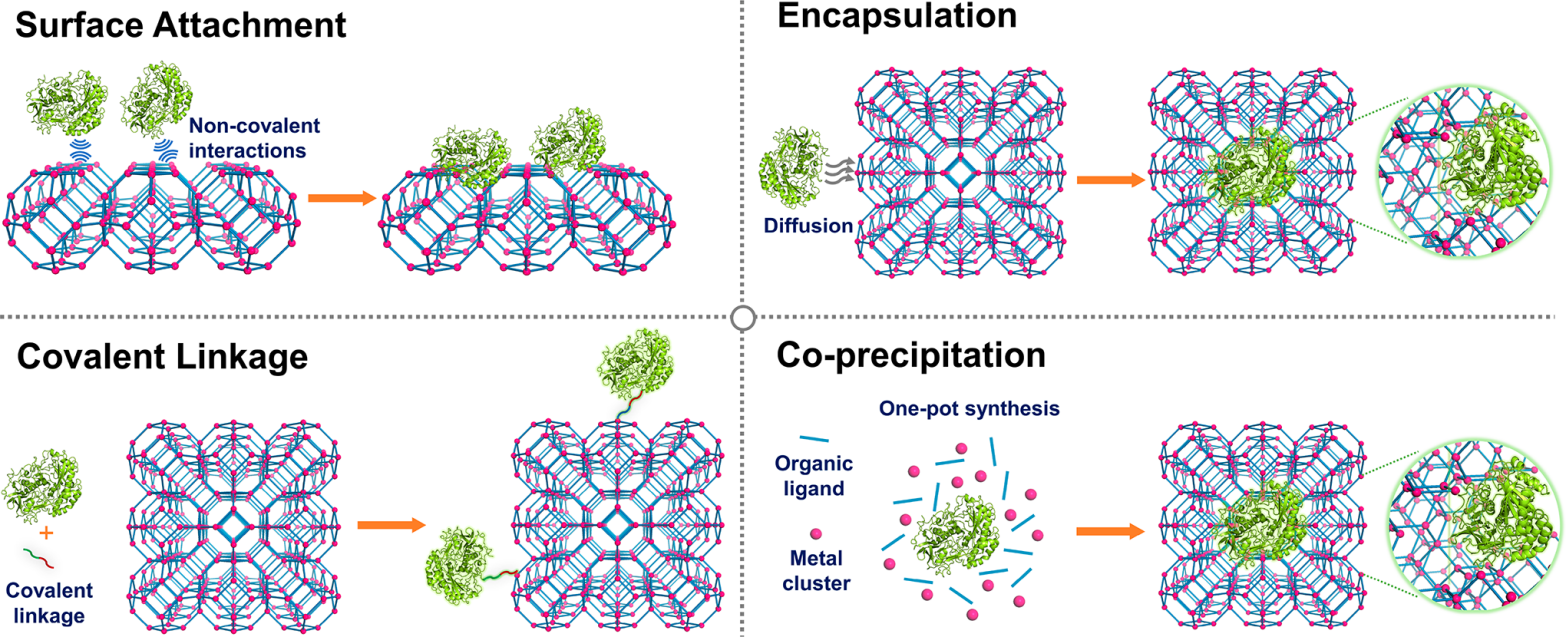
Overview of strategies to prepare MOF–enzyme composites, including surface attachment, encapsulation, covalent linkage, and coprecipitation. Surface attachment directly anchors enzymes to MOFs’ surfaces via noncovalent interactions, including hydrophobic interactions, van der Waals forces, and electrostatic forces. Encapsulation indicates entirely absorbing the enzymes into the pores of MOFs and establishing interactions within the interior environment. Covalent linkage utilizes the functional groups on both MOFs and enzymes to form covalent bonding. Coprecipitation refers to mixing up enzymes and the reactants of MOFs in the homogeneous phase, embedding the enzymes in the instantaneously formed pores.

Enzymes spread universally in the living world, displaying spectacularly efficient catalysis in biological transformations. Researchers have long been working to incorporate enzymes from living organisms and make good use of their catalytic performances.^76,77^ Because MOF has demonstrated strengths in its ordered structures, tunable porosity, multifunctionality, and outstanding chemical/physical stability, it is suitable to integrate enzymes into MOF structures and exert various yet essential reactions to carry out reactions that people can harness.^78^ Over the years, people have analyzed a variety of biocomposites and improved immobilized enzymatic performance to a large extent. The main goal of design involves expanding the roles of versatile MOFs to a fine-tuned catalysis process, which refers to tailoring exact modular construction and chemical components’ mutability.^67^ Immobilization of enzymes into MOF follows the principle that generally allows for fabricating optimized biocomposites with preserved nature and function of enzymes. Works in recent years probed deeply into the microenvironment, of which bespoke systems could perform efficient catalysis and help people utilize them in the same way that nature does it. Based on our knowledge of accessible enzymes and MOFs, both specific and generalized strategies have emerged to build the composites, bearing the requisites for stabilization and functionality, which, regarding but not limited to enzymatic performance, resistance under harsh conditions, loading quantity, and enzyme recovery.^79^ For the immobilization of enzymes with differed cofactors engaged in diverse reactions, there are reliable ways categorized into four main types (Figure 2).^41^ Surface attachment refers to using presynthesized MOFs and incorporating enzymes to their surface to form the composites, usually by weak physical/chemical interactions, which only utilizes MOF as a carrier for enzymes, to some extent. Covalent linkage approaches the enzyme–MOF bioconjugate by anchoring enzymes covalently on MOFs, enabling better recovery in general. The third method is enzyme encapsulation by tailored pores in MOFs, which focuses on utilizing physical absorption to capture enzymes and offering a suitable microenvironment for enzyme to react. Coprecipitation highlights that the enzyme is present along with the bottom-up synthesis of MOFs and the in situ formation of MOF cavities. Methods not categorized into these four main types are also available, such as immobilization from metal oxides, which serves as a mediator between the enzyme and MOF.^80^ Besides, considering the availability of enzymes in MOFs, there are meaningful and influential reactions in the biology world to be canvassed and harnessed.^69,80,81^ Significant progress has been made in conducting various reactions, such as hydroxylation, oxidation, and photocatalysis. Artificial constructions like MOFs are still far-flung from the complex yet fine-tuned organism systems.^82,83^ This part would shed light on the main types of catalytic enzymes, introducing and summarizing highlighted approaches in which multicomponent biocomposites are built, finally concluding assorted cases where diverse reactions are performed in MOFs and posting cutting-edge research.

### 2.1 Catalytic Enzymes

Of all the enzymes in nature, categorization is carried out to divide enzymes into six groups, including oxidoreductases, hydrolases, lyases, isomerases, and ligases. The division is based on the target reactions in which they catalyze. Oxidoreductases are enzymes that take on redox chemistry involving intermolecular electron transfer. Hydrolases catalyze reactions with water as an intervener to cleave substrate. Lyases directly break chemical bonds by means other than hydrolysis and oxidation. Sometimes, acid (or base) is required to cleave the substrate, reaching the reaction equilibrium. The enzymatic reactions involving two or more substrates, usually via water hydrolysis, are catalyzed by ligases. Finally, isomerases rearrange a single substrate to form the product. They were canvassing through all the catalytic enzymes, with the cofactors playing critical roles in which they directly engage in catalysis, serving as the active sites. Specific cofactors include hemes, NAD(P)H, [Fe–Fe] cluster, FADH_2_, etc. Certain metals are also involved in the active sites. Fe, Cu, Ni, Co, and Mg are universal throughout the biological world. Immobilization of enzymes in MOFs focuses on certain types of enzymes and their combinations. MOF-supported enzymes have covered nearly all types of enzymes and many reactions.^78^ It is noteworthy that tricky problems were encountered when dealing with enzymes that possess strict requirements for high performance.^84^ Much more challenges are expected as researchers probe deeper into the minute scale.

### 2.2 Enzyme Immobilization

Typical interactions in terms of creating MOF–enzyme biocomposites are classified into four groups, surface attachment, covalent linkage, enzyme encapsulation, and coprecipitation. These methods altogether pave the way for further functionalization toward biocatalysis on MOFs. The enzyme@MOF is a mutual platform where each component will affect the other in certain ways. Apart from supporting enzymes, MOF can alter the environment around the enzyme; therefore, the choice of MOF has to be canvassed through to protect enzymes and facilitate catalysis. Likewise, enzymatic reactions involve not only the enzyme but also the reactants/products, which can be largely influenced by the pore size and hydrophilicity/hydrophobicity of MOFs. Therefore, features from both enzymes and MOFs are critical to constructing an active and recyclable catalysts, and any details involved in the synthetic conditions and reaction conditions should be explicitly reviewed.^85^

#### 2.2.1 Surface Attachment

This method directly anchors enzymes to presynthesized MOFs via noncovalent interactions, including hydrophobic interactions, van der Waals forces, and electrostatic forces, to immobilize and stabilize enzymes on the surface of MOFs, which can also be termed as “surface bound”.^68,86^ This approach generally does not require harsh conditions and robust binding between two components. Therefore, simply mixing and stirring the MOF with the target enzyme can achieve immobilization.^87^ One early example from Ma et al. specified using ZIFs to anchor glucose dehydrogenase (GDH).^88^ These researchers also compared different ZIFs on the performance of adsorption capabilities, where they found that ZIF-70 had the largest capacity. In this case, GDH is physically attached to the surface of ZIFs through a simple agitation that relies on noncovalent van der Waals and electrostatic forces. As a result, GDH on ZIFs was introduced as a biosensor with high selectivity toward glucose, which showed outstanding potential for sensors in biological systems. Such an immobilization approach relies more on physical interactions to achieve absorption, and similar cases can be found in other MOFs, such as ZIFs,^89,90^ MIL-53,^91^ MIL-100,^92,93^ Cu-BDC,^94^ and UiO-66.^91,95,96^

**Figure 3:**
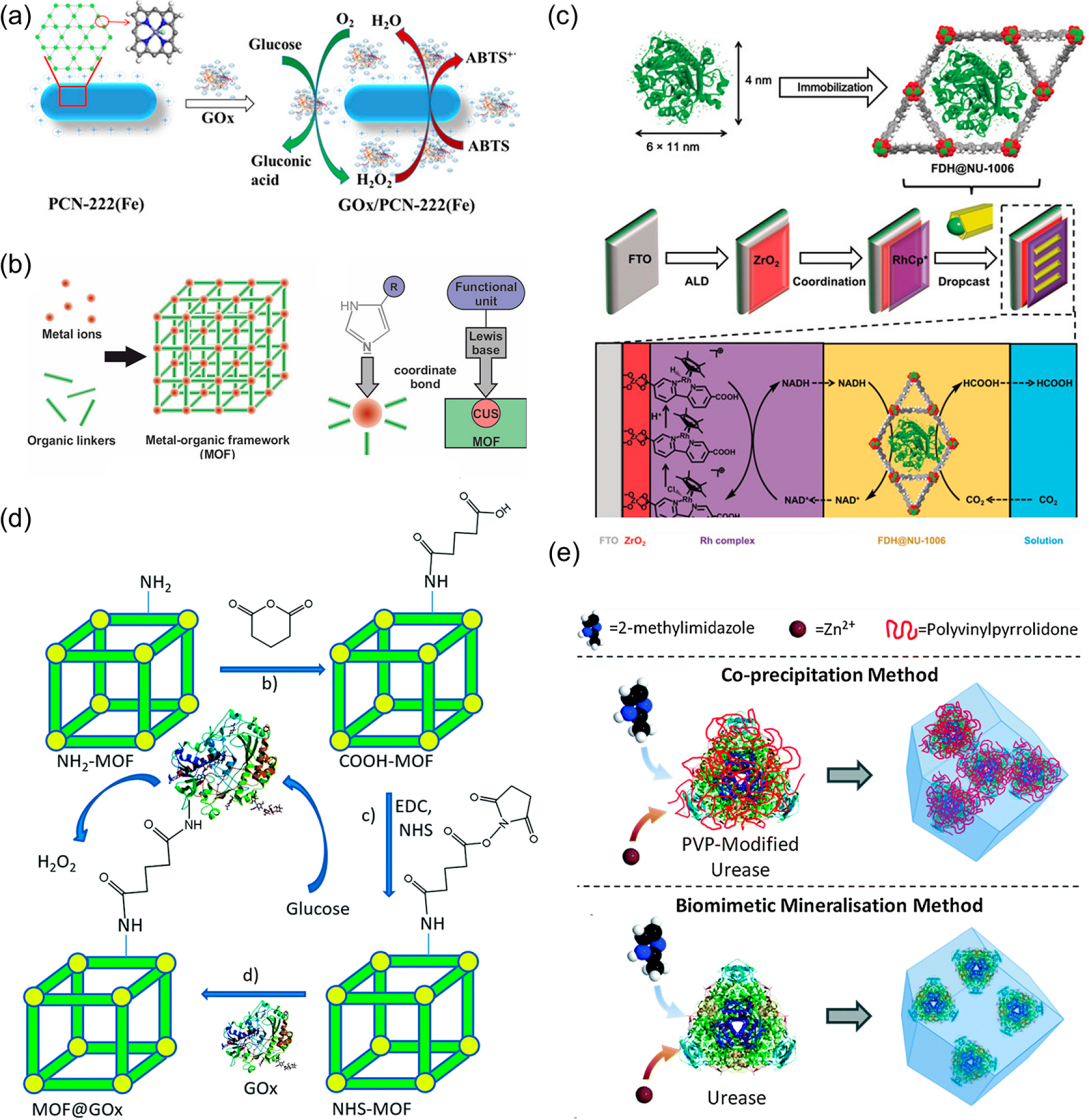
Schematic illustration of enzyme immobilization methods in MOFs. (a) PCN-222 as the supporter for immobilization of GOx by electrostatic interaction. Reproduced from ref (100). Copyright 2019 American Chemical Society. (b) The coordinative bond between the imidazole group from MO act as Lewis base and coordinatively unsaturated metal sites (CUS) acting as Lewis acid in immobilization. Reproduced from ref (106). Copyright 2017 American Chemical Society. (c) Enzyme encapsulation where formate dehydrogenase infiltrates into the pores of NU-1006. Reproduced with permission from ref (141). Copyright 2019 John Wiley and Sons. (d) Covalent linkage via *N*-hydroxysuccinimide to immobilize GOx on NH_2_-MIL-53(Al). Reproduced with permission from ref (112). Copyright 2016 Royal Society of Chemistry. (e) Illustration showing coprecipitation and biomimetic mineralization via a one-pot synthesis to immobilize urease in ZIF-8. Reproduced with permission from ref (153). Copyright 2016 Royal Society of Chemistry.

Besides Van der Waal interactions, existing electrostatic forces and other noncovalent forces also demonstrate stable immobilization of enzymes in conjunction with other linkers.^97,98^ Examples such as certain amino acids displayed on the outer surface of enzymes with abundant charges keep them solvated in an aqueous environment, which can be utilized to interact with metal ions, providing long-distance stabilizing force^99,100^ (Figure 3a). Introduction of polydimethylsiloxane (PDMS) to ZIF-8 provides a hydrophobic environment where hydrophobic molecules such as biodiesel can be produced with the help of *Aspergillus oryzae* lectin (AOL).^101^ Factors influencing the electrostatic microenvironment, such as pH and function groups in amino acid residue, can be fine-tuned to improve the enzyme’s catalytic performance to a large extent.^102 ,103^ In addition to that, additional linkage can form with desired charges where there is a need for anchoring. Numerous cases following this principle have been reported, including the pioneering research by the Kumar group,^104^ which has utilized tetraethylenepentamine (TEPA) as an addictive linker on aspartate and glutamate residues to provide reverse charges. The amino acids function as joints to anchor TEPA. Further comparison with the original charges of the enzyme demonstrated that this method had an affinity about 3.5-fold higher than the original enzyme. Based on this principle, other small molecules regarding reliable electrostatic interactions have been reported as well. In 2013, the Huang group reported using a trypsin–FITC combination to build a bioreactor with higher efficiency by introducing the dye molecule into the pore of MOFs.^105^ A relatively strong π–π stacking force confers the enzyme with stable and universal anchoring throughout classical MOFs. Surprisingly, they found that the FITC did not interfere with the high ingestion performance of trypsin. Using enzyme tags like FITC for stabilization could not only hinder the leach-out of enzymes from MOFs but also be a rapidly conjugated process that diminishes cumbersome preparation. Extended from this general idea, manifold conjugated dye linkers have been introduced to enhance its effectiveness. Histidine could be used to form coordinative bonds to integrate enzyme on MOFs. The imidazole group provides sites to form a Lewis acid–Lewis base pair to immobilize THE enzyme.^106^ (Figure 3b) Another case reported by Huang, Lin,^105^ and co-workers unveiled the NBD as a multipoint anchoring linker connected to the trypsin, which produced both stronger binding toward UiO-66 and higher proteolytic efficiency than other coupling linkers like FITC.

In some other cases, hydrophilicity and hydrophobicity of enzymes bolster the durability of biocomposites as well,^107^ this is often focused on the supporter, however. Specifically, Zhao and co-workers connected the Fe_3_O_4_ particle and HKUST-1 MOF particle with the help of polydopamine (PDA).^107^ PDA not only tunes the environment to hydrophilic but also protects the Fe_3_O_4_ core from acidic conditions. The Doonan group capitalized on fluorescence tags to study the different chemistry environments on the MOF surfaces and their impacts on enzymatic activity. Sharing topologically identical morphologies, nevertheless, MAF-7 and ZIF-8 differed in the surface for water affinity, which directly diminished the catalase–MOF immutability of the latter.^50^ Hydrophobicity in this example played a crucial role in the inactivation of the enzyme. On the contrary, hydrophobicity was confirmed as an advantage when dealing with certain enzymes. In the case of catalase, the researchers found augmented overall integrity of the system. This phenomenon resulted from hydrophobicity favoring stacked dye conditions from high ionic strength, proffering us the knowledge that a customized microenvironment is necessary for both the enzymes and MOFs for surface attachment because merely physical absorption without modification set many limitations to implement sundry enzymes.

#### 2.2.2 Covalent Linkage

It is conspicuous that either physical or noncovalent interactions are insufficient to accommodate enzymes. However, because both MOFs and enzymes have displayed abundant potentially modifiable groups, the utilization of those functional groups to form covalent bonding becomes liable. Employing strategies like these enables scientists to introduce organic reactions into the field of enzyme immobilization, propounding tools for increased recyclability and reduced protein unfolding, accordingly.^41^

An aboriginal example is from the Park group in 2011,^108^ who initially used *Candida-antarctica* lipase-B and enhanced green fluorescent protein (EGFP) to form conjugated MOFs. This strategy made good use of the linker 2-amino–1,4-benzene dicarboxylic acid (NH_2_–BDC) for different constructions. DCC here showed its unique property as an intermediate linker to create the chemical bonding between the two main motifs. It is worth noting that physical interactions are ignorable in this case. Therefore, covalent linkage stands out to be the candidate. The Park group decorated CAL-B on the 3D-MOF to form dual protein-conjugated biocomposites and surpassed free CAL-B and other inferior MOF-based supporters profoundly with boomed activity and selectivity. Under the confirmation of fluorescence microscopy, solid-state luminescence measurements, and confocal laser scanning microscopy (CLSM), they indicated the broadened scope in which the enzymes could work well. This approach achieved by DCC is limited in hydrophobic cases, after all, hydrophilic enzymatic reactions are restricted in aqueous solutions. Utilizing 1-ethyl-3-(3-(dimethylamino)propyl)-carbodiimide (EDC) as the functional carboxylate group can be auxiliary to carbodiimide copulation. Furthermore, the introduction of fatty acid on the linkage molecule, as reported by the Park group in 2017, showed that it could facilitate enzymatic activity under a generally unfavored polar solvent.^109^ After the covalent linkage of lipase on NH_2_–UiO-66’s surface, fatty acids with long carbon chains are anchored on the amino group of the ligand and alongside the enzyme, altering the environment nearby. Improved enzyme activity was found under an unfavored solvent compared to the free enzyme. Thus, this case demonstrates a new approach to facilitate the altogether performance of enzyme@MOF. The grafting of new molecules covalently is a determinant in creating a nonpolar, hydrophobic interaction with the enzyme.

Following analogous strategies, other cross-linkers have shown practical values in recent years, such as glutaraldehyde (GA),^110^*N*-hydroxysuccinimide (NHS),^111,112^ (Figure 3d) dibenzylcyclooctyne (DBCO),^113^ and heme.^114^ GA cross-linking, first reported by the Falcaro group in 2013,^115^ successfully connected the MIL-53-Al and the enzyme β-glucosidase. The Lou group synthesized polymerized GA to combine soybean epoxide hydrolase (SEH) and UiO-66-NH_2_ altogether,^116^ with the help of a befitting environment, the *K*_M_ (Michaelis constant) was much lower than the free enzyme. There are novel ways to introduce particular groups by specific reactions. For example, click reaction can be utilized to form covalent linkage, with DBCO serving as the mediator between azide-functioned UiO-66 and other biomolecules.^113,117^ Though this strategy has presented with widespread use and certainly improved drug delivery and other biomedical therapies, organic covalent linkage merely focused on several linkers and failed to become one of the convenient and high loading approaches. Nonetheless, the appliance of organic linkers improved interactions between enzymes and MOFs, constituting a powerful tool to broaden our scope in terms of this field.

#### 2.2.3 Pore Encapsulation

For all the cases referred to previously, surface chemistry is usually involved in enzyme immobilization. MOFs as multifunctional sustainers for enzymes demonstrate their superiority in other facets ranging from linker modification to porosity control.^69^ To further utilize the reticular MOF structure, research has focused on the enzyme absorbed into the MOF, illustrating that enzymes are entirely encapsulated in the pores of MOFs and establish interactions within the interior environment. The “pore encapsulation”, or termed as “pore infiltration”, provides enzymes with a protective microenvironment. In this process, enzymes are directly capsulated into the preformed pores of MOFs. In addition to protection, large loading performance is promising in enzyme@MOF to increase catalytic ability, and the relationship between pore aperture and enzyme scale needs to be fine-tuned.^86^ Compared with other porous materials such as silica- and clay-based materials, MOFs featured highly tunable and periodically organized structures, which played an essential part in preventing leaching and recyclability. Another advantage of MOFs lies in their tunable functionalities, of which the interior pore environment is densely decorated by metal clusters and organic linkers. With the introduction of multiple postmodification methods, the functionality of MOFs can be further modified to facilitate immobilizing enzymes. Pore encapsulation turned out to be practical in meeting the challenge.

For some pioneering research, people made good use of pore cavities of existing MOFs to conduct enzyme encapsulation. Pisklak et al. successfully encapsulated the enzyme MP-11 into a Cu-based MOF, with the linker consisting of biphenyl-4,4′-dicarboxylate (BPDC) and 1,4-diazabicyclo[2.2.2]octane (DABCO).^118^ By constructing this layered MOF with DABCO as an interconnected ligand, they formed the 3-D structure and optimized the pore environment. They controlled the cavity with a series of scales (ranging from 1.8 to 5.5 nm) and analyzed the uptake and activity of the enzyme, respectively. Activity data showed that the newly synthesized MP-11@Cu-MOF complex provided a much more axiomatic increase in methylene blue oxidation than freely dissolved enzyme. Another example from the Ma group is based on a terbium MOF with mesoporous cavities, Tb-mesoMOF, with MP-11 encapsulated inside.^67,119^ Triazine-1,3,5-tribenzoate was the linker connected with Tb ions, and altogether they formed abundant nanoscopic pores with 3.9 and 4.1 nm, slightly larger than the scale of MP-11, enabling high loading ability to 19 μmol/g and surpassed counterparts like porous silica. Besides, the Tb-mesoMOF design was able to retain considerable activity after several cycles of reuse. This early research highlighted the potential for pore encapsulation to achieve eye-catching catalytic capacities and loading quantity.

The results have spiked the interest of researchers, undoubtedly. With the help of Raman spectroscopy, the Ma group has substantiated the π–π stacking force and hydrophobic interactions between the interior of MOF and the enzyme, which were primarily contributing to stabilization. In another case of myoglobin immobilization, the same group not only demonstrated that a larger enzyme could be immobilized in the same MOFs with both higher catalytic activity toward small substrates and higher stability than the counterpart porous silica SBA-15.^120^ Meanwhile, the encapsulation of myoglobin was not as easy as that of MP-11 due to the larger size, which would induce catalytic activity decreases when met with larger substrates. The enzyme infiltration into the MOF does not indicate enzyme spontaneously absorbed into the cavity, instead, the configuration change is embodied in the encapsulation. Ma group’s research on cytochrome c (cyt c) has proved that pore encapsulation could unfold the protein, to some extent.^121^ As a matter of fact, Tb-mesoMOF has sufficient room for accommodating the cytochrome c, but the opening windows are narrower than the enzyme. Applying fluorescence spectra to analyze the amino residue on the protein unveiled that the cytochrome c adopted a configuration that was distinctive from either denatured or normal enzyme, allowing for the ingress of the enzyme. Other examples have demonstrated that partial denature could be facilitated to promote encapsulation, such as MIL-101-NH_2_ was used as a supporter for protease, the enzyme can be incubated in the mixture of TRIS buffer and hexane to induce partial unfolding.^122^ The results turned out to be competent, and enzyme functionality had been extended to a broad pH range (1–12) and temperature (up to 95 °C). Hence, the pore encapsulation is not merely a simple penetration but rather an interactive absorption.

**Figure 4:**
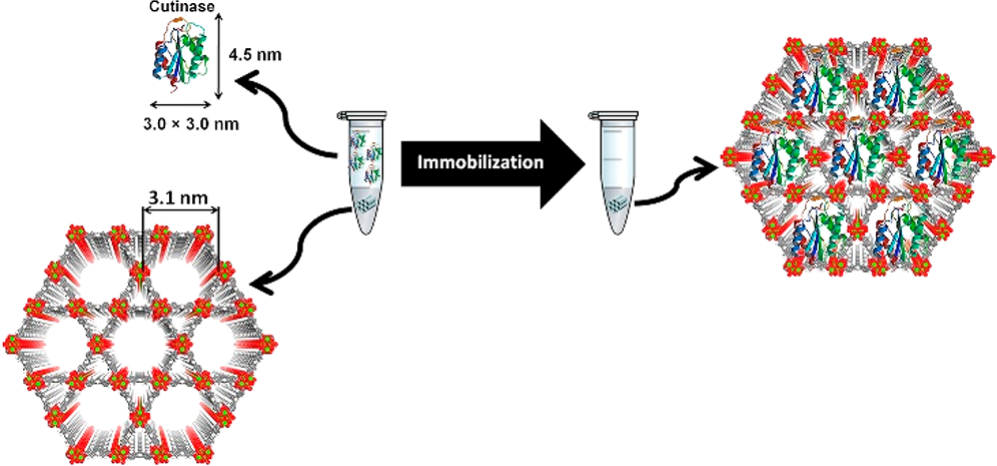
Encapsulation of cutinase into the mesopores of NU-1000. Reproduced with permission from ref (125). Copyright 2016 Elsevier.

Pore engineering is one of the main research focuses on tuning pore size and environment these days, and fabricated mesopores and macropores have been urgently demanded to study enzyme@MOF composites. The Yaghi group,^32^ the Zhou group,^123,124^ the Farha group,^125,126^ and others have reported MOFs with diverse porosity.^127−130^ To illustrate, Zhou group in 2014 utilized this strategy on MOF to embed large linkers which enlarged some pores to encapsulate enzymes of different sizes.^123^ By synthesizing similar PCN-332 and PCN-333 with differed metal clusters sharing the vertices, they successfully confirmed three different enzymes could occupy size-differed cages in one superstructure. The cages within could discriminate pores with a single enzyme and multiple enzymes, serving as a single-molecule trap to capsulate a single enzyme. As a result, horseradish peroxidase (HRP), cyt c and MP-11 all demonstrated enhanced efficiency. The PCN-888, with larger pores, was designed for the encapsulation of horseradish peroxidase (HRP) and GOX has made a nanometer tandem bioreactor possible in 2016.^124^ This work also provides a platform where substrates from the organic phase could overcome a solubility problem to meet enzymes. The Farha group used hierarchically porous NU-1000 (NU = Northwestern University) and mesoporous PCN-600 with similar pore sizes (Figure 4).^125,126^ They compared the channel difference, which could induce catalytic distinction in that substrate accessibility mattered a lot to high performance altogether with other factors. Similar results were observed in encapsulating organophosphorus acid anhydrolase (OPAA) within PCN-128Y, which was confirmed to be a well-suited scaffold for hosting enzymes. The microporous channels in PCN-128Y served as the conduits for reactant and product diffusion.^125^ More recently, the Cui group harnessed the hierarchical core–shell structures of ZIF-8, greatly enhancing the recyclability of cyt c and loading performance compared to pristine ZIF-8.^131^ A microporous ZIF-8 functionalized by magnetic particles was also reported for immobilizing catalase, from which high recovery and excellent activity were achieved compared to microporous ZIF-8.^131,132^

Driven by the quest for hierarchical porosity, researchers use the microporous MOF and postsynthetic modification well. Minor conditions change after synthesis could account for the preparation for hierarchical porosity. An example from the Kim group displayed a technique that originated from the synthesis of POST-66(Y), a yttrium-based MOF with methyl-substituted truxene tricarboxylic acid featuring high thermal stability.^128^ Water was utilized to selectively hydrolyze the ligand and create large cavities accordingly. The resultant hierarchically porous MOF was utilized to encapsulate HRP and its substrates, providing a sheltering effect in the organic solvent. Correspondingly, Zhou group prompted a linker labilization strategy, in which labile linkers were incorporated into the presynthesized MOF and then exerted under specific conditions to create a larger aperture.^34,133^ In the example of PCN-160, the linker has been changed to 4-carboxybenzylidene-4-aminobenzoate (CBAB) rather than the original azobenzene dicarboxylate (AZDC) but on the same scale. Specifically, a labile linker was incorporated in PCN-160, which was presented with numerous larger pores after mild acid treatment. The hydrophilic environment within and up to 18 nm pore size altogether makes it a promising method to immobilize enzymes. Another case focused on using hierarchically porous MOF showed resistance toward enzymes in terms of eliminating the influence of inhibitors. Gastaldo and co-workers, in 2019, reported the encapsulated protease in MIL-101(Al)-NH_2_, where multiple hydrogen bonds were present to provide stability for the enzyme.^122^ As a result, the protease@MIL-101(Al)-NH_2_ showed activity from pH 1–12 and heated up to 95 °C. When a competing enzyme was present, it could not enter the smaller pores of MOF and was therefore protected from the protease. Altogether, this hierarchically porous biocomposite proved its compatibility under two competitive enzymes. In addition, generating different encapsulation patterns regarding the enzymes’ positions in MOFs are critical as well.^134,135^ Influences on the enzymatic performance from mixed phases and pore environments have been probed into, illustrating the essence of pore sizes in facilitating substrates transfer.

Much research has been pulled out in recent years, canvassing a large scope of valuable enzymes and immobilizing them by pore encapsulation, typical enzymes including β-glucosidase (BGL),^136^ catalase (CAT),^137^ lipase,^94,138,139^ cutinase,^125^ oxidase,^124,136^ anhydrolase,^125,140^ and dehydrogenase^126,141^ (Figure 3c). Considering the fact that enzyme filtration and relatively harsh conditions of MOF synthesis are separated in time and space in the method of pore encapsulation, further detailed tailoring and tuning of the encapsulation could be illustrated. In addition to that, cutting-edge techniques like microfluidics and electrospray in terms of MOF synthesis demonstrated their superiorities as well in enzyme encapsulation.^136,142^ Nonetheless, the biocomposites built for entrapped enzymes could be hindered by the limited mass transfer of macromolecule substrates. With the goal of fine-tuned chemistry inside pores, advanced applications are reachable in many ways.

#### 2.2.4 Coprecipitation and Biomineralization

One alternative pathway for enzyme immobilization is taking advantage of the relatively mild synthesis of MOFs, which values the *in situ* localization arrangement of the enzyme within the superstructure. While pore encapsulation stresses on pore microenvironment to attain better accommodation of enzymes, coprecipitation focuses more on the so-called “one-pot” synthesis. This method refers to the reactants of MOFs mixed with enzymes in a homogeneous phase before the construction of the framework. During the building of MOFs, enzymes will be confined into nanopores of MOFs, where they are physically entrapped inside. In some literatures, “coprecipitation” is also depicted as “encapsulation”.^67^ The ratio and concentrations of the MOF precursors can be varied depending on the enzymes to be immobilized. This fact can lead to diverse MOF structures and pore environments.^143^ Generally speaking, the coprecipitation approach here can be divided into precipitation and biomimetic mineralization, of which the difference originated from whether it involves additive chemicals. Basically, the method is featured directly synthesizing enzyme-embedded MOFs and facile reaction habitat, which is also occasionally present with additives to enable the functionality of enzymes on an even keel. The initial work studying this mechanism is from Liu group in 2014,^49^ a cyt c@ZIF-8 was fabricated in a homogeneous mixture of zinc nitrate hexahydrate, 2-methylimidazole, polyvinylpyrrolidone (PVP), and cyt c. The product from this one-pot synthesis was also verified by SEM and TEM after the removal of enzymes, displaying cavities ranging from 5 to 20 nm, yet in ZIF-8 the average pore size did not exceed 1 nm. The 10-fold enhancement of catalytic performance for cyt c compared to free cyt c and similar results in other enzymes like HRP and lipase hold promise for the further development of this method. It is convincing that incubating cyt c with methanol and the presence of zinc ions have also boosted the reaction. PVP serves as the stabilizer for enzyme dispersion in methanol, protecting its functionality at the same time. This strategy has risen to be a facile method to integrate enzymes with MOFs naturally.

While chemicals like PVP plays conducive roles in one-pot synthesis to proffer diffusion of enzymes, which could help increase activity by a considerable percentage, biomimetic mineralization, could be employed to construct biocomposites as well with simply proteins as seeds for construction in the absence of facilitators. Another pioneering work by the Liang and Falcaro group reported the first example of unprecedented biomacromolecules encapsulated in the MOF synthesis and the latter forms coatings for the biomolecules^144^ (Figure 3e). An array of enzymes and proteins has been “mineralized” into MOFs such as catalase,^50,137,145−147^ horseradish peroxidase (HSP),^148,149^ bovine serum albumin (BSA),^150^ and ribonuclease A.^151^ As the synthetic procedure proceeded, enzymes inside modulated the size and morphology of cavities and the latter established strong interconnected interactions within the biomacromolecules inside concomitantly. Manipulation of MOF precursors in terms of different concentrations can introduce different morphologies as well.^51^ By taking advantage of the self-adjusting enzyme in MOFs, researchers tested the enzymatic reaction under harsh conditions for the enzyme, such as high temperature and denaturing solvent.^144,146^ It turned out that most of the enzymatic catalytic performance could be preserved. This fact encouraged further research that the natural immobilization of enzymes could fine-tune the structure, as well as the structure could influence the enzymes embedded. Similar results from Shieh group have verified the MOF’s robust yet size-matched window for accommodating the catalase allowed for increased recyclability and stability.^152^ They demonstrated the *de novo* approach to entrap several small enzyme molecules in large pores. It is shown that proteinase K did not have access to the detriment of the catalase protected inside.

**Figure 5:**
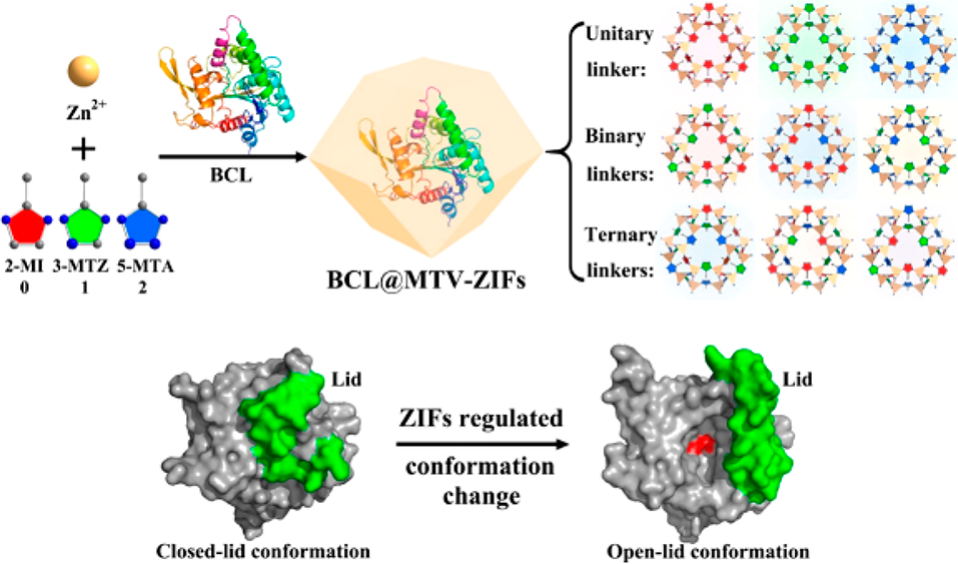
One-pot synthesis of BCL@MTV-ZIFs, in which the closed-lid/open-lid conformations of BCL were regulated via MTV-ZIFs. Reproduced with permission from ref (138). Copyright 2021 American Chemical Society.

It is crucial for researchers to interpret enzyme behavior within a spatially confined environment. The Doonan group has conducted coprecipitation featured in controlling hydrophilicity of the microenvironment by utilizing ZIF-8,^153,154^ ZIF-90,^50^ and MAF-7 (MAF = metal-azolate framework) to load FITC-tagged CAT (FCAT). While ZIF-90 and MAF-7 create a hydrophilic environment inside, ZIF-8 was known for its hydrophobicity instead. The hydrophobic environment can engender conformational change to the enzyme and therefore decreasing its activity.^155^ Additionally, unwanted aggregation of enzymes can also happen in a hydrophobic environment. After synchronous incubation of FCAT, reaction rates were determined, and ZIF-8 hardly had any decomposing effect on the substrate hydrogen peroxide, while the other two biocomposites showed close capability toward free enzymes. Here hydrophobicity also obstructs substrate and product diffusion along the cavity of MOFs. Discrimination of elastic effects in MOF cavities and the exact host–guest interactions are both crucial to actual performance via coprecipitation for enzyme@MOF composites. The cavity of MOF can be utilized to control the enzyme within and keep their high catalytic abilities. As reported by the Chen group, cytochrome c was immobilized through a one-pot synthesis of a MOF called NKMOF-101.^156^ By using harsh conditions such as heating, organic solvent, and trypsin degradation, they found the cyt c@NKMOF-101-Zn proved to be the best candidate to protect cyt c. Circular dichroism indicated cyt c could be well-protected in terms of its secondary structure. Altogether, the characterization demonstrated the small cavity and metal ions from NKMOF-101 can synergically provide a suitable environment for the enzyme to boost catalytic ability. Meanwhile, upon coprecipitation of MOF and enzyme, it can help to reduce impurities generated through the catalytic process. The Zhao group reported a coprecipitation between α-glucosidase (GAA), GOx, and Cu-MOF in 2019.^157^ The approach allowed less impurity compared to free enzyme during the catalysis. Simple centrifugation can remove the product from GAA@GOx@Cu-MOF, indicating the recyclability of enzyme@MOF in catalysis. Harnessing the cavity size can also be helpful for increasing the selectivity of substrates. In 2019, the Luo group coprecipitated ZIF-8 with a lipase called *Candida rugosa* lipase (CRL), where they examined substrates with different lengths of carbon chains.^158^ While larger substrates were constricted on enzymes at the surface, smaller substrates can diffuse into the micropores of ZIF-8. Therefore, this case indicates the versatility of enzyme@MOF for catalysis, where the sizes of substrates can induce different positions of catalytic reactions. Another case demonstrates control of the cavity environment has been achieved by the Cheng group,^138^ who continuously tuned the hydrophilicity in the pores and depicted a clear illustration existing in nano-*Burkholderia cepacia* lipase (nano BCL) and ZIF-8 that a specific sequence of arrangement resulted in the switch of the enzyme conformation (Figure 5). Multiple linkers have been studied for activity performance, and a minor change in linker sequence could cause a significant decrease in activity. With precise control at a molecular level, superstructures bestow catalytic capability in other ways. Hence it is reasonable to view single pore and whole architecture as inalienable considerations.

One-pot coprecipitation has superiority not only in the improved ability of catalysis but also in equipping biosensors as well. Dong group designed a ZIF-8-based sensor, where GOx and NiPd hollow nanoparticles were immobilized.^159^ Moreover, the composite has outstanding electrochemical sensitivity performance toward glucose, which has the potential to be applied in colorimetric sensing. Another aspect refers to the biocompatible immobilization of agents such as insoluble biopolymers and cotton fibers.^160^ These guests improved MOFs in mechanical strength and controllable crystallization, which originated from coordination with zinc ions providing nucleation sites for ZIF-8. Other components from the solution could dominate ZIF-8 morphology and enhance catalysis in addition of 0.1 M NaCl with (*R*)-1-phenylethanol dehydrogenase ((*R*)-PEDH) embedded.^161^ Meanwhile, direct nucleation triggered by the enzyme itself does depend on protein properties. The biomineralization here likely resembles pore encapsulation in which the pore environment of MOF can be tuned to accommodate enzymes to provide better performance. Certain linkers served to adjust the electrostatic potential (zeta potential) for the protein surface chemistry, identified by the Doonan group. Enzymes differ in natural pI, and electrostatic potential can determine the success of triggering ZIF-8 growth.^150^ This accounted for discrepancies regarding the formation of biocomposites. Surface chemistry modification again showed its practicality in that lysine residue and succinic anhydride facilitated the negative charge needed. Indeed, the calculation outcome from zeta potential and comprehensive 3D information corresponded. Together, they posed crucial illustrations to the biomineralization process. Similarly, work from the Ge group abandoned long-distance ordered MOF structure^145^ and instead focused on using amorphous ZIF-8 to increase mesoporous cavities and loading quantity. By optimizing the linker’s concentration in regular synthesis, they increased the average pore size to reach 5 nm rather than less than 2 nm in ZIF-8. The discrepancy accounts for the disparate difference in residual activity regarding different types of enzymes. The mesoporous generated paved the way for efficient mass transfer of glucose throughout the biocomposite to promote its dymamic detection within single living cells. Apart from enzyme biomineralization only, some auxiliary metals can be helpful to form the biocomposite. Iron mineralization, reported by Ouyang and co-workers, specified using iron as another metal in synthesizing ZIF-8.^143^ The biomineralization of GOx and introduction of iron in ZIF-8 have a synergic effect on both the enzyme loading capability and catalytic activity. Iron as a nanoenzyme can effectively degrade hydrogen peroxide generated from glucose oxidation. In this regard, the cascade reaction is formed within and faster flux rate of reactants/products. Overall, this incorporation with iron showed 82-fold increase in the activity compared to the GOx@ZIF-8.

It seems coprecipitation and biomineralization are promising strategies for the synthesis of enzyme@MOF, albeit limitations emerge in the relatively mild conditions of construction for one-pot synthesis. Facts that most biocomposites by one-pot synthesis involve frameworks such as ZIF-8 and ZIF-90 denote its narrowed scope.^50^ Shieh group recently illustrated that BGL imparted into UiO-66-NH_2_ and Zn-MOF-74, which required relatively harsh conditions to fabricate and was not accessible by traditional solvothermal synthesis, achieved by liquid-assisted grinding (LAG).^162^ A proper amount of ethanol was involved here to facilitate MOF formation. the defects existing in as-synthesized systems. Although new techniques like these are coming, they posed significant challenges for embedding enzymes synchronically with MOF. Another problem in coprecipitation is the lack of precisely spatial control. The Ge group demonstrated a method to shed light on this by microfluidic laminar flow,^142^ from which controlled defects were induced to facilitate substrates accessibility. Nevertheless, achieving control over the enzyme and MOF will invariably be the goal. Similarly, harnessing on electron microscopy can provide essential structural information with high resolution. The Chen group recently unveiled the atomic-level structures of enzyme@MOF via advanced characterization.^134^ They also specified the introduction of enzyme in MOF can cause detects and resulting in a mixture of crystalline and amorphous phases. The multiphase structure of the enzyme@MOF can proffer large open pores which turned out to be favorable for catalysis. In addition, protein surface functionalization introduces additives that act as facilitators to form a suite of biocomposites. With the porosity and chemistry corresponded, the coprecipitation could still hinder us from expected performance, though. The research in this field turned out to be the first step toward understanding the interface between MOF and biomolecules to advance enzyme@MOF systems.

#### 2.2.5 Other Approaches

**Figure 6:**
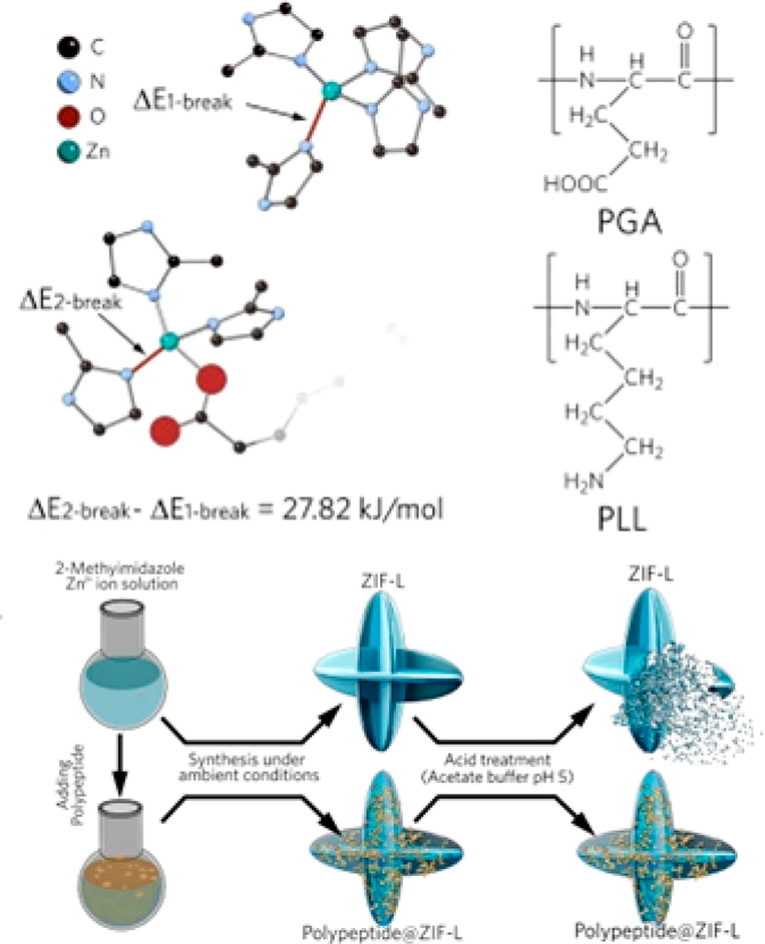
Utilization of polypeptide to boost the stability of ZIFs toward acid treatment. Reproduced with permission from ref (130). Copyright 2021 John Wiley and Sons.

The general idea of immobilization could be extended to other MOF-based biocomposites. In 2020, the Liang group harnessed on enlarging existing pores to increase the diffusion efficiency of substrates.^130^ Harsh conditions like etching by tannic acid could also facilitate macropores in MOFs, which made improvements to expand apertures in ZIF-L and thus could significantly optimize biocatalytic reactions to 16-fold (Figure 6). Besides, tannic acid coating MOF prevented it from collapsing, serving as a cooperative agent. Incorporation from other materials also imparts enzyme immobilization. Examples such as using magnetic particles as an auxiliary agent to form bioconjugate turned out to have better performance regardless of specific MOF type have emerged.^80^ Apart from the previous building techniques, the Tezcan group has reported a protein-based construction where metal ions coordinate with residues on the surface of the protein and are connected to the organic ligands.^792^ Single protein molecules were used as building blocks to form a 3D framework. This work further inspires researchers to integrate protein and even catalytic enzymes to form multivariate MOFs, therefore, performing functionalities. The Cui group recently reported a glutathione (GSH)-modified ZIF-67 to asynchronously immobilize catalase. It is worth noting that this one-pot synthesis demonstrates up to 9-fold catalytic activity compared to catalase on ZIF-67, providing a new approach to modifying microenvironment and promoting performance and stability.^163^ Throughout the research, enzyme immobilization has risen to be a versatile approach toward expected capacities and became a tool from which basic and complicated chemistry could be delved into.

Apart from all the discussions above, some review articles ought to be addressed with their distinct insights and comprehensiveness. The Lou group and the Doonan group showed detailed illustrations of the strategies used to immobilize enzymes.^67,68^ Cui and co-workers discussed how MOFs, as categorized by dimensions, affected enzymatic reactions.^69,70^ The Farha group highlighted enzyme immobilization and its use in multienzyme systems via MOFs with hierarchical pores.^71^ The Hou group examined the hierarchical MOFs with mesopores on their unique advantages and applications for enzyme encapsulation.^164^ Focusing on the environment of enzymes in MOF, the Ouyang group discussed the armor protection from MOF in many perspectives to promote the activity and application of enzymes.^165^ The Liao group explicitly illustrated the pivot advantages of MOF in constructing enzyme@MOF biocomposite.^84^ Furthermore, Liang and co-workers summarized the cutting-edge strategies for performing multienzyme cascade reactions in MOF.^166^ Other review works also contribute to the understanding of this rising research field.^167−170^ All these review articles showed that enzyme@MOF as a multifunctional platform possesses excellent opportunities and advantages.

### 2.3 Catalysis

Researchers have exploited a manifold of enzymes with multiple catalytic roles. MOFs, as the supporter for biocatalytic reactions, are assuredly the platform that meets our needs. The enzymes in organisms could be categorized into several groups based on the reaction enzyme catalyzes. Generally, the versatile approaches toward similar goals may involve disparate outcomes, which arise from minute differences and should be prudently viewed and utilized. This section reviews several classical types of biological reactions that happened on the immobilized enzymes on MOFs and posts an outlook from a synergic perspective.

#### 2.3.1 Hydrolysis

Hydrolysis refers to breaking chemical bonds and breaking substrates into smaller molecules with hydrolase as the enzyme, which has canvassed throughout the organisms, this specific type of reactions functions in diverse occasions, which has also made it particularly special regarding the required environment where it takes place. DNase and glucosidase are hydrolases that serve different roles. Researchers have performed numerous examples with glucosidase, yet hardly with DNase. Hence it demonstrates that understanding upon reaction itself ought to be coherent and explicit to be realized in MOF at its highest efficacy.

**Figure 7:**
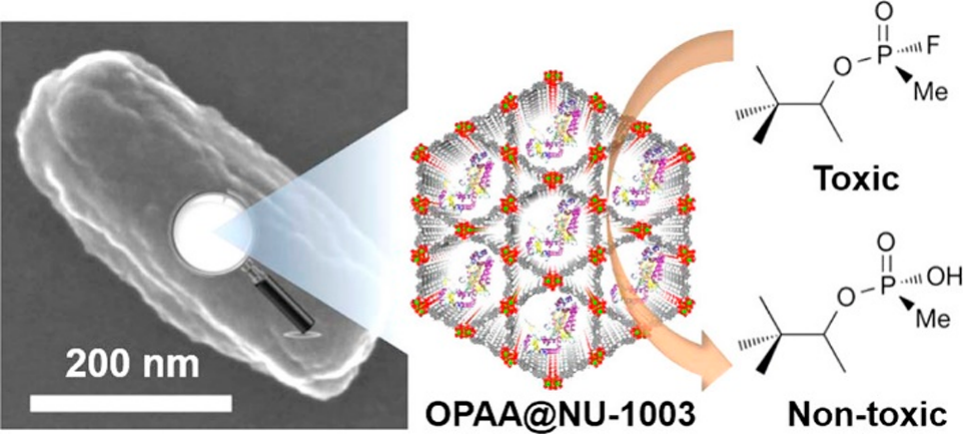
Hydrolysis reaction for chemical warfare agent degradation using enzyme OPAA encapsulated within NU-1003. Reproduced with permission from ref (125). Copyright 2016 Elsevier.

In 2015, Falcaro group did pioneer research on hydrolase.^144^ They applied the coprecipitation method to immobilize urease, an enzyme that exerts its role in decomposing urea to generate ammonia and carboxylate. Encapsulated urease demonstrated enhanced stability to heat, which could perform catalytic properties 35 °C above the denaturing point. The performances were measured by the absorptance of phenol red at 560 nm, which was introduced to the enzyme@MOF composite. The outcome not only showed stabilized enzymes with high capacity but also compared coprecipitation with PVP and biomimetic mineralization toward the same goal. The latter is proven to have better stability without PVP affecting the structure of ZIF-8. The two strategies have a promising future for a step forward to industrialized biocatalysis. In addition, cutinase encapsulation utilizing NU-1000 from Farha group exhibited excellent properties in the aliphatic esters generation via enzymatic ways (Figure 7).^125^ It turned out that NU-1000 has tunability, enabling both diffusion and stabilization of enzymes with high sustainability. Enzymatic activity was measured under various media such as tetrahydrofuran (THF) and urea, where free enzyme has decreased its capacity substantially, yet enzyme on MOFs was active.

β-Glucosidase is present when polysaccharides hydrolysis needs to be carried out by the Wang group, the mildly acid environment for this reaction could pose some challenges to MOFs.^171^ MOF-based cellulose decomposition was displayed with a Cu-MOF, and β-G@Cu(PABA) was generated. It is worth noting that one-pot synthesis was applied in this research, and the yield has reached up to 98% for degrading cellulose to glucose. In similar reactions like this, ZIF-8 may lose its robustness in the low acid stability and decompose entirely within hours. Cu(PABA) here could retain its stability against acids in pH = 5 for hours, from which its application could mainly be expanded. Later, it was demonstrated by the Shieh group that biocomposite processed by a ball milling procedure,^162^ ZIF-8 and UiO-66 both have expect-exceeding effects on harsh conditions resistance and better performance on catalysis as well. This method could also be extended to other enzymes.

Nowadays, the immobilization of hydroxylases has undoubtedly covered plenty of the enzymes of great significance in fields like manufacturing and biomedical sensors. Lipase,^94,135,172^ glucosidase,^171,173^ amidase,^127^ α-l-rhamnosidase,^174^ organophosphorus acid hydrolase,^140,175^ and soybean epoxide hydrolase are hydroxylases with meaningful uses in health monitoring functionalities that must be stable enough to fit in various working environments.^116^ Therefore, people have focused on another aspect that differed from exploring the immobilization of new enzymes. Unlike enzymes carrying out redox reactions dependent on the oxidant, the capability of hydroxylases usually depends on their substrates, along with their optimal reaction conditions. Substrates ranging from small molecules like glucose to macromolecules like protein hold different affinities for certain enzyme@MOF biocomposite. The tunability of MOFs could sustain the ongoing enzymatic reactions occurring when extended to extreme conditions. A translocation case of protease from the Marti–Gastaldo group has shown great stability under high temperatures up to 95 °C and nearly 80% activity has been recorded.^122^ Differed pH ranging from 1 to 12 has been applied and more than 50% of activity has been retained compared to the optimal pH keeping other conditions the same. The mesopores apertures and interactions from amino group shielded enzyme that relative stability under such a highly intense environment could be maintained.

#### 2.3.2 Oxidation

**Figure 8:**
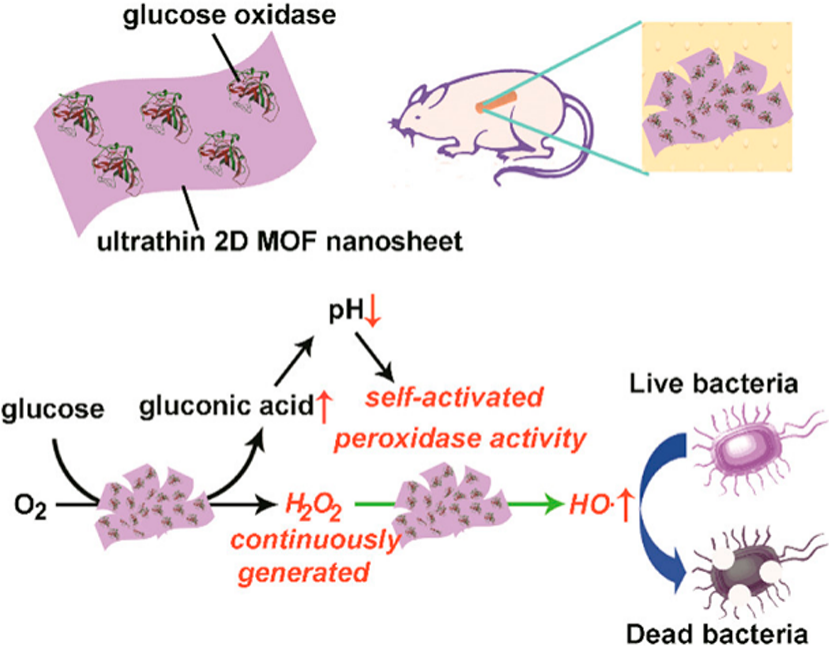
Glucose oxidase immobilized on 2D MOF to conduct an oxidation reaction. Oxygen is used as an oxidant to oxidize glucose to generate radicals to kill bacteria. Reproduced with permission from ref (178). Copyright 2019 American Chemical Society.

Without a doubt, oxidoreductases have been acting actively in metabolism within a biological organism. HRP,^112,176^ GOx,^100,177−179^ and cyt c are typical enzymes studied frequently by researchers that take the job for redox chemistry.^107^ HRP as a classical oxidoreductase has been immobilized in ZIF-8 via the biomimetic mineralization method by Falcaro group,^144^ where they examined the reaction of pyrogallol to purpurogallin catalyzed by HRP. It is vitally important to select candidates from other solid materials like silicon dioxide nanoparticles. ZIF-8 is a candidate which demonstrated superior ability in preventing leaching and stabilization in the harsh environment, owing to extraordinary enzyme packing. It was surprising that mineralization in ZIF-8 could sustain HRP catalytic activity to more than 80% even in boiling DMF and water. They manufactured separate materials to show the preserved activity of the occluded biomacromolecules after the removal of the framework. Their work proved biomimetic mineralization as a promising immobilization in advanced stability than relatively loose encapsulation approaches. It also heightened the level of understanding of the minute mechanism itself and brought us closer to application. In addition to that, a similar method could also be applied to GOx and cyt c by Ouyang group in 2020^178,180^ (Figure 8).

Although they all go through a redox chemistry process, the latter enzyme, together with urate oxidase, alcohol dehydrogenase, and cholesterol oxidase, take positive charges, making them resistant to interacting with zinc ions. This reduced interaction could induce slower assembly triggered nucleation. This could result in a slower encapsulation along the nucleation of MOF, during which enzymatic activity was reduced due to the interaction with 2-methyl imidazole. The one-pot synthesis is beneficial in that enzyme-triggered nucleation is present. Oxidoreductases like these mainly contribute actively to electron transfer, leading to various sensors and indicators in actual use. Comparable to ZIF-8, zirconium-based MOF has a robust structure as well, and xanthine oxidase (XO) was coprecipitated, where it oxidized xanthine to uric acid.^181^ As a biosensor with a fast response, the biocomposite held a linear response range of 0.2–40 U/L and a low detection limit of 0.004 U/L. Monitoring for trace XO is of great significance in developing XO inhibitors. Based on these important results, researchers believed this complex could be applied to actual use in sensing and early diagnosis.

Along with biosensors like that, oxidoreductase is demonstrated again as an encouraging method to place in organisms. Drug-resistant cancer cells are an issue where the target could not be reached, which could be ameliorated via *in situ* activation of prodrug under an acid environment due to the sheltering effect of PCN-333.^182^ As reported by the Zhou group, tyrosinase as the oxidase took the job to oxidize paracetamol to 4-acetamido-*o*-benzoquinone, the latter served as toxic targeting cancer cells. Gu group imparted the sarcosine oxidase (SOX) into a Zr-based MOF in which porosity could be tuned easily. To shed light on the continuous and accurate extent of mesopores required to match SOX size, a swelling agent, 1,3,5-trimethylbenzene, was added together with TCPP to construct a hierarchical mesoporous UiO-66. Notably, this was applied as a screener for early prostate cancer individuals via quantification of sarcosine. These cases have demonstrated the vast biotechnological applications with the marriage of biocompatible MOF and enzymes again. Interestingly, adsorption of substrates sometimes may pose a negative effect on the reactivity of enzyme@MOF composites. For instance, GOx immobilized in MOFs demonstrates reactive linearity according to the concentrations of glucose feed in a certain range.^159,183^ When excess substrates are adsorbed into, the enzyme will be tightly surrounded by substrates and reach its maximum capacity, leading to the deviation of its linear relationship between reactivity and substate concentration. In this case, the reaction efficiency decreased as substrates were concentrated at the vicinity of GOx, which narrowed its practical use for sensing. The adsorption effects on substrates ought to be taken into consideration when evaluating the biocomposites’ practical application.

#### 2.3.3 H_2_O_2_ Degradation

**Figure 9:**
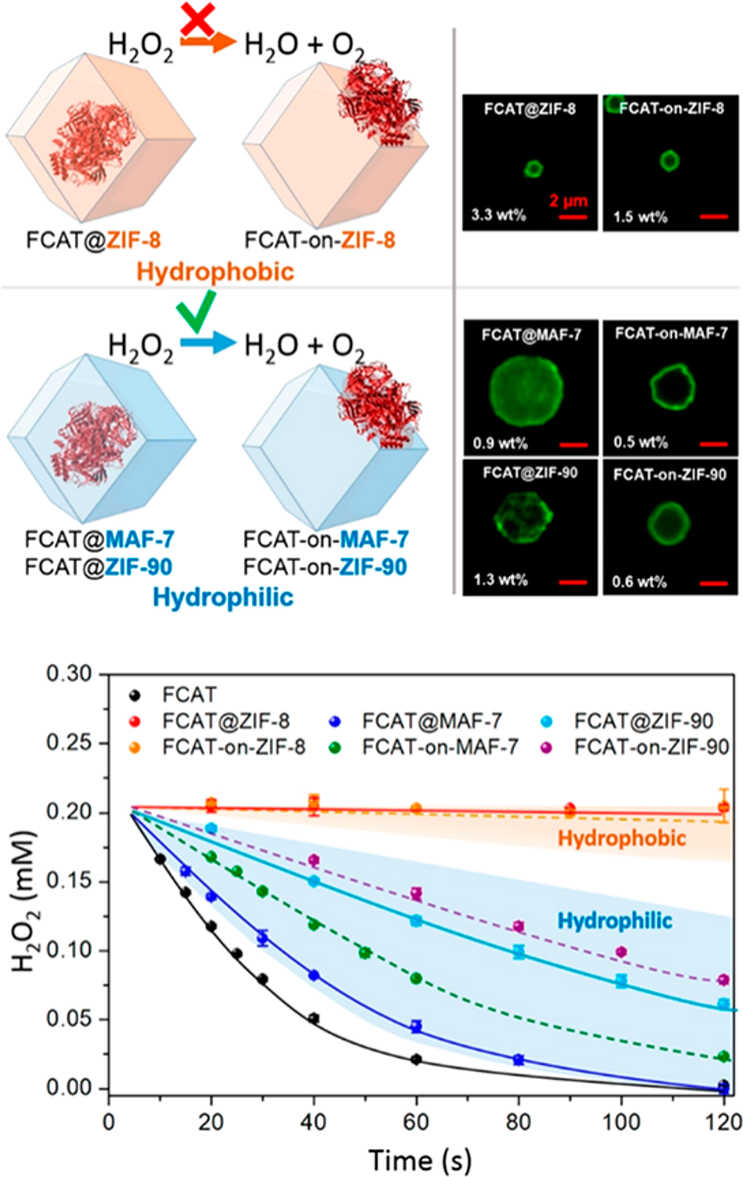
Catalase immobilized on MAF-7 and ZIF-90. The catalytic performances of catalase are presented to demonstrate residue H_2_O_2_ concentration decrease with a dependence on the time. Reproduced with permission from ref (50). Copyright 2019 American Chemical Society.

Overlapped with the oxidation section, H_2_O_2_ degradation refers to a relatively small field where catalase is commonly immobilized. The Tsung group reported the embedding of catalase in single-crystalline ZIF-90,^147^ which comprised a relatively hydrophilic environment, giving rise to stabilization from protease via a coprecipitation approach (Figure 9), following research done by the same group innovatively tight confinement of catalase@MOF in which better localization and sheltering were achieved.^146^ In this case, more permeable molecules like urea were implemented for disabling catalase and, as a result, catalase embedded showed much less decreased activity. Zeolitic imidazolate framework was again utilized as a multiple templated for different enzyme immobilization stages, which refers to a confined and relatively freestanding stage. Their attempt was ZIF-8 growth after ZIF-67 and cores of ZIF-67 were removed through a mild hollowing procedure.^152^ This hierarchically porous framework offered freestanding movements of the enzyme, accounting for nearly 3-fold activity of the confined enzyme. A larger shift in fluorescence spectra has appeared as well when treated with urea as a protein unfolding agent.

The hydrophilicity variance also impacts catalase embedded, which could result in a discrepancy in resistance for dissemblance from thermal, proteolytic, and acid treatment. The Liang group synthesized leaflike zeolitic imidazole framework (ZIF-L) to investigate acid stability in catalase@MOF biocomposites with the perspective that coordination took an active part in dual stabilization.^184^ To be more specific, zinc–nitrogen interaction by the carboxylate groups of catalases with zinc clusters facilitated ZIF-L crystallinity, especially under acidic solutions. This case enlightened that groups from the guest molecules could fabricate reinforcement symbiotically in that MOF could be maintained rigidly by introducing catalase and other biomolecules like DNA. Besides, enzymes like microperoxidase-11 and cyt c have demonstrated activity like catalase in other MOFs.^185,186^ It is natural to conclude that ZIF emerges as an up-and-coming candidate for catalase supporters as well as templates for coprecipitation and encapsulation. The size match certainly plays an essential part in it. The tunability and robustness also contribute a lot to the overall applicability.^50,142^ When it comes to specific cases, the similar scales between the MOF cavity and enzyme certainly play an essential part in the construction of the biocomposite. As the researchers probe deeper into the minute system inside, however, the acclaimed performance it may have, the distribution and mass transfer are also responsible for enzymatic capacities, which failed to be mentioned profoundly within.

#### 2.3.4 Photocatalysis

Photoreactions require the excitation of light in specific wavelengths. However, biomimetic nanoenzymes with the framework are often probed, together with the vulnerability and sensitivity of the enzymes, this type of reaction has not been broadly investigated via supporters like MOF. Usually, photoreactions are carried out more on the active center rather than immobilization of an enzyme that conducts photoredox reactions. The Chen group reported a schematic illustration of an enzyme cascade system in which two enzymes, FaldDH and FateDH, form a cascade and convert CO_2_ to formaldehyde with the help of light in ZIF-8.^187^

**Figure 10:**
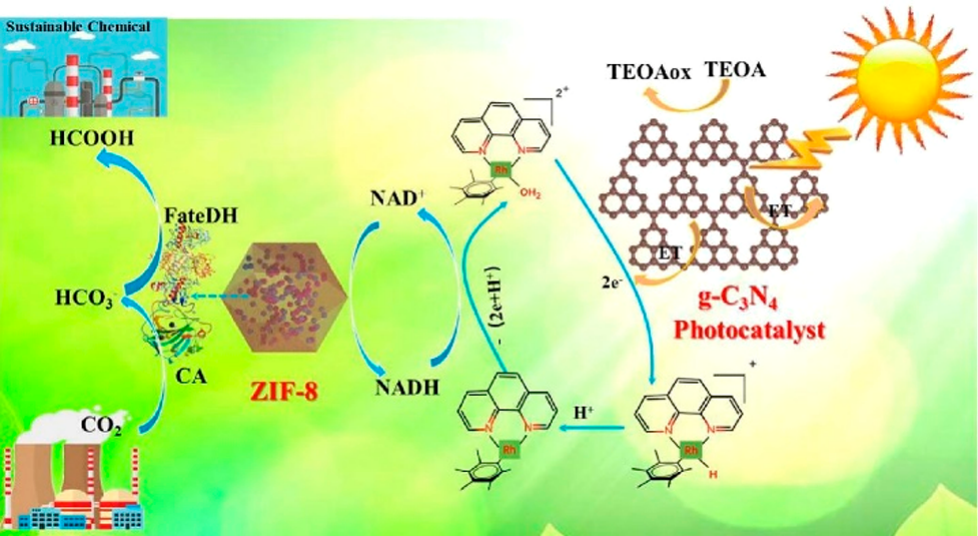
FateDH immobilized in ZIF-8 carrying out carbon sequestration under light. Reproduced with permission from ref (188). Copyright 2021 Elsevier.

During the process, NADH was used as the primary electron donor with anchored TCPP absorbing light. The group achieved higher performance than TCPP alone and free enzyme as well. They demonstrated the practical basis for integrating artificial photocatalytic systems via enzyme immobilization on MOF. The relatively low loading efficacy of TCPP suggested much room to reach perfection. In 2021, the same group published another work highlighting the combination of photocatalytic graphitic carbon nitride (g-C_3_N_4_) on the MOF, again cascaded with carbonic anhydrase as an enzyme to perform a light-involved photoreaction^188^ (Figure 10). Similar cases have emerged as well, and specific organic reaction sites must be involved to perform photoreactions.^189,190^ The previous work altogether provides fundamental insights and leaves room for future research to be carried out, proffering people with inspiring techniques to delve into this field.

#### 2.3.5 Enzyme Cascades

**Figure 11:**
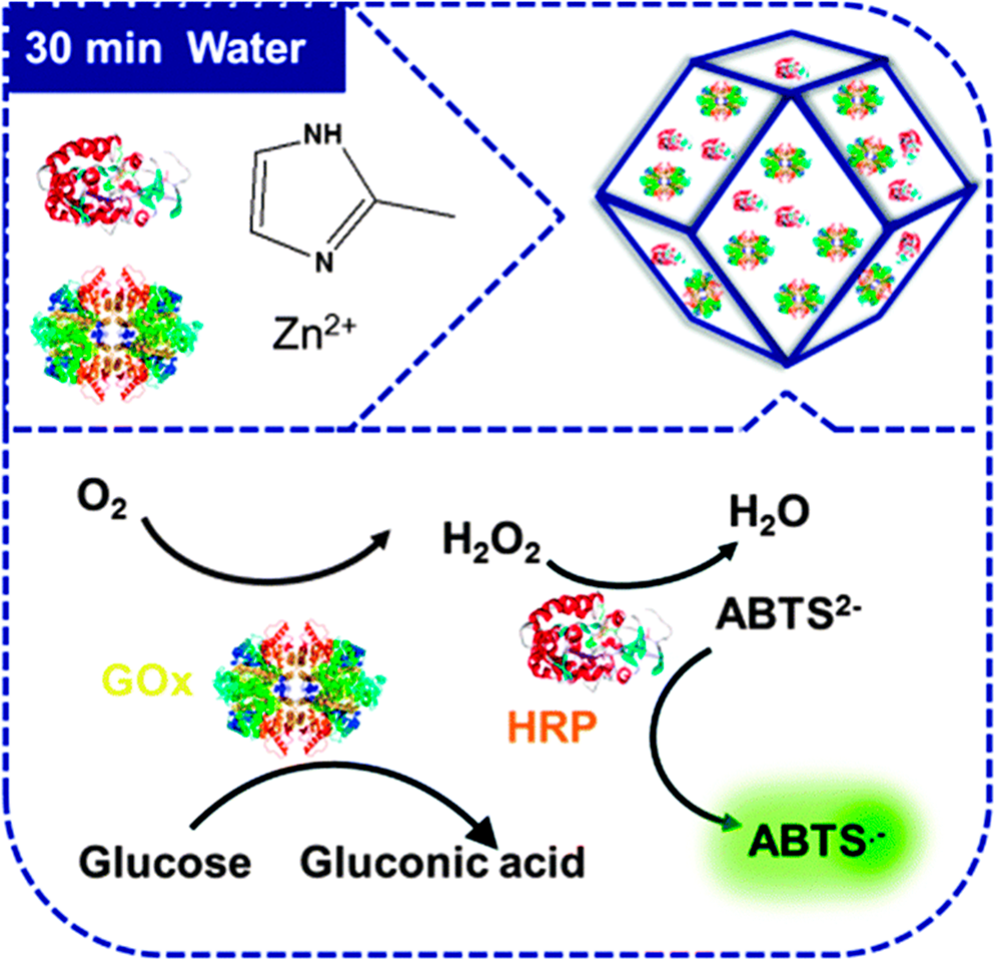
Two enzymes, namely Gox and HRP, immobilized in a MOF that together perform the oxidation of glucose and electron transport to water. Meanwhile, 3,3′,5,5′-tetramethylbenzidine (TMB) is oxidized to oxTMB. Reproduced with permission from ref (197). Copyright 2015 Royal Society of Chemistry.

Integration of multiple enzymes featured in coupled reactions is universal in organisms. Approaching the inner mechanism of biocatalytic systems also stands in need for performing cascade catalysis within MOFs. There’s no doubt that the MOF-based multienzyme biocomposite is important in advancing the depth of understanding catalysis in complex constructions.^191−196^ Regarding the fact that enzymes have to be coupled with each other in space, one of the first attempts by the Ge group investigated the coprecipitated GOx and HRP into well-studied ZIF-8 to build the artificial system in mild conditions^197^ (Figure 11). Within 30 min, the biocomposite was constructed with the cascade carrying the reaction from glucose to gluconic acid and H_2_O_2_, HRP consumed the latter to oxidize ABTS^–^. Substrate selectivity and system recovery ability were examined. It turned out that 80% of original ability was retained after 7 days, and glucose was largely consumed, contrary to analogues like fructose. Yet the method was not sufficient to perform precisely tuned enzyme localization. The following work by the Zhou group showed a cavity-dependent distribution of identical enzymes as before.^124^ Encapsulation was applied to immobilize enzymes in a stepwise order in PCN-888, realizing a hierarchically distributed biocomposite. The biocomposite with the appropriate environment has close interaction to undermine leaching. This research inspired controlled bienzyme catalysis considering the evenly distributed workflow. Techniques giving birth to hierarchical porosity could also rise from etching by tannic acid, the Liang group focused on using acid to integrate differed pores in ZIF-L and exhibited lowered surface energy.^184^ As a result, more than 2-fold boosted activity was given compared to free enzymes. The Lv group synthesized a complicated MOF structure, where an amine-MIL-101(Cr) was centered at the core and two layers of HKUST-1.^198^ This system was able to absorb CO_2_ and reduce it to formate via a three-enzyme cascade consisting of carbonic anhydrase, formaldehyde dehydrogenase, and glutamate dehydrogenase. The enzymes were separated via the two layers and this could be harnessed to reduce CO_2_ release from the inside MOF to the outer layers. Periodic generation of cofactor NADH from outside could be taken up naturally. CO_2_ as the substrate can easily penetrate the cavity of MIL-101. The MOF here also acted as adsorbent to cumulate CO_2_, which facilitated the catalysis. The system produced formaldehyde 13-fold more efficiently than the free enzyme cascade with optimization done. The same reaction was also recently reported by the Cui group, in which ion-exchange interactions can be used to tether cofactor (NADH). A nanoreactor was fabricated to demonstrate 4.6-fold yield of formate compared with free-enzyme systems.^199^ The same group also reported a bimetallic hybrid system, and MIL-88(B) Fe-NH_2_ was designed as a sensor for sensitive detection of glutamate.^200^ In addition to that, it is reasonable to harness the advantage of linker properties cooperatively with the consideration of enzymes. The Jiang group recently manifested acid-induced pyrolysis with a covalently linked enzyme system to perform productive oxidative reactions.^201^ This was a typical example in terms of biomimetic enzymes synergically connected to enzyme cascades.

Coupling enzymes with their upper hand in performing more complicated reaction cascades has proved its practical value and promising future in approaching the surface of biomimetic catalysis cascades. This has been a propelled research based on single enzyme@MOF, which undoubtedly made good use of the knowledge to sustain organized enzymes. However, the localization and spatialization of cavities and functional groups are more than significant to be probed into.

#### 2.3.6 Other Catalysis

As the matured technology of enzyme immobilization exhibited its advantages in many facets, researchers have shifted their focus to other enzymes.^202,203^ Such as thermostable *S*-adenosylmethionine synthetase (SAMS) was immobilized in nickel-based MOF (Ni-BDC) via one-pot synthesis and evaluated under high temperature and acid solutions.^204^ Kinase, another family of enzymes with pivotal roles in organisms, has also been surface attached in MIL-101-NH_2_ together with a Fe nanoparticle.^97^ After promising stability of the kinase recombinant class III polyphosphate kinase 2 (ArPPK2), they constituted a cascade reaction followed by another enzyme tyrocidine synthetase A (TycA-A). TycA-A allows for harnessing ATP in MOF-based systems. Similar cases are coming soon, which promote broader employed enzyme immobilization and sheds light on the functionality of MOFs to be a pluripotent platform.

## 3 MOFS with Enzyme Active Sites

**Figure 12:**
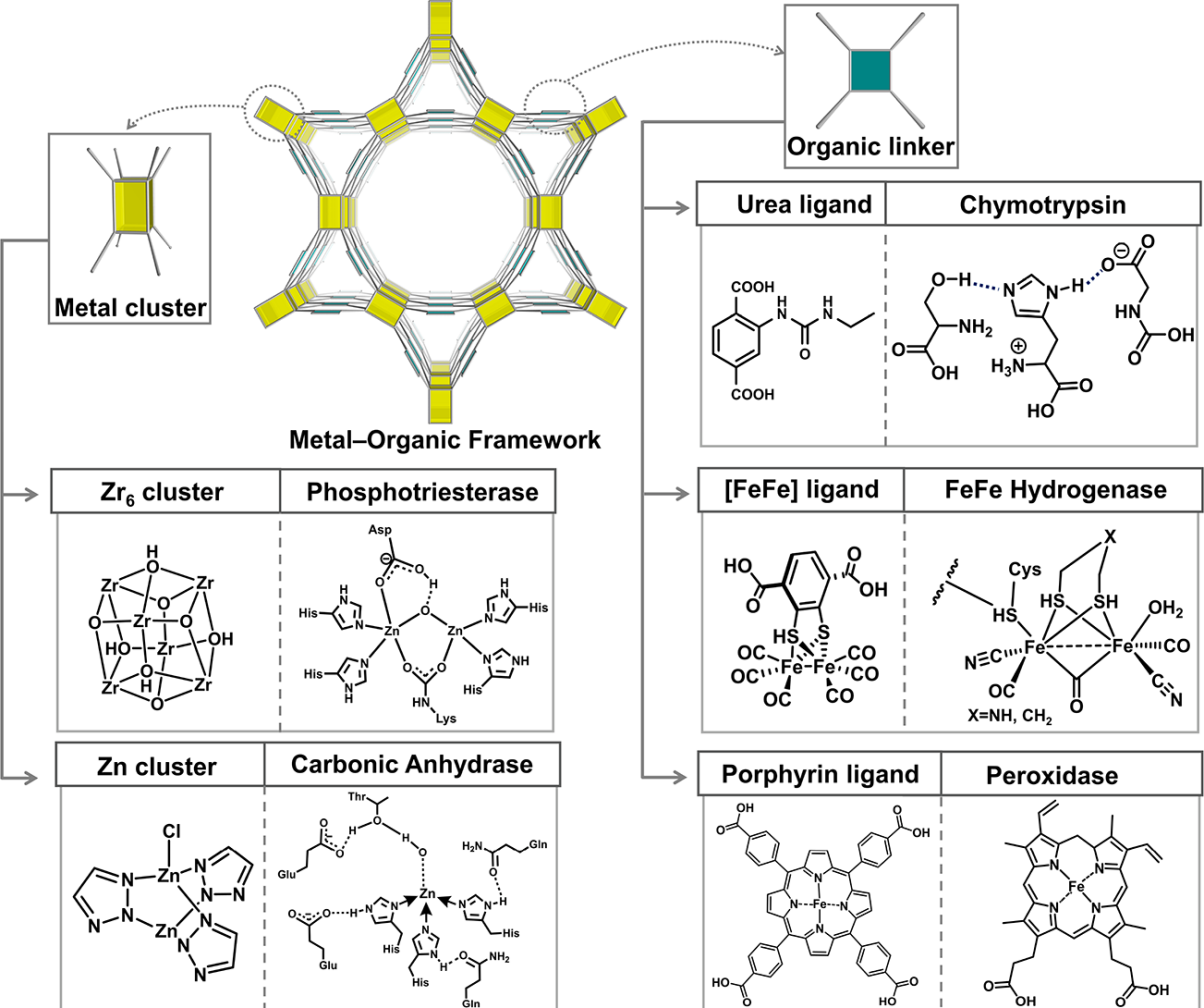
Building block design of MOFs emulating the enzymatic active sites. Model compounds are integrated into MOFs as metal clusters and organic ligands to reproduce functions of enzymes. Two representative metal clusters are Zr_6_ cluster emulating phosphotriesterase and Zn cluster emulating carbonic anhydrase. Active sites, such as urea, diiron, and porphyrin, can be embedded onto the organic linkers.

One of the most remarkable properties of MOFs is their tunable chemical compositions and tailored structures, promoted by the development of organic synthesis, coordination chemistry, and materials science (Table 1).^31,205−208^ In general, catalytic centers can be incorporated into MOFs through three approaches, namely ligand functionalization, metal node functionalization, and guest encapsulation (Figure 1).^209^ Two primary strategies have been applied to functionalize organic ligands of MOFs, focusing on functionalizing backbones and substituents, respectively (Figure 12). Herein, in the backbone design, active sites are introduced into the ligand entity, determining crucial chemical properties of MOFs, such as connectivity, pore size, stability, and topology. The substituent design mainly installs active centers as pendant functional groups onto ligands, which brings less influence on the integral MOFs compared to the backbone design. Besides, some metal nodes in MOFs feature similar structures and functions to the active centers of enzymes, which can be assembled before or during MOF synthesis. In particular, open metal sites are usually required to access substrates in catalysis. In addition, owing to the adjustable coordination modes of metal nodes, MOFs can feature diverse pore environments to accommodate guest substrates, conferring the materials with application potentials in catalysis, chemical recognition, gas storage and separation, molecular magnetism, and electrochemistry.^32,73,210−216^

**Table 1:** Summary of MOFs Embedded with Enzyme Active Sites

reaction	MOF	active sites	ref
Formate Dehydrogenase (FDH)			
CO_2_ reduction to HCOO^–^	(Me_2_NH_2_^+^){In^III^-[Ni(C_2_S_2_ (C_6_H_4_COO)_2_)_2_]}·3DMF·1.5H_2_O	[NiS_4_] core (Figure 26)	(217)
glucose oxidation to gluconolactone	[Mn_2_{Ni-(C_2_S_2_ (C_6_H_4_COO)_2_)_2_}(H_2_O)_2_]·2DMF	[NiS_4_] core (Figure 26)	(218)
Carbonic Anhydrase			
CO_2_ hydration	MAF-X25 MAF-X27	M(II) and M(III)–OH center (M = Mn, Co)	(219)
	Co-BBP@Tb-MOF	Co-BBP	(220)
	CFA-1-(OH)	Zn–OH center (Figure 30)	(221)
	ZIF-100	Zn–OH center	(222)
	MFU-4l-(OH)	Zn–OH center (Figure 30)	(223)
Nitrogenase			
N_2_ reduction	(Mo_3_ (HAB)_2_)	coordination center of Mo	(224)
	V_2_Cl_2.8_ (btdd)	coordination center of V	(225)
	MIL-53 (Fe^II^/Fe^III^)	mimicking ratio of Fe(II) and Fe(III)	(226)
	UiO-66 (Zr, Hf)	mimicking the electron transfer between P and M cluster in nitrogenase	(227)

	[Fe_4_S_4_ (BDT)_2_][NR_4_]_2_	[Fe_4_S_4_] cluster	(228,229)
	[Fe_4_S_4_ (TMBDT)_2_][TEA][Li]		
	[Fe_4_S_4_ (TMBDT)_2_][TBA]_*x*_[Li]_2–*x*_		
[Fe–Fe] Hydrogenase			
H_2_ evolution	UiO-66	[FeFe](dcbdt)(CO)_6_ (Figure 36a)	(230,231)
	PCN-222	[(i′-SCH_2_)_2_NC(O) C_5_H_4_N]–[Fe_2_(CO)_6_] (Figure 36b)	(232)
	UiO-66	[Fe_2_ (dcbdt)(CO)_4_ (PX_3_)_2_] (X = Me, Et, Ph)	(233)
	UiO-MOF-Fe_2_S_2_	[FeFe](dcbdt)(CO)_6_	(234)
	PCN-700	[FeFe](dcbdt)(CO)_6_ (Figure 36c)	(235)
[NiFe] hydrogenase			
H_2_ evolution	[Ni_2_ (PymS)_4_]_*n*_	[2Ni2S] node	(236,237)
	PCN-777	[L^N2S2^Ni^II^Fe^II^Cp(CO)]BF_4_	(238)

According to statistics, more than 90% of industrial processes use catalysts, including petrochemical, fertilizer, pharmaceutical, and plastic industry.^239,240^ According to the phase state of the reaction system, catalysts can be mainly divided into homogeneous and heterogeneous catalysts. The homogeneous catalyst works as a soluble system, including but not limited to Lewis acid, Lewis base, and transition metal complexes.^241^ The heterogeneous catalysts adopt phases different from that of reactants or products.^242^ Many nascent porous materials, such as MOFs, covalent organic frameworks (COFs), and hydrogen-bonded organic frameworks (HOFs), are recognized as heterogeneous catalysts.^243−248^ In these catalysts, the interactions with reactants, intermediates, and products with active centers determine the selectivity and efficiency in catalysis.^249−252^ Herein, the basic design principle of active sites on MOFs will be discussed, including metal nodes and ligands.

One widely applied approach to designing catalytic MOFs is to construct MOFs with open metal sites, which not only serve as single-site catalysts,^249,253,254^ but also provide the platform for structural functionalization. The open metal sites can be originated from intrinsic structures of MOFs or defects produced through postsynthetic modifications. Many MOFs are synthesized with open metal sites initially occupied by solvents or other removable molecules. In this case, solvent exchange and activation under heat/vacuum can be utilized to make the open sites accessible. Besides, open metal sites can be produced through postsynthetical removal of coordinated ligands, driven by diverse physicochemical interactions. Once the open metal sites are exposed to substrates, they can serve as the active sites for catalysis. Sometimes, the open metal sites may not feature catalytic performance solely. Herein, further structural functionalization is required, including metal exchange,^255^ linker installation and metalation,^44,256^ to endow the material with superior catalytic performance and tunable pore environment.^257^

**Figure 13:**
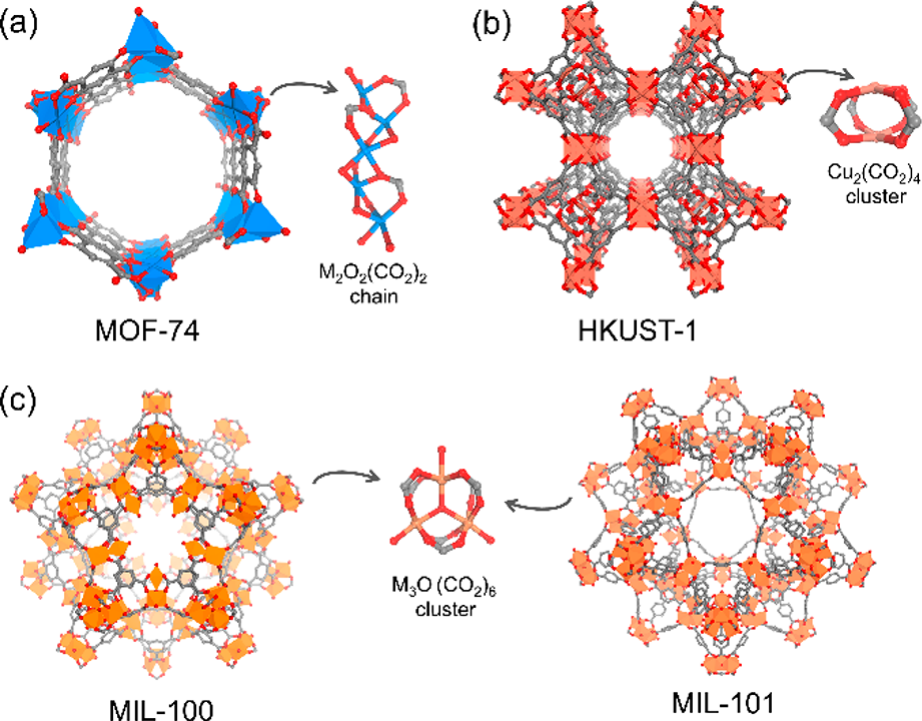
Representative MOFs with open metal sites. (a) MOF-74 containing one-dimensional channels and M_2_O_2_ (CO_2_)_2_ metal-oxo chains. (b) HKUST-1 based on Cu_2_ (CO_2_)_4_ paddle-wheel clusters. (c) MIL-100 and MIL-101 consisting of M_3_O (CO_2_)_6_ clusters.

MOF-74 and its derivatives represent an important class of MOFs with open metal sites, demonstrating exceptional gas adsorption and separation properties (Figure 13a).^32,258^ MOF-74 is composed of honeycomb pores decorated with open metal sites originating from the one-dimensional metal-oxo chain M_2_O_2_ (CO_2_)_2_. In literature, nearly all divalent metal ions can be applied as the metal source of MOF-74, conferring the MOF with diverse catalytic performances. For instance, MOF-74-Zn is known for its high performance in oxygen reduction reaction (ORR) and hydroformylation.^259,260^ MOF-74-Ni enables catalyzing the Suzuki–Miyaura cross-coupling reaction.^261^ In addition, multiple metal cations can be doped into MOF-74 to generate mixed-metal MOFs for catalysis. In 2017, MOF-74-CoNi was synthesized as a highly efficient electrocatalyst for water splitting,^262^ while MOF-74-NiCo and MOF-74-NiFe were employed for the oxygen evolution reaction.^263^ A recent report demonstrates the catalytic activity of MOF-74-NiMg for carbon dioxide capture and methanation.^264^

HKUST-1 is another classical MOF with the Cu_2_ paddlewheel cluster (Figure 13b).^265^ Owing to the robust nature of Cu_2_ cluster, HKUST-1 can remain stable in aqueous solutions. The open metal sites on the Cu_2_ cluster enable selectively bonding and transforming substrates, yielding high catalytic activity in low-temperature CO oxidation,^266^ electrochemical CO_2_ reduction to hydrocarbons,^267^ and hydrogen evolution reaction (HER).^268^

MIL-100 and MIL-101 are typical MOF examples with large pores and exceptionally high stability, allowing structural functionalization under harsh conditions (Figure 13c).^33,269,270^ The open metal sites of MIL-100 and MIL-101 are located on the M_3_O cluster, providing the docking sites for modification. For instance, MIL-101 has been used to catalyze methanol synthesis from CO_2_ hydrogenation^271^ and the aerobic oxidation of benzyl alcohol.^271^ Kim and co-workers installed chiral ligands onto the open metal sites, converting the MIL-101 into a homochiral MOF with remarkable catalytic activity in asymmetric aldol reactions.^272^ Besides, some recent reports indicate that catalytic metal nanoparticles can be incorporated into MIL-100, which occupy the large cavities or defects in the MOF.^273,274^

In addition, the ligand functionalization in MOFs can be divided into two aspects, backbone and substituent. The substituents on ligands of MOFs can be readily modified by pre- or postsynthetic methods. As an example of the presynthetic method, one of the most studied ligands, terephthalic acid, is easily modified with multiple functional groups to confer diverse properties on the resultant MOFs.^275^ In 2002, Yaghi and co-workers designed and synthesized a series of Zn-based MOF-5 analogues, functionalized with −Br, −NH_2_, −OC_3_H_7_, −OC_5_H_11_, −C_2_H_4_, or −C_4_H_4_. These isoreticular MOFs featured different pore sizes and capacities for methane storage.^213^ The combination of terephthalic acid and Zr_6_ cluster can result in a chemically stable MOF, UiO-66, which can be functionalized with −NH_2_, −OH, −COOH, −OCH_2_CH_3_, −F, and −COOH.^276^ It is worth noting that the substitutes are highly associated with the catalytic performance of MOFs, including efficiency and selectivity.^277^ In particular, the presence of −NH_2_ can shift the MOF’s photoabsorption edge to the visible light region and improve its photocatalytic activity.^278^

**Figure 14:**
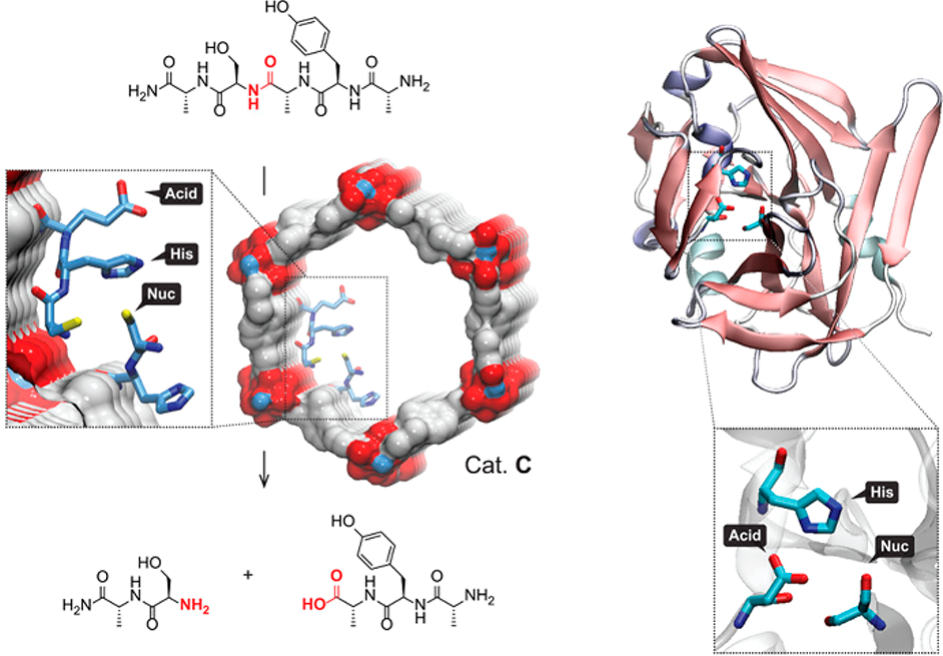
Pentapeptide cleavage using a multivariate MOF with enzyme-like structural complexity. Reproduced with permission from ref (282). Copyright 2016 American Chemical Society.

The postsynthetic method requires both the presence of modifiable sites and framework stability. Dynamic covalent chemistry is widely used for substituent modification to avoid framework collapse and loss of crystallinity.^279^ For instance, in 2009, Cohen and co-workers modified the −NH_2_ group of IRMOF-3 through condensation reactions to prepare over 10 multifunctional MOFs, fully uncovering the utility of postsynthetic modifications for divergent synthesis.^280^ Remarkably, Canivet and co-workers reported the first example of MOFs functionalized with peptides, starting with MIL-101-NH_2_, In-MIL-68-NH_2_, and Zr-UiO-66-NH_2_, and the resultant MOFs enabled catalyzing asymmetric Aldol reactions.^281^ In 2016, Yaghi and co-workers conducted seven postsynthetic reactions in one single MOF and successfully introduced tripeptides into the MOF ligand, resembling the structural complexity of enzymes.^282^ The resultant MOF with enzyme-like complexity enables selective cleavage of pentapeptide (Figure 14).

Another effective approach to constructing catalytic MOFs is selecting functional groups as the ligand backbone. The backbone functionalization is mainly conducted through presynthetic ligand design, which determines the connectivity and chemical stability of ligands. For instance, some enzyme-mimicking fragments, such as porphyrinic and Salen units, have been intensively studied. These functional fragments bring versatility to MOFs, including catalysis, sensing, and biomedicine.^283,284^ Inspired by the structure of peroxidase, Zhou and co-workers have developed plenty of porphyrinic MOFs, most of which are based on tetratopic porphyrin MOFs.^64,65,285−295^ These porphyrinic MOFs benefit from the porphyrinic units to possess great activities in catalytic oxidation. Salen-based ligands are chiral ligands featuring similar catalytic properties to porphyrin, representing a unique class of enantioselective catalysts.^296,297^ Compared with the substituent modification, the backbone functionalization may change the topology of the integral MOF, leading to the discovery of some unprecedented structures, while it usually involves more complicated ligand synthesis.

### 3.1 Chymotrypsin-Inspired MOFs

Chymotrypsin is an important proteolytic enzyme secreted by the pancreas in the alimentary canal, which can efficiently decompose denatured proteins and polypeptides in the duodenum.^298^ Chymotrypsin has been widely used in the treatment of sprain, otitis media, rhinitis, sinusitis, pharyngitis, and lung abscess. It also features utility in surgery for surgical inflammation, trauma, hematoma, abscess, and tracheotomy. Chymotrypsin belongs to endopeptidase and enables selectively hydrolyzing peptide bonds to cut off peptide chains. In addition, chymotrypsin is also known to catalyze the cleavage of ester bonds to hydrolyze lipids. The structure of chymotrypsin has been well characterized, defined as a hydrogen bond donating serine protease.

**Figure 15:**
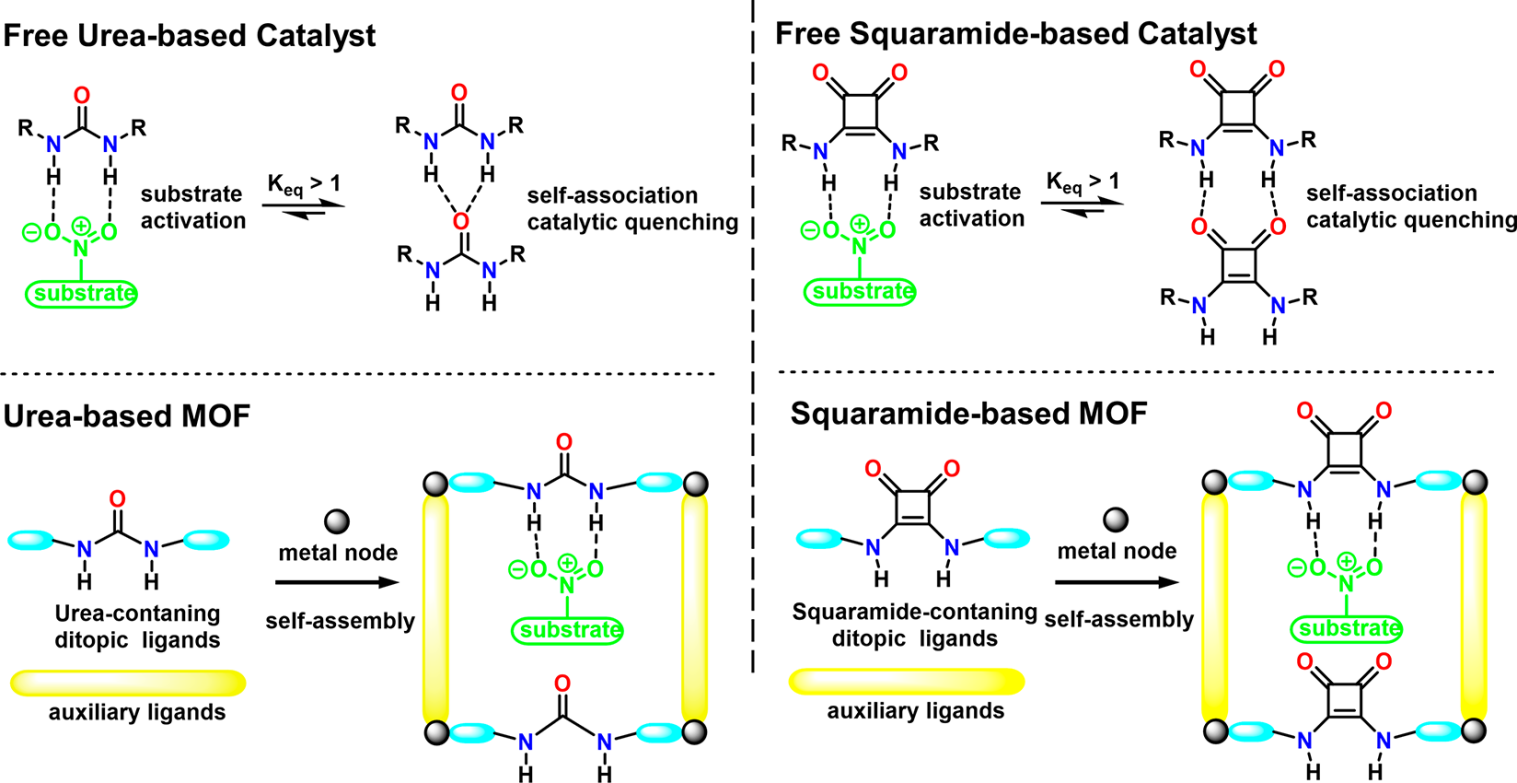
Long-range ordered arrangement of the urea/squaramide groups in frameworks helps to avoid the self-quenching of the active sites due to the oligomer formation for free urea/squaramide-based small molecules.

Inspired by chymotrypsin, two molecular catalysts, urea and squaramide, were developed with hydrogen bond donors.^299−301^ Yet, due to the competency of hydrogen-bond donors, the hydrogen-bond-donating catalysts can easily bond to each other through dimerization or oligomerization, significantly attenuating the solubility and reactivity of catalysts^60^ (Figure 15). Consequently, incorporating these catalysts into framework materials can provide confined environments and avoid this problem, leading to catalysts with enhanced stability and performance.^78,302^

#### 3.1.1 Urea-Based MOFs

**Figure 16:**
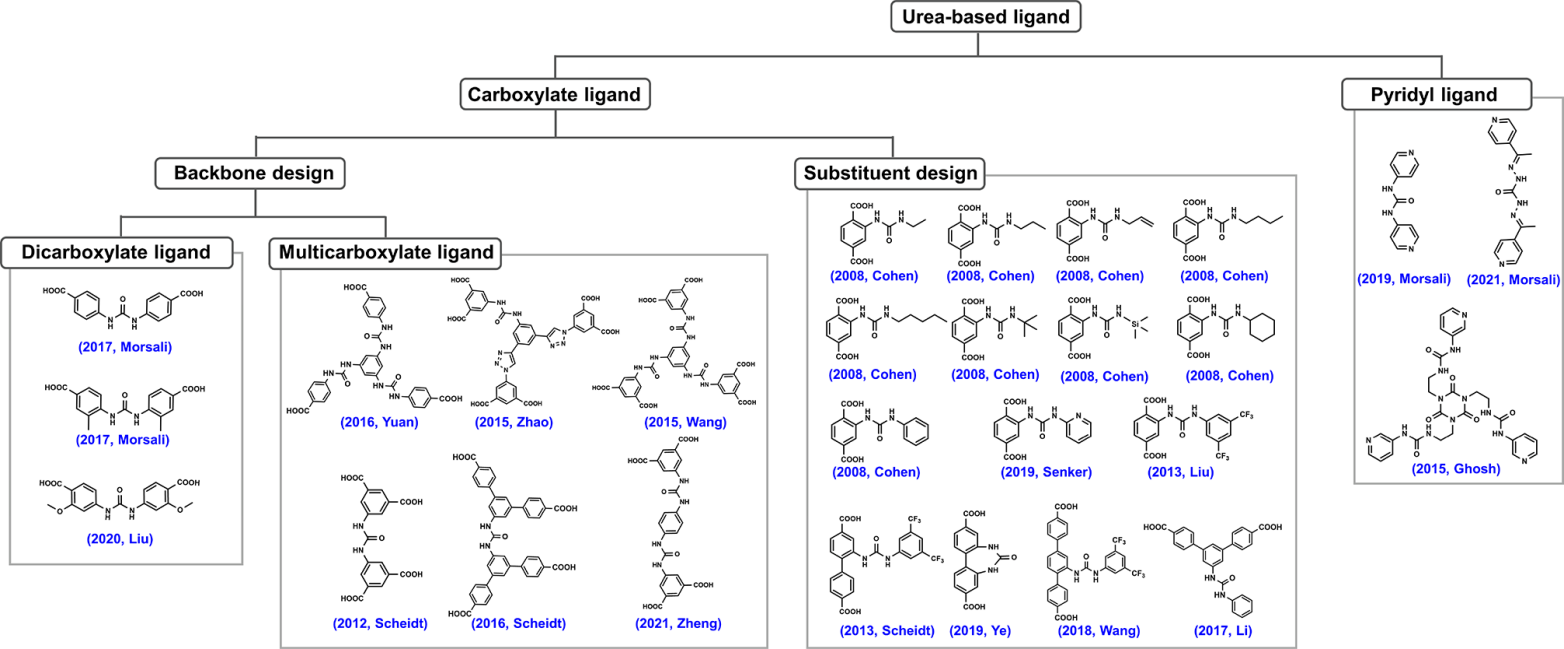
Structures and design principles for reported linkers used for constructing urea-based MOFs. The reported urea-based ligands consist of carboxylate groups or pyridyl groups. Urea groups can be incorporated into the backbone and substituent of the ligands, affording ligands with varied connectivities and configurations.

Due to the promising applications in anion recognition and separation, biomedicine, and catalysis, the urea group has been widely used to construct nonporous coordination polymers,^303−305^ coordination cages,^306^ crystalline capsules,^307^ supramolecular architectures,^308^ covalent organic frameworks,^309^ metallogels,^310^ as well as molecular organocatalysts.^311^ Combination of the multifunctionality of urea groups with the porous nature of MOFs may lead to novel catalysts with everlasting and selective performance. In the past ∼15 years, numerous MOFs containing urea groups have been reported. In 2008, Cohen and co-workers synthesized a series of urea-functionalized microporous MOFs using a postsynthetic modification method.^312^ A MOF named IRMOF-3 was selected as the prototype, consisting of NH_2_–BDC ligands and Zn_4_O clusters. Urea-functionalized MOFs were generated by condensing pendant amino groups on the ligand with various isocyanates (Figure 16). This work achieved a nearly quantitative conversion and the MOFs’ crystallinity was retained with slightly reduced BET surface areas. Similarly, the postsynthetic modification was also applied by Liu and co-workers using a highly stable MOF, MIL-101 containing BDC-NH_2_.^61^ The resultant MOFs served as heterogeneous catalysts exhibiting broad substrate scopes and excellent activity in Friedel–Crafts alkylation reactions. In addition to postsynthetically modification, urea-based MOFs can be synthesized directly using urea functionalized ligands. For example, Morsali and Liu groups developed dicarboxylate ligands with urea-based backbones to construct urea-based MOFs under solvothermal conditions.^313−316^ Multicarboxylate urea-based ligands with various sizes and geometries have been developed, significantly enhancing the structural diversity and functionality of urea-based MOFs.^317−320^ In addition, not only the number of the carboxylate groups can be tuned in the ligands, but more than one urea group can also be introduced into one single ligand, affording multiple hydrogen donors.^321−323^ Besides, pyridinyl-based ligands containing urea groups were also used to construct urea-based MOFs, as reported by Morsali and Ghosh groups.^324−326^ It should be noted that the dipyridinyl-based ligands usually result in two-dimensional layers or three-dimensional diamond-like networks.^324,327−331^ Some rare examples show pillared 2D MOFs based on urea-containing dipyridyl ligands and auxiliary dicarboxylate ligands.^332^ On the contrary, pillared-layered urea-based MOFs with dinuclear paddlewheel clusters can be easily obtained using urea-containing dicarboxylate ligands, pillared by linear dipyridyl ligands with various lengths.^333−337^ Interestingly, a pillared urea-based framework can also be formed using a tetra-carboxylate urea-based ligand and 4,4′-bipyridine.^60^ Apart from above examples, mixed-linker or mixed-metal MOFs containing urea groups have also been reported.^338−341^

Most of the urea-based MOFs are highly porous, exhibiting large BET surface areas, which can be used for adsorbing and separating multiple gases, including CO_2_, H_2_, CH_4_, C_2_H_6_, C_3_H_8_, SO_2_, and NH_3_.^323,335,336,339,341,342^ In a typical urea-based MOF, the urea groups in the ligands were well isolated from each other, which can be used as receptors for anions, such as F^–^, H_2_PO_4_^–^, Cl^–^, H_2_AsO_4_^–^, NO_2_^–^, HPO_4_^2–^, NO_3_^–^, HAsO_4_^2–^, SO_4_^2–^, and ClO_4_^–^.^332^ In addition, urea-based MOFs also demonstrate the capability to remove heavy metal cations, such as Hg^2+^ and Pb^2+^ in water.^324,329,331^ Besides, the accessible hydrogen bond donors endow urea-based MOFs with high selectivity and efficiency in detecting molecular species, including antibiotics, explosives, fluorescent dyes, and metal ions.^316,317,321,327,328,334,337,340,343,344^ Urea-containing MOFs also feature proton conduction capacity, leading to durable high conductivity materials.^345^

The exposed active sites and the accessible voids in urea-based MOFs make them perfect candidates for biomimetic catalysis. The urea group can activate electrophilic moieties toward nucleophilic addition via cooperative hydrogen bonding, which has been confirmed to lower the lowest unoccupied molecular orbital (LUMO) of the electrophile, such as nitro, carbonyl, or ether compounds. As a result, the activation barrier for nucleophilic attack will be decreased. As shown in Figure 1, the introduction of the active sites into MOFs helps to avoid the self-aggregation of catalysts and improve the catalytic activities. Therefore, urea-based MOFs have been used to catalyze various bond-forming transformations, such as Diels–Alder reactions and Friedel–Crafts reactions.^66^

The catalytic performance of urea-based MOFs in Friedel–Crafts reactions between β-nitrostyrene and *N*-alkylated pyrrole or indole is first studied, as this reaction is one of the most widely used approaches to synthesizing tryptamine derivatives. In 2012, Hupp and co-workers reported that the urea-based NU-601 exhibited effectivity and size-selectivity for the nucleophilic addition between *N*-alkylated pyrrole and β-nitrostyrene under 60 °C in the solvent of THF/MeNO_2_ (v:v = 1:1).^60^ The functions of the pores were also revealed by the evidence that large substrates showed significantly diminished yields versus small substrates. Similarly, a postsynthetic modified MIL-101 containing urea groups showed excellent catalytic activity and broad substrate scopes for the Friedel–Crafts reactions between β-nitrostyrene and *N*-alkylated pyrrole or indole.^61^ The large pore sizes of MIL-101 can facilitate the mass transfer of substrates, resulting in a heterogeneous catalyst with broad substrate scopes. In 2016, Yuan and co-workers developed a de novo approach to constructing MOFs with polydentate ligands but with different topologies.^322^ Four urea-containing MOFs with predesigned pore environments and catalytic sites were attained as reusable hydrogen-bond-donating catalysts, indicating varied catalytic capacities and size selectivity toward Friedel–Crafts reactions.

**Figure 17:**
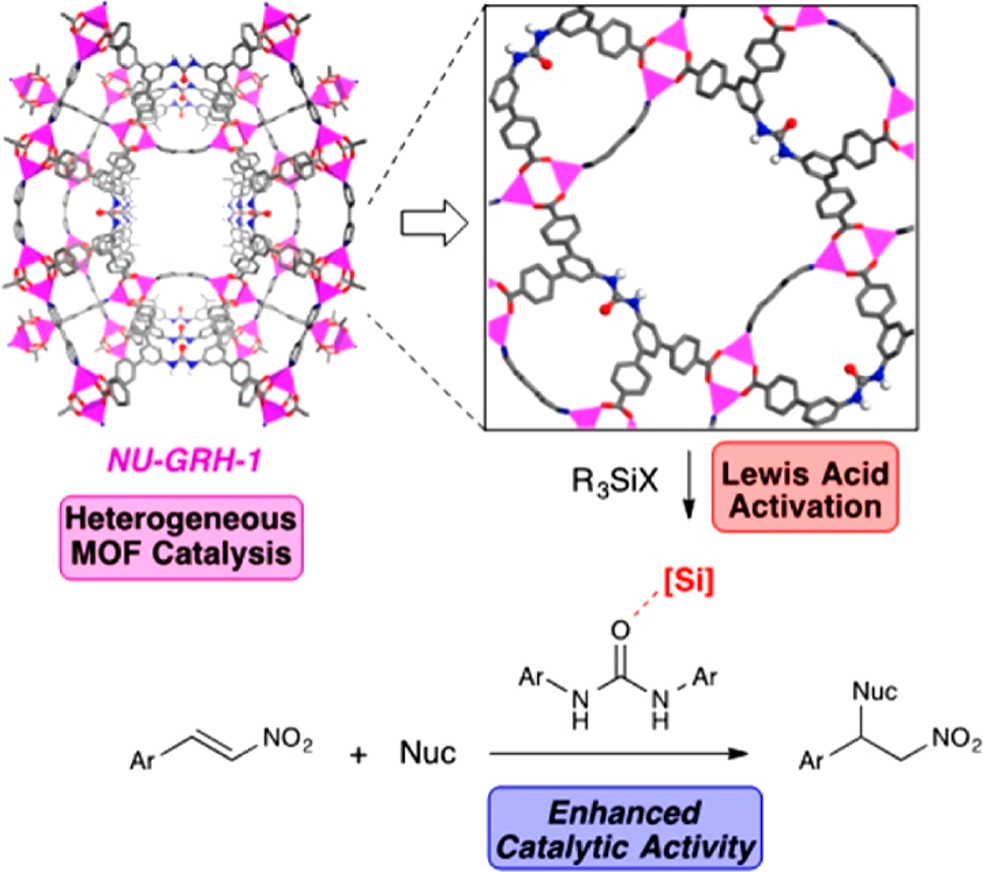
Enhanced catalytic activity of urea-based MOFs with the addition of Lewis acid. Reproduced with permission from ref (318). Copyright 2016 American Chemical Society.

Due to the remarkably lower nucleophilicity of pyrrole and indole, the Friedel–Crafts reactions between unsubstituted pyrrole or indole and β-nitrostyrene are slower than that of *N*-alkylated pyrrole or indole. To overcome this challenge, Scheidt and co-workers reported that a urea-based NU-GRH-1 featured excellent activity for the Friedel–Crafts reactions between unsubstituted indole and β-nitrostyrene with the presence of an activator trimethylsilyl chloride (TMS-Cl), in which the yield was improved from ∼19% to ∼98% (Figure 17).^318^

**Figure 18:**
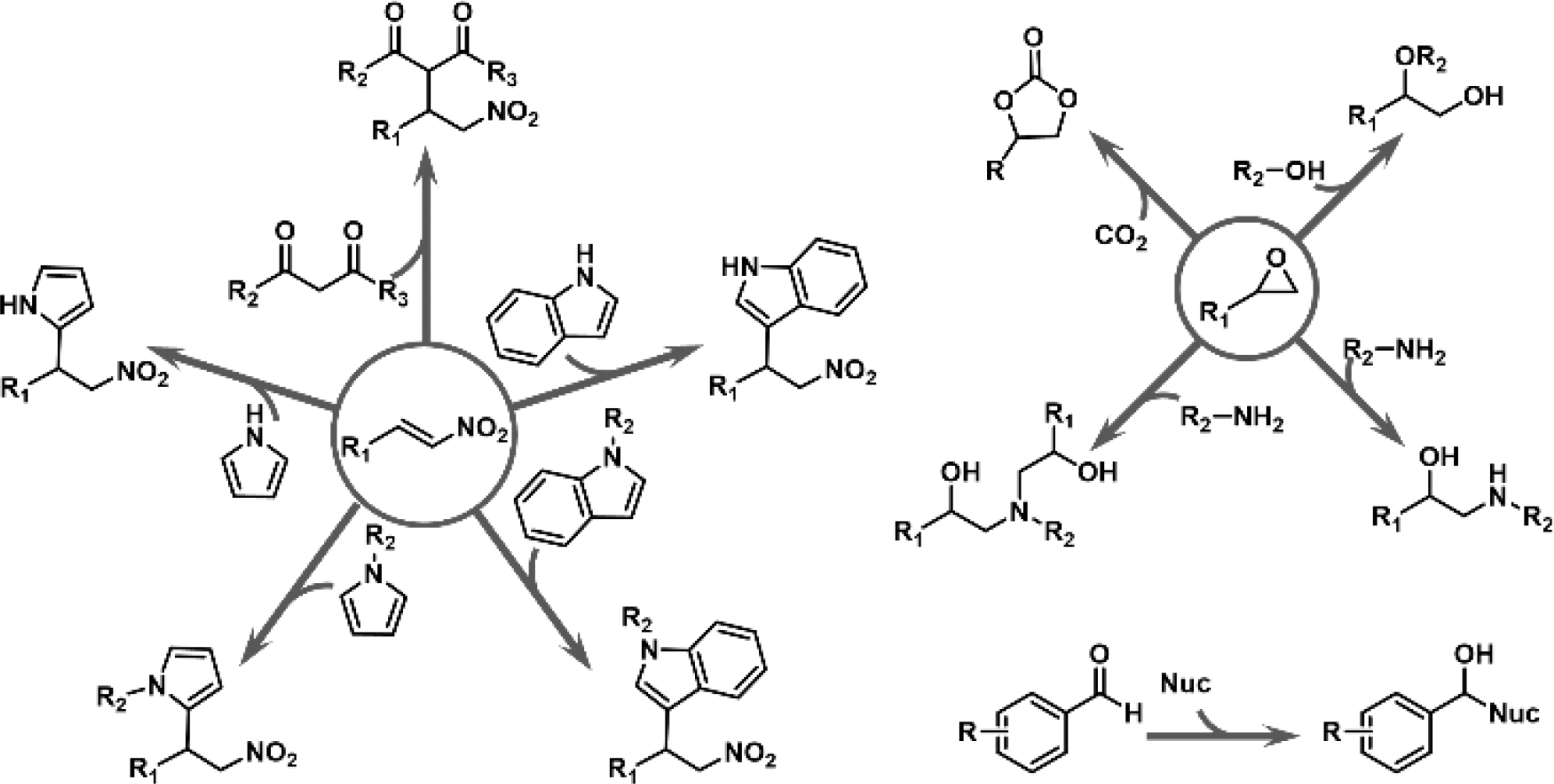
Summary of nucleophilic substitution reactions catalyzed by urea- and/or squaramide-based MOFs.

Besides pyrrole with different substituents, 1,3-dicarbonyls has also been selected as the nucleophile. Wang and co-workers demonstrated that the yield of the Michael addition reaction between 1,3-dicarbonyls and β-nitrostyrene reached up to ∼99% when a squaramide-based Zn-DBDA served as the catalyst under room temperature in water.^346^ Other electrophiles instead of β-nitrostyrene have also been investigated. Urea-based TMU-18 and -19 can achieve highly efficient methanolysis of epoxides, with methanol serving as nucleophile and solvent under 60 °C.^333^ Furthermore, in 2019, Ye and co-workers designed a urea-based MOF constructed with a lactam-derived ligand, which catalyzed cycloaddition between carbon dioxide and epoxide to produce cyclic carbonate with a ∼98% yield and 136 h^–1^ TOF under 1 atm and room temperature.^347^ Additionally, the reaction between electrophile aryl formaldehyde and different nucleophiles, such as nitro compounds and 1,3-dicarbonyls, has also been achieved using urea-based MOFs catalysts with high efficiency^348,349^ (Figure 18).

#### 3.1.2 Squaramide-Based MOFs

**Figure 19:**
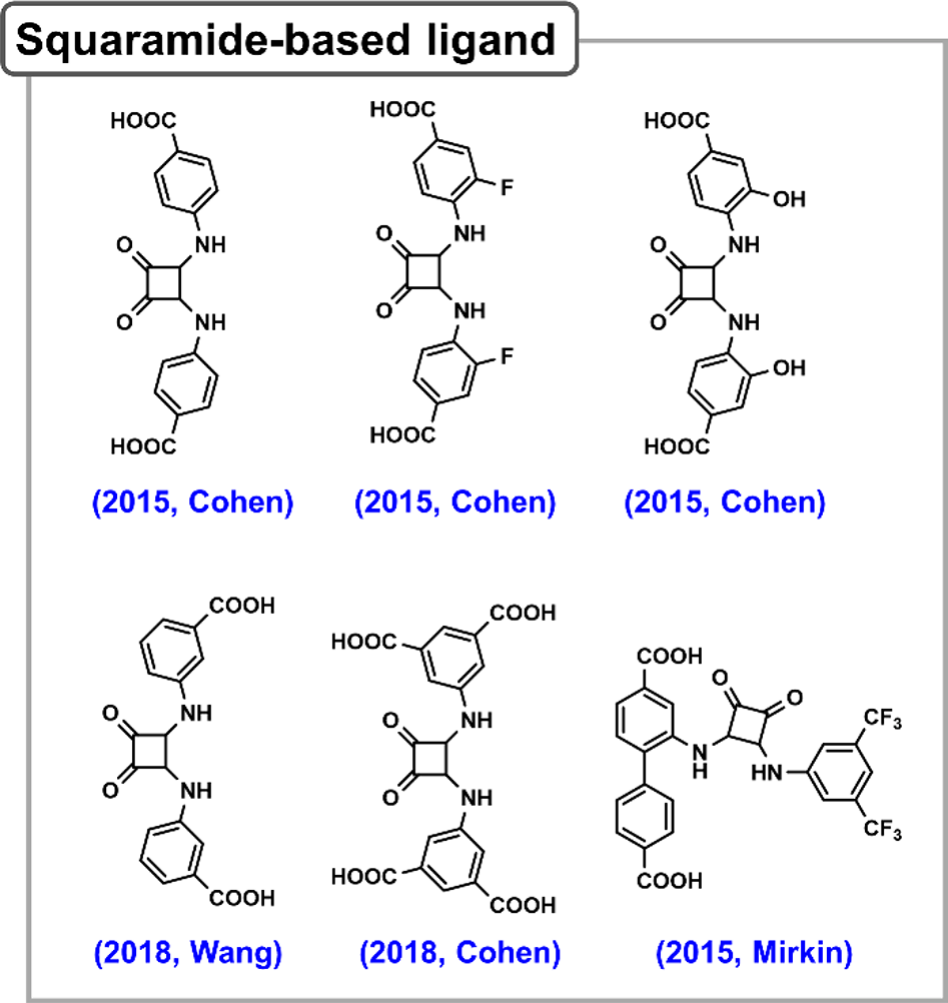
Summary of reported linkers used for constructing squaramide-based MOFs.

The squaramide skeleton has an aromatic quaternary cyclic rigid structure. As a double-hydrogen-bond acceptor/donor, the squaramide can selectively bind many guest species. Squaramide-based molecules have been widely investigated in supramolecular chemistry, catalytic chemistry, and chemical biology.^350^ These molecules show promise in anion binding and transmembrane transport, metal ions sensing, and chiral catalysis.^351−355^ Squaramide groups have been widely reported in diverse materials, including metallogels, coordination polymers, porous polymers, and COFs, enabling CO_2_ adsorption and cooperative conversion, NH_3_/NO sensing, as well as biomimetic organocatalysis.^356−359^ The self-quenching of the active sites due to the formation of oligomer for free squaramide-based small molecules can also be avoided by introducing squaramide groups into porous MOFs (Figure 15).^360,361^ Compared with the urea-based MOFs, studies on squaramide-based MOFs are still limited. To the best of our knowledge, only six ligands containing squaramide groups have been reported in MOFs, most of which are dicarboxylate ligands with varying lengths and geometries (Figure 19). Similar to the urea-based MOFs, squaramide-based MOFs feature high selectivity and efficiency in sensing bioactive molecules, such as histidine and lactose.^362,363^

**Figure 20:**
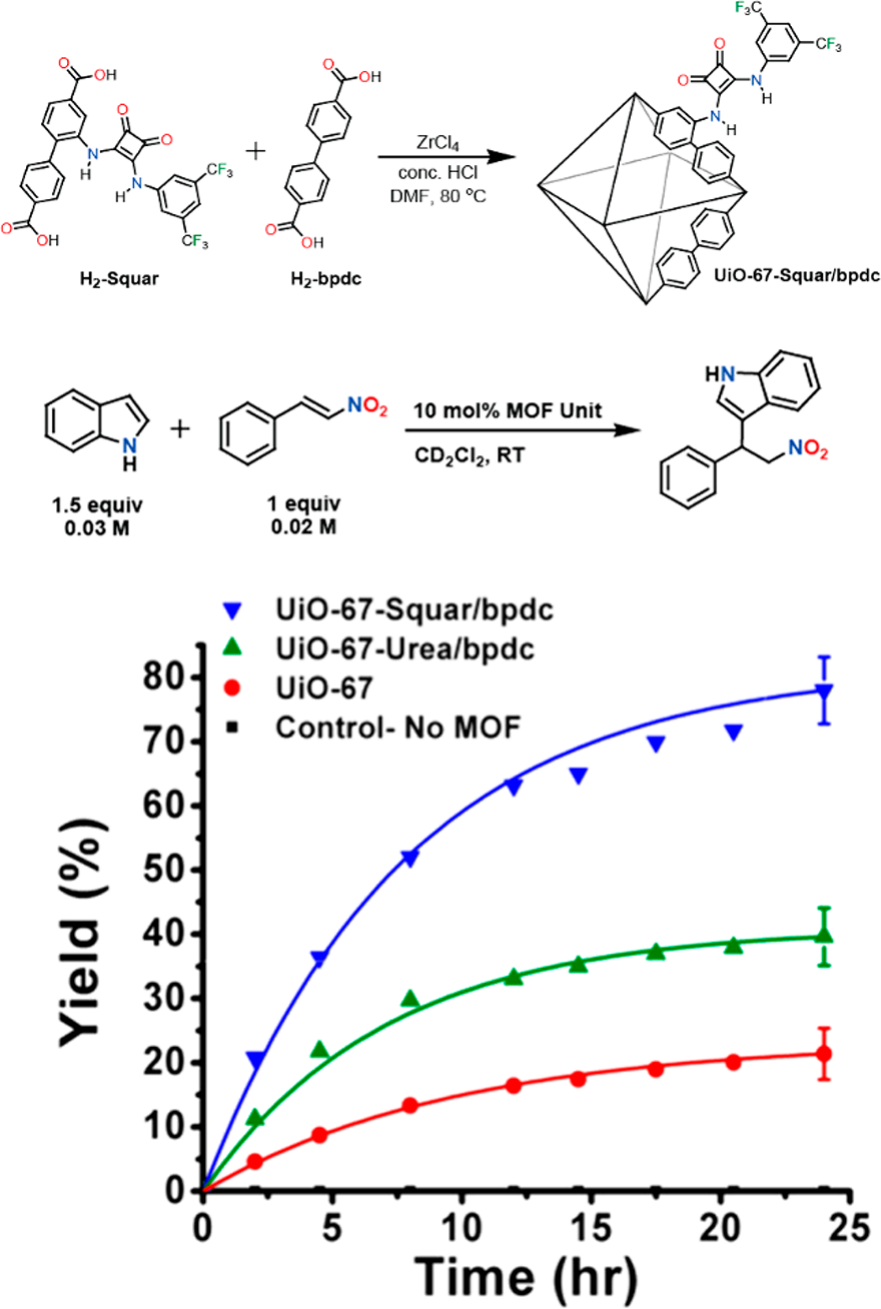
Synthesis of multivariate squaramide-based UiO-67 and its catalytic activity toward Friedel–Crafts reaction. Reproduced with permission from ref (66). Copyright 2015 American Chemical Society.

It has been confirmed that squaramide-based organocatalysts feature higher catalytic activity than urea-based ones, attributed to the enhanced electron density of the partially aromatic squaramide ring for resonance stabilization.^364^ Therefore, Mirkin and co-workers reported a linear ligand containing squaramide group as the side substituent, which can be utilized to construct a mixed-linker UiO-67-type MOF (Figure 20).^66^ The yield of the reaction between unsubstituted pyrrole and β-nitrostyrene can also reach up ∼51% under room temperature in the solvent of DCM, which is much higher than that of the urea-based MOF UiO-67-Urea/bpdc (∼28% yield). It is worth noting that free squaramide ligands could not catalyze the Friedel–Crafts reaction between unsubstituted pyrrole or indole and β-nitrostyrene, while assembling the squaramide motif into a framework would turn on their catalytic activity. Cohen and co-workers capitalized on the squaramide group as the backbone of dicarboxylate linkers, affording UiO-68-type MOFs and IRMOFs.^365−367^ In 2018, Wang and co-workers designed a bent squaramide-containing ligand, which can be used to synthesize MOFs with a 1D rhombus channel.^346,363^ In 2016, Cohen and co-workers utilized a squaramide-based tetra-carboxylate ligand to prepare a series of Cu-based MOFs through a postsynthetic exchange. These Cu-based MOFs feature high stability and catalytic performance as catalysts for the Friedel–Crafts reaction of indole and β-nitrostyrenes.^362,368^ Recently, Maspoch and co-workers designed four heterogeneous MOF-based catalysts with the linker 4,4′-((3,4-dioxocyclobut-1-ene-1,2-diyl) bis(azanedyil)) dibenzoic acid, which is identical in directionality and length to *p*,*p*′-terphenyldicarboxylic acid, acting as efficient catalysts in Friedel–Crafts alkylation and epoxide ring-opening reactions.^366^ When amino compounds were selected as a nucleophile, solvent-free epoxide ring-opening reactions with controlled monoaddition or tandem reactions can be conducted in the pores of squaramide-based MOFs, yielding 1,2-aminoalcohols or 1,2,2′-aminodialcohols, respectively.^366,367^ (Figure 18)

In summary, chymotrypsin mimicking MOFs benefit from the framework structures to fix the double hydrogen bonding units on target positions, eliminating the self-association effect that often occurs in molecular catalysts. Therefore, chymotrypsin-mimicking MOFs feature superior catalytic activity compared with molecular catalysts, and the porous nature endows MOFs with size-dependent selectivity in catalysis.

### 3.2 Phosphotriesterase-Inspired MOFs

**Figure 21:**
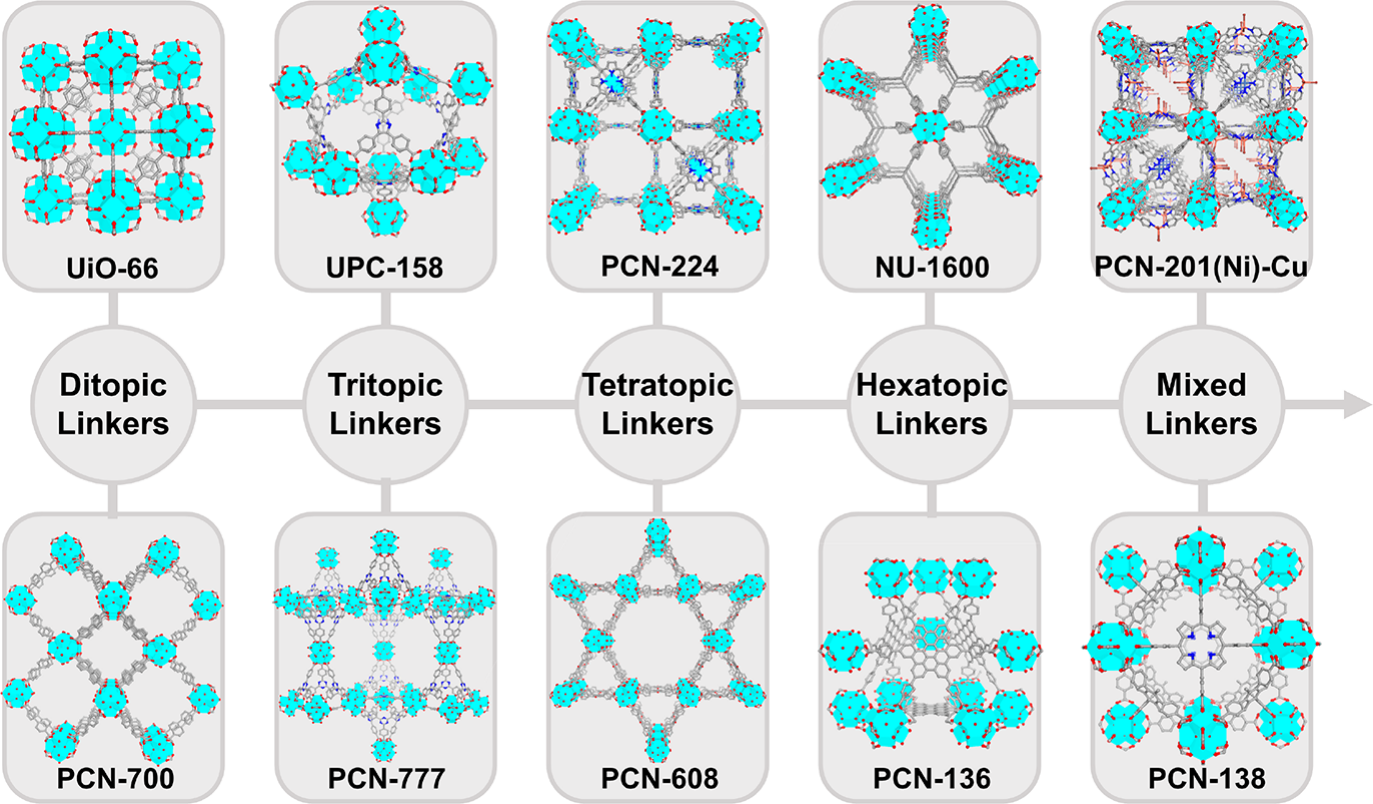
Representative MOFs based on Zr_6_ clusters and organic linkers with varied connectivities, including ditopic, tritopic, tetratopic, hexatopic linkers, and their mixtures.

Organophosphorus or organophosphate compounds with only a weak odor are known as nerve agents, which are highly toxic and lethal chemicals (Figure 21). These agents have a strong inhibitory effect on the acetylcholinesterase in the brain, diaphragm, and blood. As a result, the excessive accumulation of acetylcholine in the body will induce severe functional disorders of the central and peripheral cholinergic nervous system.^369−374^ Owing to their stability and easy production, nerve agents have become the primary chemical warfare agent (CWA). The continuous threat of terrorist attacks using CWAs has prompted research on new materials capable of removing CWAs at ambient temperature and humidity. Chemicals containing phosphonate linkages, such as DMNP, VX, GB (Sarin), and GD (Soman), are extremely toxic CWAs, and their detoxification is urgently demanded.

**Figure 22:**
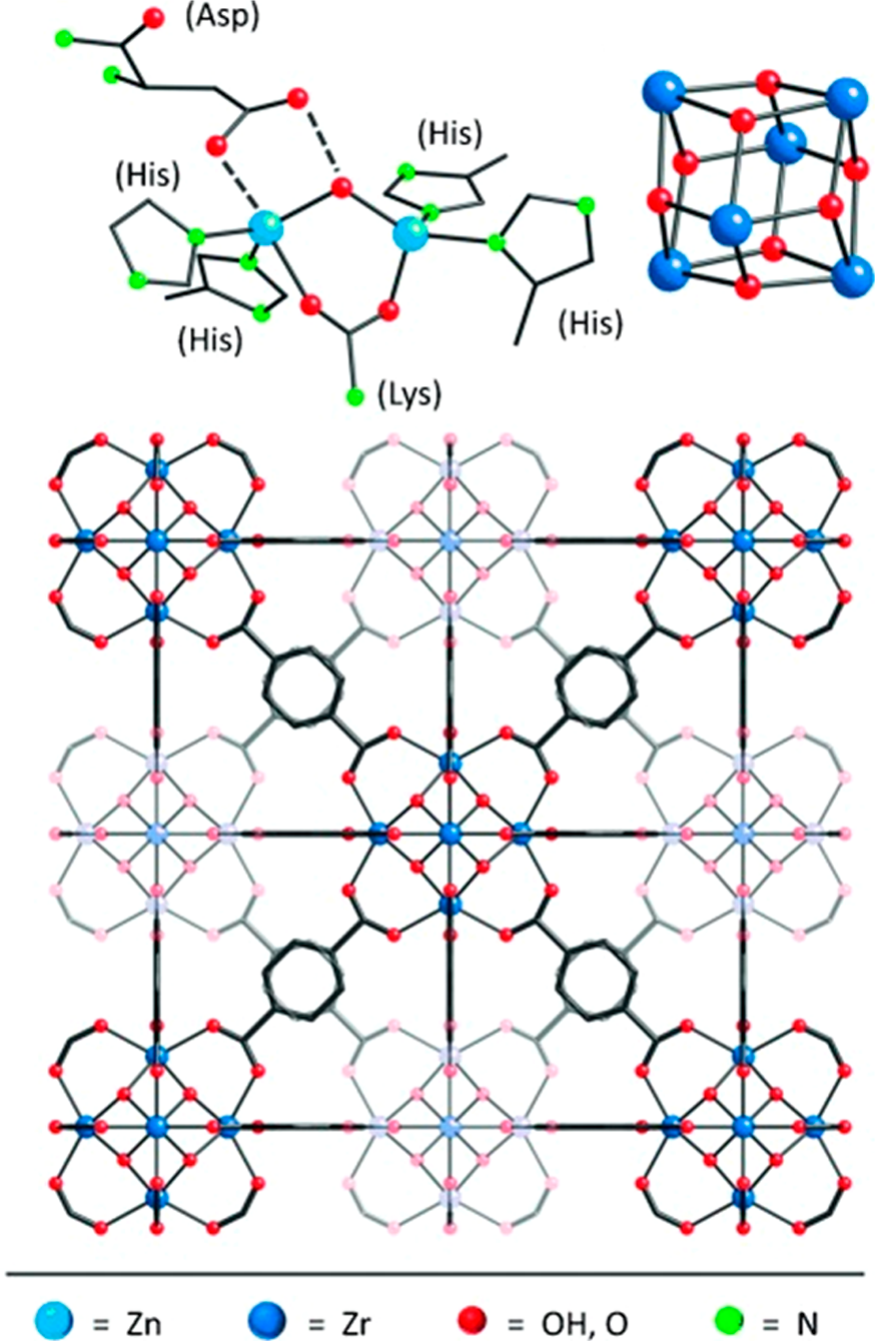
Structural illustrations of phosphotriesterase’s active site and a Zr_6_-based MOF named UiO-66. Reproduced with permission from ref (63). Copyright 2014 John Wiley and Sons.

Widely discovered in *Pseudomonas diminuta*, flavobacterium, and other biosystems, phosphotriesterase enables the hydrolyzation of phosphate ester, which is widely used as phosphorus-containing pesticide or nerve agent.^375−378^ It is reported that the bridged hydroxyl ligands in phosphotriesterase play important roles in the hydrolysis of phosphate ester, which is highly useful for the detoxification of certain nerve agents to save lives. Two zinc(II) metal ions are bridged by one hydroxyl ligand, one of which binds to the oxygen atom of P=O to activate the phosphate ester, and the other transfers the hydroxyl to cleave the ester group of the substrate (Figure 22).^63^ Inspired by phosphotriesterase, various MOFs with bridged hydroxyl groups, especially Zr_6_-based MOFs, were developed as catalysts for the hydrolysis of nerve agent simulators.

#### 3.2.1 Zr_6_-Based MOFs

In 2008, Lillerud and co-workers reported three isoreticular microporous MOFs based on Zr_6_ clusters, termed UiO-66, -67, and -68.^379^ Since then, zirconium-based MOFs have attracted more and more attention due to their ultrahigh stability, diverse structures, and versatile properties. A few relevant reviews have comprehensively summarized the synthesis, structures, and properties of zirconium-based MOFs.^72−75^ Zr_6_-based MOFs features a collection of bridged μ_3_–OH groups, similar to the phosphotriesterase active site, serving as the potential site to catalyze the hydrolysis of phosphate esters. Therefore, some representative examples are briefly described here to introduce the structural design and catalytic activity of Zr_6_-based MOFs.

UiO-type MOFs are the most well-known Zr_6_-based MOFs, in which 12 ditopic linker carboxylate groups are coordinated with the saturated Zr_6_ clusters.^379,380^ In contrast, the connection number of the Zr_6_ clusters will reduce to 8 in the resultant PCN-700, with two uncoordinated methyl groups introduced to the ditopic linker, which will slightly change the symmetry of the linker.^44,381^ The connection number of the Zr_6_ clusters will be further reduced to 6 when a tritopic linker BTC is applied. MOF-808 and PCN-777 are two examples of such systems with spn topology.^382,383^ Tetratopic linkers have been extensively studied in Zr_6_-based MOFs, leading to the discovery of diverse MOFs with structural complexity and robust nature. For example, using a porphyrin-containing tetracarboxylate ligand, tetrakis(4-carboxyphenyl)-porphyrin (TCPP), at least five kinds of Zr_6_-based MOFs, namely PCN-221/MOF-525, PCN-222/MOF-545, PCN-223, PCN-224, and PCN-225, all with different topologies, can be constructed.^384−386^ NU-901 and NU-1000 with pyrene-based linker; PCN-605, PCN-606, and PCN-608 with biphenyl-based linker are also examples of such topological polymorphism.^387,388^ Hexatopic linkers with different sizes and symmetries have also been used to construct Zr_6_-based MOFs, such as NU-1600 and PCN-136.^389,390^ Mixed linker Zr_6_-based MOFs can also be obtained by one-pot syntheses, such as PCN-134 with tritopic BTB and tetratopic TCPP linkers, and PCN-138 with tritopic TBTB and tetratopic TCPP linkers.^287,389,391,392^ For Zr_6_-based MOFs bearing 6- or 8-connectivity Zr_6_ clusters, linear ditopic linkers can be introduced through postsynthetic linker installation to achieve mix-linker MOF.^84,85,393−396^ In general, the high stability, structure diversity, and surface areas, together with the bridged hydroxyl groups on zirconium clusters, make Zr_6_-based MOFs promising candidates for the hydrolysis of nerve agent simulators.

Inspired by the natural enzyme phosphotriesterase, in 2014, Hupp and co-workers tested the hydrolysis properties of UiO-66 on a phosphate-based nerve agent simulant DMNP. The combination of the strong Lewis acidic Zr^IV^ cations and bridging hydroxides led to ultrafast reaction rates for a hydrolysis reaction. With only surface-only catalysis observed, lowering the actual catalyst loading to merely 0.045%, the result is remarkable (Figure 21).^63^ Since then, intense efforts have been devoted to improving the catalytic efficiency and selectivity of MOFs on the hydrolysis of phosphate-based nerve agent simulants.

**Figure 23:**
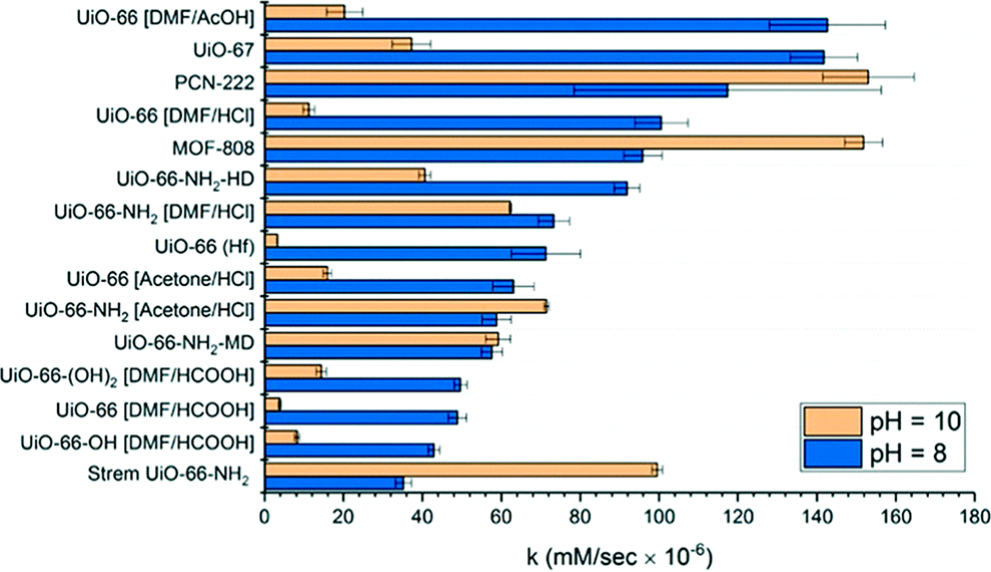
High-throughput screening of Zr_6_-based MOFs for DMNP degradation, in which the catalytic activity at pH 8 and 10 were compared. Reproduced with permission from ref (410). Copyright 2018 Royal Society of Chemistry.

Zr_6_-MOFs with different structures and functional groups have been examined.^397−401^ Effects of the MOF topologies, defects, particle sizes, pH values of the reaction systems, and different amine-based bases have been systematically studied.^402−406^ In 2015, Hupp and Farha reported a highly porous and stable MOF NU-1000 that was extraordinarily effective for degrading nerve agents and their simulants. NU-1000 overcame many challenges that traditional materials often meet, such as the low sorptive capacities, low active site loadings, hard deactivation of the active site, slow degradation kinetics, and limited structural tunability. Herein, NU-1000 is highly active in the destruction of the nerve agent simulant dimethyl 4-nitrophenyl phosphate and the highly toxic CWA Soman. Computational results suggest that the extraordinary activity of NU-1000 is engendered by the unsaturated Zr_6_-cluster and weak intermolecular interactions, which direct orientations between the substrate and catalyst. The mesoporous channels in NU-1000 allow substrates access to active sites.^407^ In addition, Hupp, Farha, and co-workers reported that UiO-67-NH_2_ was more efficient in hydrolysis than UiO-67 and UiO-67-NMe_2_ because the amino moiety served as a proton-transfer agent during the catalytic cycle.^408^ Frenkel and co-workers presented a comprehensive study on the influence of carbon dioxide on the capture and decomposition of DMMP by MOF-808, so as to improve the catalytic performance of the material under battlefield conditions.^409^ They found that the presence of CO_2_ in the pores of MOF-808 hindered the decomposition of DMMP due to the preferential formation of carbonate on the bridged hydroxyl ligand. In 2018, Cohen and co-workers utilized a high-throughput screening method to accelerate the discovery and evaluation of nerve agent degradation catalysts.^410^ As a result, none of the zeolites or metal oxides show comparable activity with MOFs, especially the UiO-66 series, which shows the unparalleled superiorities of MOFs toward triester decomposition (Figure 23).

**Figure 24:**
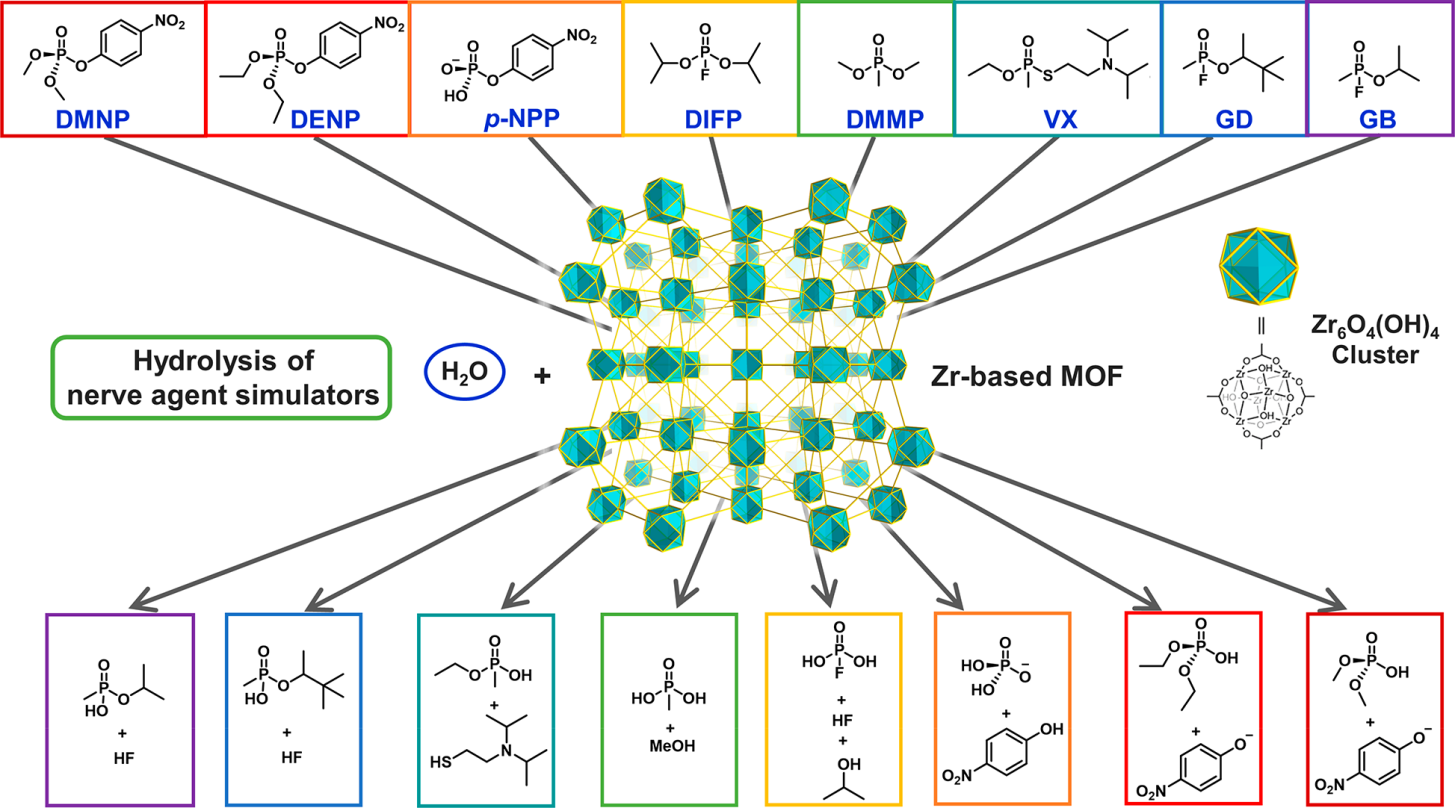
Hydrolysis of nerve agent simulators containing phosphonate linkages, which is catalyzed by phosphotriesterase inspired MOFs with Zr_6_O_4_ (OH_4_) clusters.

In order to enhance the catalytic properties of Zr_6_-based MOFs, some auxiliary species or cocatalysts have been introduced into the MOFs, such as amine, Fe_2_O_3_, polydopamine, linear-polyethylenimine cationic polymer, imidazolate, and polyoxometalates.^411−416^ The obtained composite materials show high efficiency in hydrolyzing a broad range of nerve agent simulators, including DMNP, DMMP, DIFP, VX, GD, and GB (Figure 24).^414−416^ In addition, the hydrolysis mechanism based on the MOF catalysts has also been studied in depth using in situ synchrotron-based X-ray powder diffraction, X-ray absorption, infrared spectroscopy, and phosphorus-31 solid-state-magic-angle spinning nuclear magnetic resonance (^31^P SS-MAS NMR), revealing essential aspects of the reaction mechanism.^387,417^ As expected, the substrates were adsorbed into pores of MOFs first, and then coordinated to the Zr_6_ cores directly, which would be decomposed into phosphonate as final products.

**Figure 25:**
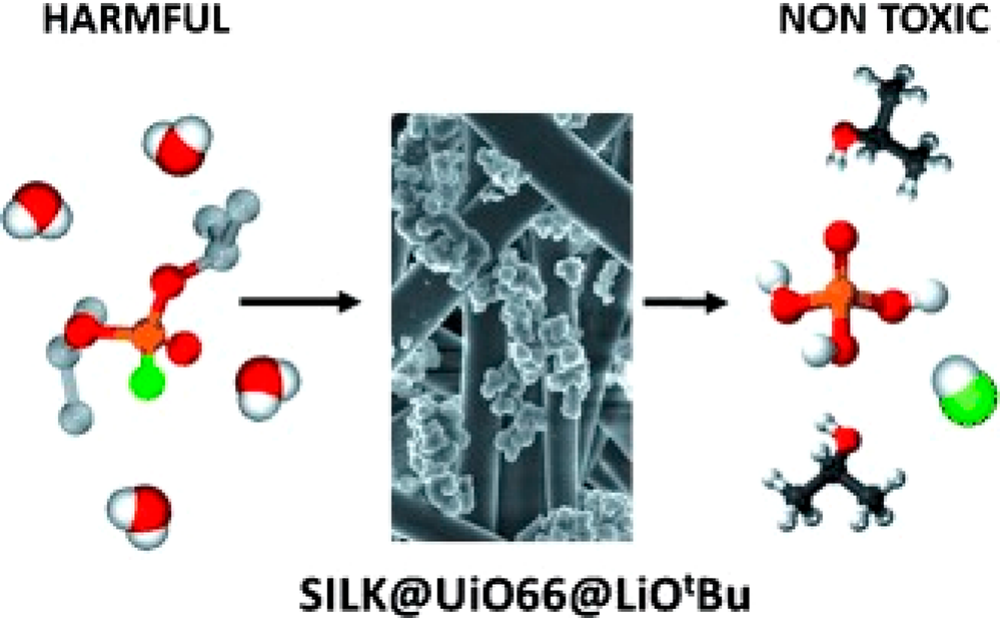
Fabrication of SILK@UiO-66@LiO^t^Bu for CWA degradation. Reproduced with permission from ref (424). Copyright 2015 John Wiley and Sons.

The fabrication of phosphotriesterase-inspired MOFs is also critical for practical application under working conditions. Therefore, many efforts have been made to fabricate the MOFs into mixed-matrix membranes, nanofiber kebabs, hydrogels, or integrate the MOFs onto activated carbon, fibers, and polymer sponge.^418−423^ In 2015, Barea and Navarro took advantage of lithium alkoxide doped UiO-66 to develop self-detoxifying adsorbents of CWA containing hydrolyzable P–F, P–O, and C–Cl bonds.^424^ This work demonstrated a novel strategy to combine air-permeation properties of the textiles with the self-detoxifying properties of MOFs, paving the way to integrate MOFs into self-detoxifying protective fabrics (Figure 25). In 2018, Peterson and Epps described a new strategy for fabricating mixed matrix composites containing layered MOF/polymer films for CWA protection.^425^ Incorporating MOFs into the core layer led to efficient removal of CWA while simultaneously promoting moisture vapor transport through the composite, showcasing the promise of these composites for protection applications. In 2019, Farha and co-workers developed a composite material MOF-808/linear polyethylenimine/fiber, which showed high catalytic activity for the hydrolysis of a nerve agent under ambient conditions.^426^ Notably, this composite showed enhanced activity in bulk water for the hydrolysis of DMNP and Soman compared to previously reported MOFs.

#### 3.2.2 MOFs with Other Metals

Many coordination complexes have been reported for the catalytic degradation of organophosphates to imitate the active sites in phosphotriesterase, especially the complexes based on Zn^2+^ ions and Ln^3+^ ions.^427−433^ These studies have provided valuable design principles for the construction of MOF-based mimics. Herein, a few MOFs containing other metal nodes have also been applied to the catalytic hydrolysis of nerve agent simulators recently, including Ce, Zn, Fe, and Ti.^434−437^ For example, Navarro and co-workers studied the DIFP detoxification properties of a series of Ce/Zr-mixed MOFs.^438^ The catalytic rate for the P–F bond cleavage was improved with increasing Ce/Zr molar ratio, and a further enhanced catalytic efficiency can be achieved by doping Mg(OMe)_2_ into the pores of Ce/Zr-MOFs. In addition, Zhang and co-workers reported a Zn-based MOF adopting the active center of organophosphorus hydrolase, which was assembled via rational combination of functional ligands, resulting in efficient decomposition of DENP without bridged hydroxyl ligand and cocatalytic base.^439^ Yamada and co-workers reported that an Fe/Co-based Prussian-blue-type MOF can also be used for organophosphate hydrolysis, and the catalytic activity and stability of *p*-NPP hydrolysis can be improved by doping of divalent metal ions, such as Mn^2+^, Ni^2+^, or Cu^2+^.^437^ Interestingly, multiple functions can be achieved through the introduction of other functional units, such as single-component degradation and detection of VX by chromophore functionalization, and tandem catalysis by integrating the merits of artificial enzyme and metal nanoparticle catalyst.^440,441^

In summary, a series of Zr_6_-based MOFs have been proved as excellent catalysts for hydrolyzing organophosphate triesters, while studies on MOFs with other metal nodes have not been fully investigated, indicating a potential seeding point for developing MOFs to decompose CWA. Besides, fabricating MOF-based composite materials can improve their applicability in practical applications.

### 3.3 Dehydrogenase-Inspired MOFs

Dehydrogenases are enzymes belonging to oxidoreductases that oxidize a substrate by reducing an electron acceptor, usually NAD^+^/NADP^+^ or a flavin coenzyme such as FAD or FMN.^442,443^ They catalyze reverse and forward reactions with great physiological significance, like all catalysts. For example, alcohol dehydrogenase catalyzes ethanol oxidation to acetaldehyde in animals, and a reverse transformation can take place in yeast.^444^ This section will focus on two dehydrogenases, namely CO dehydrogenase (CODH) and formate dehydrogenase (FDH), which have great potential in CO_2_ fixation and reduction to address the challenge of global warming. In detail, the active center of these dehydrogenases, the development of MOFs to mimic them and perspectives of the future for designing dehydrogenase-inspired MOFs will be covered in this section.

CODH can be briefly divided into two types based on the active center: Mo-[2Fe-2S]-FAD in aerobic bacteria and Ni-[3Fe-4S]-CODH in anaerobic bacteria. Currently, most research focuses on Ni-CODH. Ni-CODHs can catalyze the reversible transformation between CO_2_ and CO at a rate of 15 756 U/mg at pH 8 and 70 °C.^445^ There are five widely accepted crystal structures of Ni-CODHs.^445−450^ All those structures contain five metal clusters, which are two nickel–iron–sulfur C-clusters, one Fe_4_S_4_ D-cluster, and two Fe_4_S_4_ B-clusters. The B-clusters feature a different morphology from the D-clusters. Of all the clusters, C-clusters are believed to be the active center of Ni-CODHs. Several critical features have been observed in the catalytic cycles.^451^ Molecular water is bound to the pendant Fe site of the C-cluster, associated through hydrogen bonding with Lys563, His93, and His263. This coordination sphere created by these residues is essential to the activity of CODH.^452^ When the whole system is under CO treatment, the Ni coordination geometry transforms closer to tetrahedral, with the average Ni–S distance increasing to 2.25 Å, suggesting a structural rearrangement in the C-cluster. However, no changes in the Ni oxidation state have been observed.^452^ After the Fe-bound hydroxide attacks the Ni-CO, the Ni coordination geometry further changes from tetrahedral to square planar in a CO_2_-bound form.^449^ The redox of the C-cluster releases CO_2_ and a proton, with two electrons transferred to the B- and D-clusters.^453^ The distance between the metal clusters is approximately 11 Å, making it a suitable electron transfer route.^454^ In conclusion, the pendant Fe site, Ni site, and the coordination sphere play a vital role in the catalytic cycle between CO_2_ and CO. Several molecules, including nitrous oxide, sulfide, azide, thiocyanate, cyanate, cyanide, and n-BIC,^443,455−458^ are known to inhibit the catalytic activity of CODHs because these molecules will compete with CO or CO_2_ to bind the metal site. Therefore, it is essential to remove these molecules when performing catalytic tests.

FDH has long been considered as an enzyme only catalyzing the irreversible transformation from formate to CO_2_.^459^ However, a recent study has found that certain FDHs could reversibly interconvert CO_2_ and formate, such as the case in CO_2_ reductase.^460^ Barlow made an in-depth discussion about the mechanism, and the widely accepted mechanistic proposals have concluded several factors.^461^ The Mo or W center is transformed between a +4 and +6 oxidation state, and the electrons and protons are not colocated on the metal. A proton is required to complete the hydride transferred from a ligand or the secondary coordination sphere.^462−466^ Creating more hydrogen bonds around the active center may enhance the performance of FDH.

There are also many reports focused on enzyme engineering to enhance the performance of FDH. For example, mutation of the active site has been promoted to loosen the restriction of the substrate positioning, elevate the conformational flexibility, and increase the accessibility of the active sites.^467^ Changing the positioning of the cofactor to facilitate hydride transfer from NADH to HCO_3_^–^, resulting in an improved catalytic turnover rate (*k*_cat_) and total catalytic efficiency (*k*_cat_/K_m_).^468^

The active site of the molybdenum-dependent (Mo-FDHs) and tungsten-dependent enzymes (W-FDHs) is Mo or W atom coordinated by two molybdopterin ligands.^461^ Based on this structure, it is promising to design and construct a structure in which Mo is coordinated with dithiolene ligands and a terminal sulfide to mimic the coordination sphere of FDH.

The rapid increase of CO_2_ concentrations has threatened the environment. It is urgent to find an efficient way to capture and convert CO_2_ into valuable products. MOFs, as promising candidates for CO_2_ utilization, have attracted great interest. Jiang and co-workers divided the development process of CO_2_ utilization into three parts,^275^ namely tuning the CO_2_ adsorption capacity and selectivity of MOFs, developing MOF-based materials for the conversion of CO_2_ to organic products, and expanding the reaction scope of possible CO_2_ conversion. Herein, we would like to offer a novel way to design MOFs for CO_2_ utilization, take a page from nature to emulate the structure of CODH and FDH.

**Figure 26:**
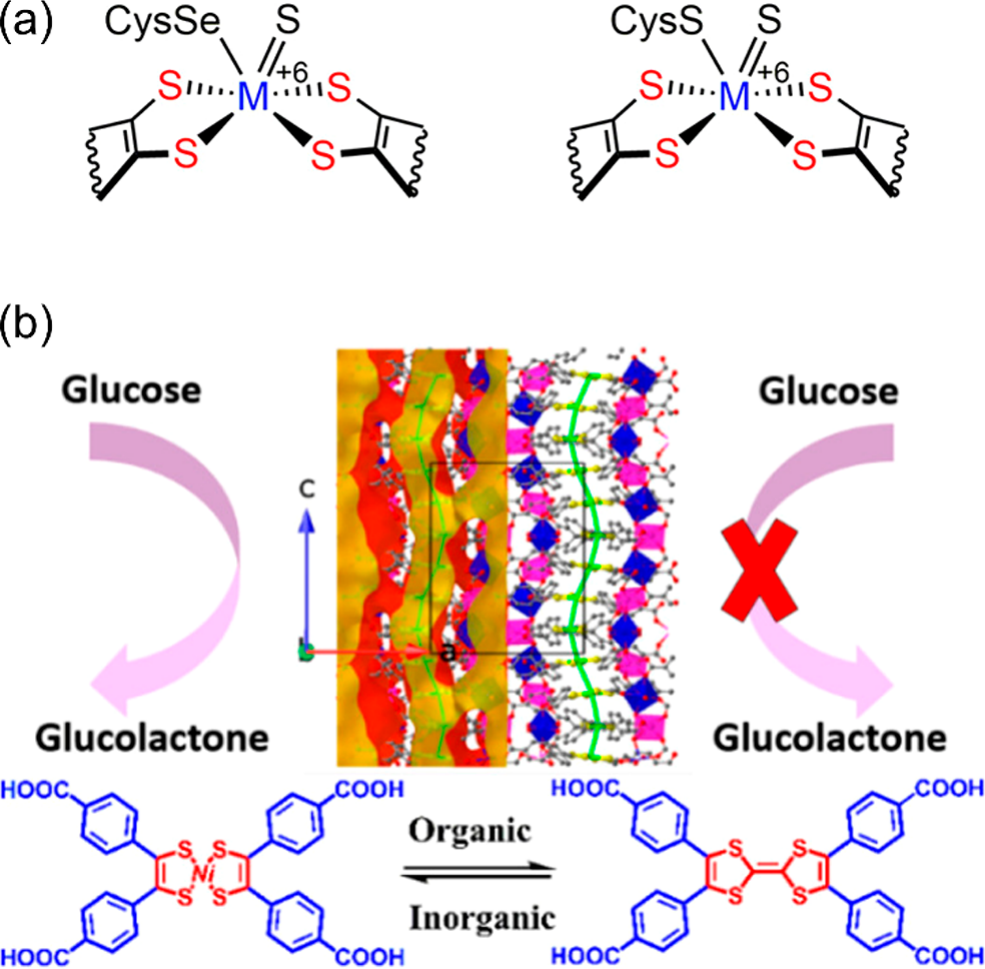
Ligand design of dehydrogenase-mimicking MOFs. (a) Active sites of formate dehydrogenase (FDH). (b) Crystal structure of a dehydrogenase-mimicking MOF as an electrochemical glucose sensor. Reproduced with permission from ref (218). Copyright 2020 American Chemical Society.

**Figure 27:**
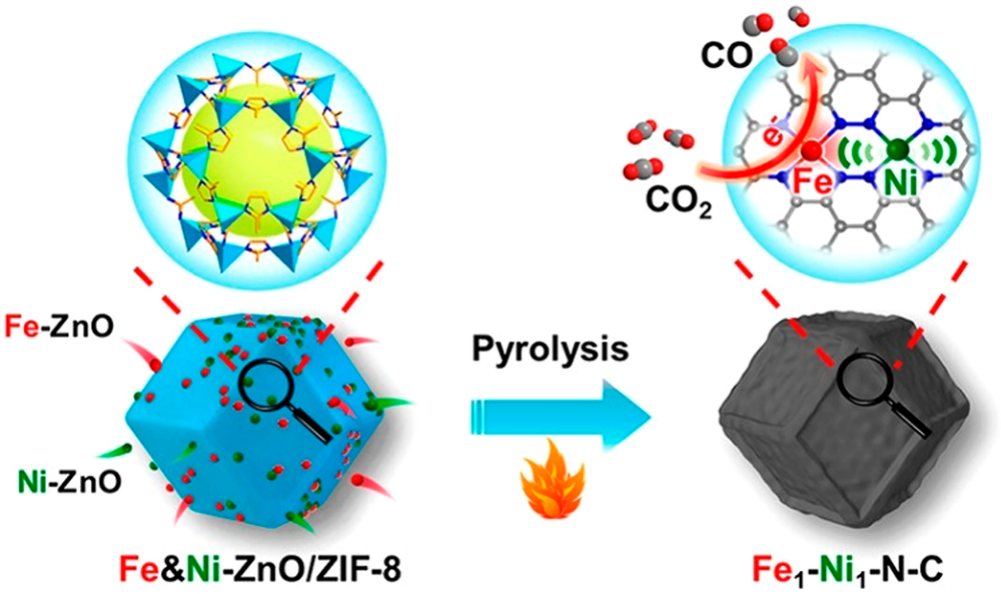
Preparation of MOF-derived carbon with neighboring Fe and Ni single-atoms, which was applied in CO_2_ electroreduction. Reproduced with permission from ref (470). Copyright 2021 American Chemical Society.

However, very few examples mimic the active center of dehydrogenase in MOFs.^141,469^ Zuo and co-workers reported the first example by introducing nickel bis(dithiolene-dibenzoic acid) into MOFs to attain [Mn_2_{Ni-(C_2_S_2_(C_6_H_4_COO)_2_)_2_}(H_2_O)_2_]·2DMF.^218^ The nickel bis(dithiolene-dibenzoic acid) resembles the active center of FDH while the metal center was changed. (Figure 26) The new MOF is an excellent electrochemical glucose sensor due to the multiple oxidation states of the [NiS_4_] core. It features a wide linear detection range from 2.0 × 10^–6^ to 2.0 × 10^–3^ M. As advancement in this work, Zuo and co-workers synthesized a MOF (Me_2_NH_2_^+^){In^III^-[Ni(C_2_S_2_ (C_6_H_4_COO)_2_)_2_]}·3DMF·1.5H_2_O using nickel bis(dithiolene-dibenzoic acid) and explored its potential in CO_2_ reduction to HCOO^–^.^217^ The MOF showed a higher conversion rate and Faradaic efficiency (FE) compared to the isomorphic MOF (Me_2_NH_2_^+^)[In^III^-(TTFTB)]·0.7C_2_H_5_OH·DMF, with FE_HCOO_^–^ increasing from 54.7% to 89.6%. Inspired by the structure of C-clusters in CODH, Jiang and co-workers pyrolyzed MOFs assembled with Fe- and Ni-doped ZnO nanoparticles and obtained a novel Fe_1_–Ni_1_–N–C catalyst, which exhibited superior performance of CO selectivity (Figure 27).^470^ Theoretical calculation showed that single Fe atoms can be activated by adjacent single Ni atoms via nonbonding interactions, resembling the process of CO_2_ reduction in CODH. It is worth noting that the process of pyrolysis cannot precisely control the distance between Ni and Fe atoms.

In conclusion, better catalytic performance can be achieved by introducing the active center of dehydrogenase into MOFs. However, there is still a long way to mimic the unique coordination sphere of the active center around dehydrogenases, which might achieve even better catalytic performance and further understand the mechanism of the catalytic cycles.

### 3.4 Carbonic-Anhydrase-Inspired MOFs

As discussed before, CO_2_ capture and utilization is a universal issue, which addresses the challenges brought by global warming. Many research groups provide their strategies toward CO_2_ fixation by designing different compounds with affinity to bind with CO_2_. However, nature offers many ways to balance the CO_2_ level, including metalloenzymes. Herein, we will mainly focus on carbonic anhydrases (CAs) to offer new strategies for MOF-based CO_2_ adsorbents.

**Figure 28:**
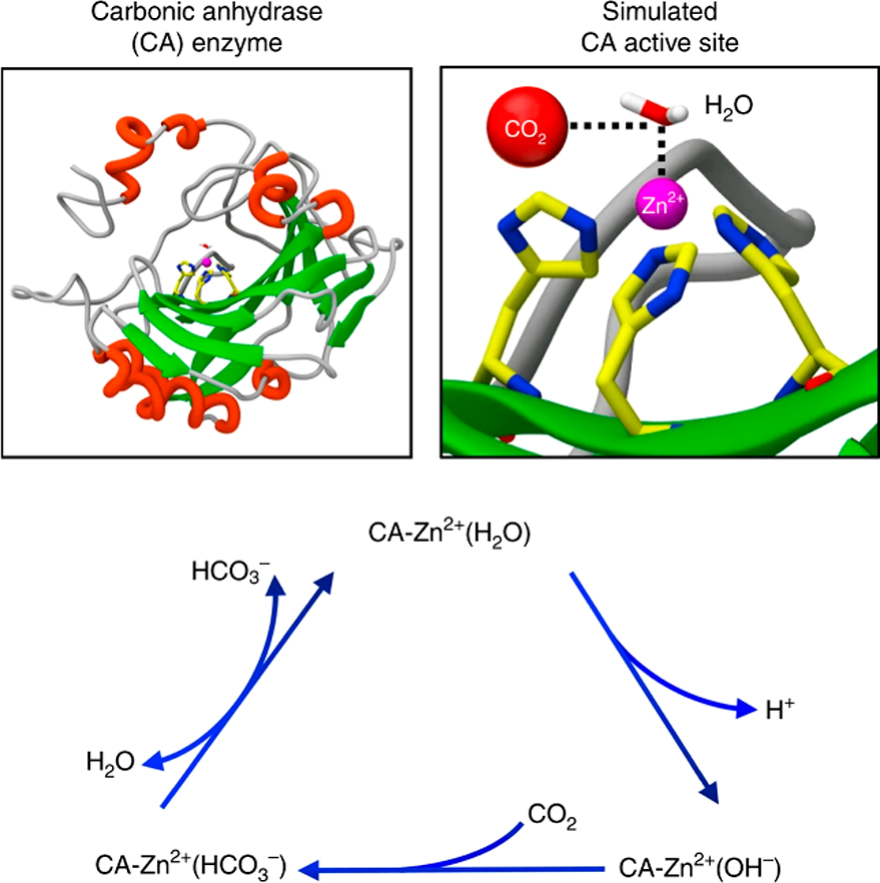
Structural illustrations and CO_2_ capture mechanism of carbonic anhydrase. Reproduced with permission from ref (472). Copyright 2018 Springer Nature.

CAs are widely found in marine and terrestrial ecosystems that can biomineralize CO_2_ by forming CaCO_3_ crystals with a TOF of 10^4^–10^6^, one of the fastest rates among all enzyme catalysis.^471^ All the members of the CA family contain the same active center, one distorted tetrahedral Zn center coordinated to three histidine imidazole residues and an aqua ligand (Figure 28).^472^ The aqua ligand plays an essential role in the catalytic cycles, providing an OH unit to furnish the bicarbonate ion (HCO_3_^–^) from molecular CO_2_.^473^ The imidazole coordinated Zn (II) ion drags the electron cloud of the Zn–H_2_O bond, which polarizes the O–H bond and leads to a swift removal of hydrogen. Therefore, researchers focus on creating a suitable environment to form a more efficient Zn–OH center.

**Figure 29:**
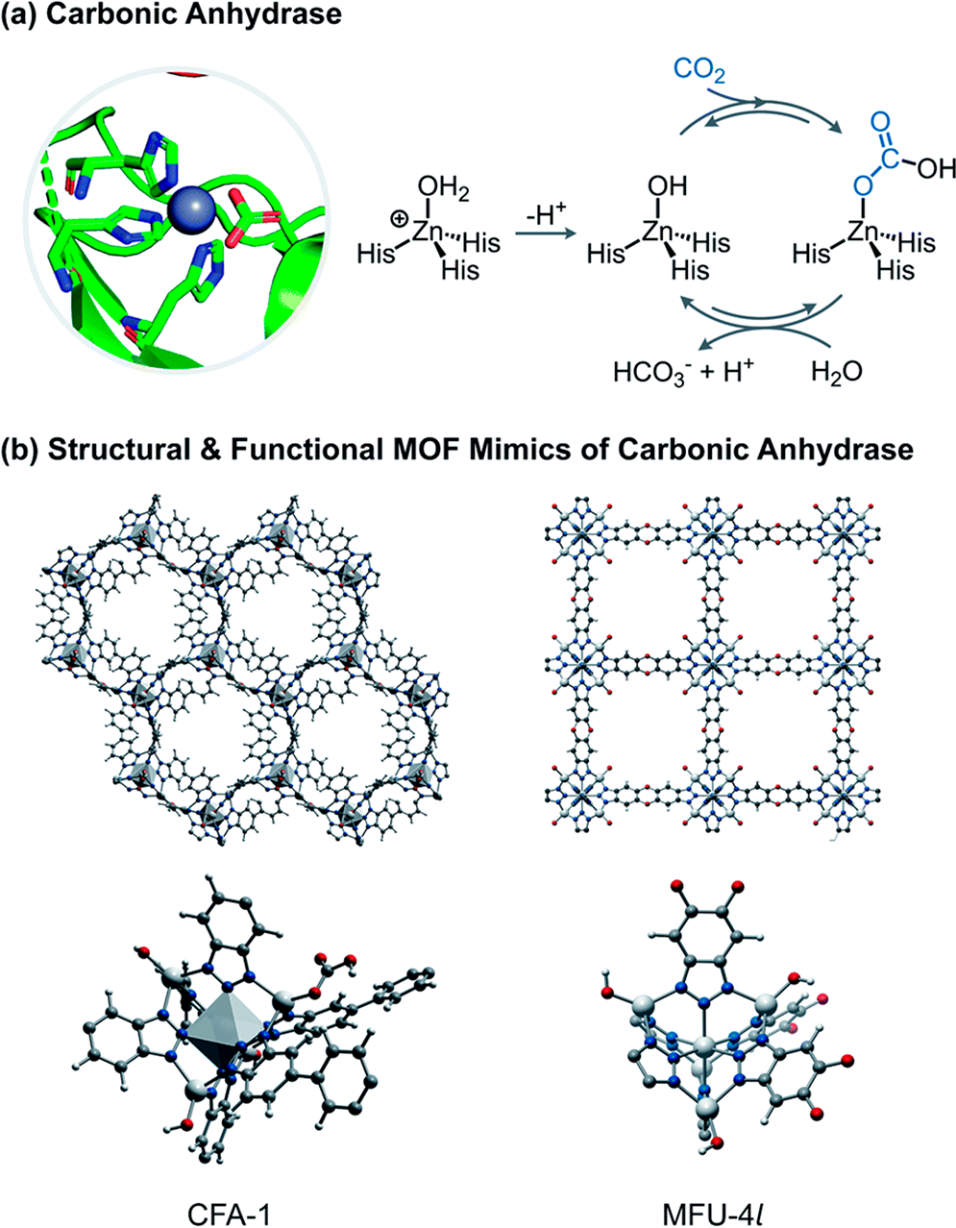
Two triazolate-based MOFs as mimics of carbonic anhydrase. (a) Active site of carbonic anhydrase. (b) Structures of CFA-1 and MFU-4l with exposed Zn sites. Reproduced with permission from ref (476). Copyright 2020 Royal Society of Chemistry.

Two triazolate-based MOFs were reported in 2011 and 2013 separately, Zn_5_ (OH)_4_(bibta)_3_(CFA-1-(OH), H_2_bibta = 5,5^’^-bibenzotriazole) and Zn_5_(OH)_4_ (btdd) (MFU-4l-(OH), H_2_btdd = bis(1,2,3-triazolo[4,5-*b*],[4^’^,5^’^-i]) dibenzo[1,4]dioxin).^474,475^ Both MOFs feature SBUs that are close structural homologues of the CA active site, with central octahedral zinc(II) connecting six bridging azolate ligands and four peripheral tetrahedral zinc(II) sites with an exchangeable X-ligand (Figure 29).^476^ In the next few years, more examples of MOFs were reported to feature similar active centers to CA and enable CO_2_ capture and storage (CCS). Zhang and co-workers designed MAF-X25 and MAF-X27 MOF series,^219^ functionalized with monodentate hydroxide by reacting redox-active metals in M^II^_2_Cl_2_ (bbta) (M = Mn, Co; H_2_bbta = 1*H*,5*H*-benzo (1,2-d:4,5-*d*^′^) bistriazole) with hydrogen peroxide. Notably, the functionalized MOFs can achieve ultrahigh CO_2_ adsorption heat (124 kJ mol^–1^) and adsorption capacity (9.1 mmol cm^–3^ at 298 K and 1 bar). Infrared (IR) spectroscopic analysis of the CO_2_-loaded material revealed formation of a bicarbonate moiety similar to the intermediate in the catalytic cycle of CA CO_2_ activation, which explains its ability to capture CO_2_ at a high relative humidity (82%). Lee and co-workers also designed a NNN-pincer-based complex (Co-BBP) mimicking the active site of CA, which could be immobilized to a Tb-based MOF.^220^ Interestingly, instead of the tetrahedral environment of the CA’s active center, the ligand BBP coordinates Co in a pendant fashion. Though the Co(II)-based catalyst adopts a different structural geometry, it was found to function as a suitable mimic of CA. Dimerization of the homogeneous catalyst was successfully ceased by immobilization in Tb-based MOF, providing a new footprint for CO_2_ capture and storage. Compared to direct enzyme immobilization, introducing functional linkers into MOFs can result in a higher density of active centers. Therefore, introducing proper NNN-pincer-based molecules to MOFs may generate higher performance in CO_2_ utilization.^383^

**Figure 30:**
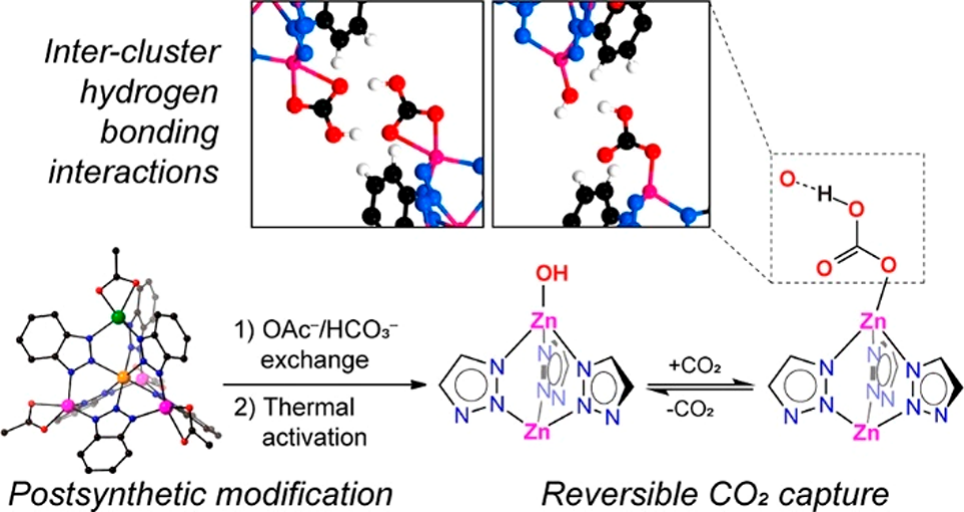
CO_2_ chemisorption on the Zn–OH site through intercluster hydrogen bonding interactions. Reproduced with permission from ref (221). Copyright 2018 American Chemical Society.

In 2018, Wade and co-workers directly prepared CFA-1-(OH) by ligand exchange procedure followed by thermal activation to generate additional nucleophilic Zn–OH groups on the MOF,^221^ which resemble the active site of α-carbonic anhydrase, and it exhibited excellent performance for trace CO_2_ capture. More importantly, the structure analysis and mechanism study revealed that the Zn–X sites in the SBU were not all equivalent, where bicarbonate may interact with a second Zn–OH group or Zn–CO_3_H group at an adjacent SBU when undergoing CA chemistry (Figure 30). Inspired by the achievements of CA-mimicking MOFs in CO_2_ capture and utilization, more studies have been reported to uncover the adsorption and catalysis mechanism in MOFs. Dinu and co-workers reported that hydrophilic MOFs retained 72% activity of the free CA,^202^ while the hydrophobic ones only retained about 28%. It indicated that water plays a crucial role in the CO_2_ hydration process and acts as both the reactant and stabilizer. Moreover, Zhang and co-workers also suggested ZIF-100 as an alternative for CA mimicking MOFs,^222^ enabling the hydrolysis of *para*-nitrophenyl acetate. As another promising candidate for CA mimicking MOFs, MFU-4l differs from the former CFA-1-(OH) in being a cubic MOF with equally spaced SBUs and N_3_ZnX sites. Similar to Wade’s postsynthetic modifications on CFA-1-(OH), Dinča and co-workers installed terminal hydroxides to MFU-4l by anion exchange with [^*t*^Bu_4_N][OH].^223^ Mechanism studies demonstrated that MFU-4l-(OH) enabled catalyzing the isotopic exchange between H_2_^18^O and CO_2_ as well as hydrolyzing *para*-nitrophenyl acetate. Different from the mechanism in CFA-1-(OH), CO_2_ was bonded in this MOF through an insertion into the Zn–OH bond, leading to remarkable adsorption of CO_2_ (3.41 mmol/g). Recently, extensive studies have be conducted to explore broader usage of CA mimicking MOFs. For instance, Dong and co-workers synthesized a ZIF-8 nanozyme promoting CO_2_ hydration and acetylthiocholine hydrolysis.^477^

Incorporating active sites into MOFs can create a platform with high-density and well-isolated catalytic centers. Nevertheless, there are some limitations in developing CA mimicking MOFs. For instance, both SBUs of MFU-4l-(OH) and CFA-1-(OH) possess four accessible Zn–OH motifs, which are not fully electronically isolated from other Zn centers according to CO_2_ sorption measurements. Therefore, the potential interplay between multiple metal centers should also be considered for the overall catalytic performance. Moreover, some solution-phase CA models enable selectively CO_2_ binding through a carboxylate intermediate, in which the carboxylate group bridges two zinc centers in a unidentate coordination mode.^478^ Such cooperative binding mode may be essential to the CA’s catalytic performance but has long been neglected in MOF studies. Presumably, it is promising to develop CA mimicking MOFs featuring cooperative coordination behavior to improve CO_2_ capture and utilization capacity.

### 3.5 Nitrogenase-Inspired MOFs

The conversion of atmospheric dinitrogen (N_2_) to bioavailable ammonia (NH_3_) is a critical step in the biogeochemical nitrogen cycle, which is highly related to the agriculture and chemical industry. Although nitrogen is an essential component of amino acids, nucleobases, and many biorelated molecules in nature, nitrogen is intricated to be directly utilized by organisms owing to the strong N≡N bond. Currently, nitrogen fixation mainly occurs in three ways, biological catalysis based on the nitrogenase family,^479−481^ Haber–Bosch process,^482,483^ and light-induced chemical conversion.^484^ It is worth noting that nitrogenase is the only biological system capable of generating ammonia directly from dinitrogen under ambient conditions. There are mainly three variants of nitrogenase, which are Mo-dependent, V-dependent, and Fe-dependent nitrogenase. Despite decades of research on nitrogenase, the catalytic mechanism and the active center’s structure are still under debate. Moreover, the nitrogenase’s active center is very vulnerable to water and oxygen, bringing difficulty in characterization when encapsulating or mimicking the active center in framework materials.^485^ This section will mainly focus on the Mo-dependent nitrogenase and its mimics in MOF systems.

**Figure 31:**
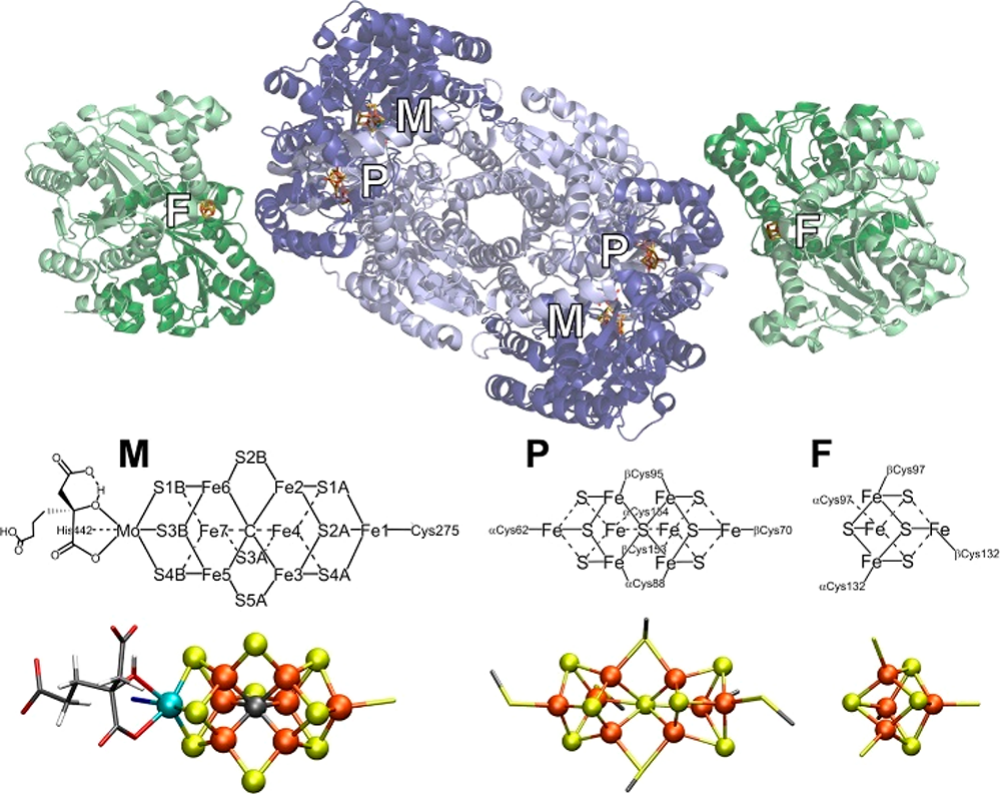
Subunits and cofactors of Mo nitrogenase. Reproduced with permission from ref (489). Copyright 1994 American Chemical Society.

The nitrogenase consists of two-component proteins, the MoFe protein and the Fe protein, also named dinitrogenase/component I and dinitrogenase reductase/component II (Figure 31).^486−489^ The Fe protein contains Fe_4_S_4_ cluster (F cluster), involved in the process of electron transportation.^486^ MoFe protein contains two metal clusters, the iron–molybdenum cofactor (FeMo-co, M-cluster) and P-cluster.^490,491^ The iron–molybdenum cofactor provides the active site for substrate binding and reduction, while the P-cluster enables electron transfer from the Fe protein to FeMo-co.^492^ In the alternative V- and Fe-type nitrogenases, the Mo of FeMo-co is replaced by V or Fe,^493^ which leads to varied performances in nitrogen fixation.

The electronic structures of FeMo-co remain vague. Although Mo^IV^ was widely accepted as the oxidation state of the molybdenum, recent studies propose reassigning the oxidation state to Mo^III^.^494^ Moreover, the valence of Fe atoms is not identical in FeMo-co. Studies have shown that some iron atoms in the FeMo-co are partially reduced, but the specified valence of iron is still under debate.^495^ According to X-ray absorption spectroscopy, the oxidation state of the FeMo-co is Mo^III^-3Fe^II^-4Fe^IV^, while density functional theory (DFT) calculations have suggested that the formal oxidation state is Mo^IV^-2Fe^II^-5Fe^III^-C^4–^-H^+^.^496,497^

Polypeptides constructing the coordination sphere surrounding FeMo-co are vital in the catalytic cycle.^498^ The Fe atom of FeMo-co is coordinated to the sulfhydryl group of Cys_275_, while the Mo atom is coordinated to the imidazole nitrogen of His_442_ and the carboxyl oxygen of homocitrate. The coordination bonds stabilize the overall structure and affect the catalytic properties. The homocitrate coordinated with the molybdenum can form hydrogen bonding with the imidazole group, which is the only known acid enabling proton transportation in the nitrogenase, compared with the citric acid bearing methylene group.^499^

To date, chemists developed various methods to attain the FeMo-co of the nitrogenase, including extraction, chemical synthesis,^500^ and biosynthesis.^501,502^ As the most promising strategy, extraction consists of acid-treated and nonacid-treated types, requiring strict air-sensitive operations.^491,503−507^ However, extraction always results in NMF-substituted FeMo-co, and further purification is required.^501,508^

High-resolution crystal analysis indicated that polypeptide chains around the FeMo-co enable blocking water from the active site.^509^ It is also postulated that there is a water chain containing eight water molecules, providing a pathway for transporting protons from the outer sphere to the FeMo-co. Besides, the exact substrate binding sites of the three metal-centered catalytic cofactors, FeMo-co, FeFe-co, and VFe-co, are still uncertain. For the FeMo-co, the amino-acid-involved catalysis indicates that the active site is located at the Fe–S face. However, determining the location and binding mode of dinitrogen within the nitrogenase remains a grand challenge.^510^ To date, one widely accepted mechanism is the Lowe–Thorneley catalytic cycle.^511^ Adamo,^512^ Dance, and Seefeldt conducted comprehensive studies on the Lowe–Thorneley catalytic cycle, which could serve as a starting point for further research.^498,513,514^

Owing to the astonishing catalytic performance of nitrogenase, researchers have developed a large number of molecular catalysts for nitrogen fixation, which can be encapsulated into MOFs as guest molecules. Among all molecular catalysts, molybdenum was long thought to be the essential transition metal for nitrogen fixation. Schrock and co-workers first reported a single Mo-based molecular catalyst,^515^ which could reduce N_2_ to ammonia at ambient conditions through a distal type mechanism. Following this work, a di-Mo-based catalyst with PNP-type pincer ligands was reported by Nishibayashi.^516^ Zuo and co-workers also designed a diiron complex similar to the active center of nitrogenases,^517^ which can accommodate HN=NH and convert it to NH_3_. Recently, Qu and co-workers reported a well-defined thiolate-bridged Fe^IV^Fe^IV^ μ-nitrido complex featuring an uncommon bent Fe–N–Fe moiety, which showed excellent reactivity in hydrogenation with N_2_, forming ammonia at ambient conditions with high yield.^518^

**Figure 32:**
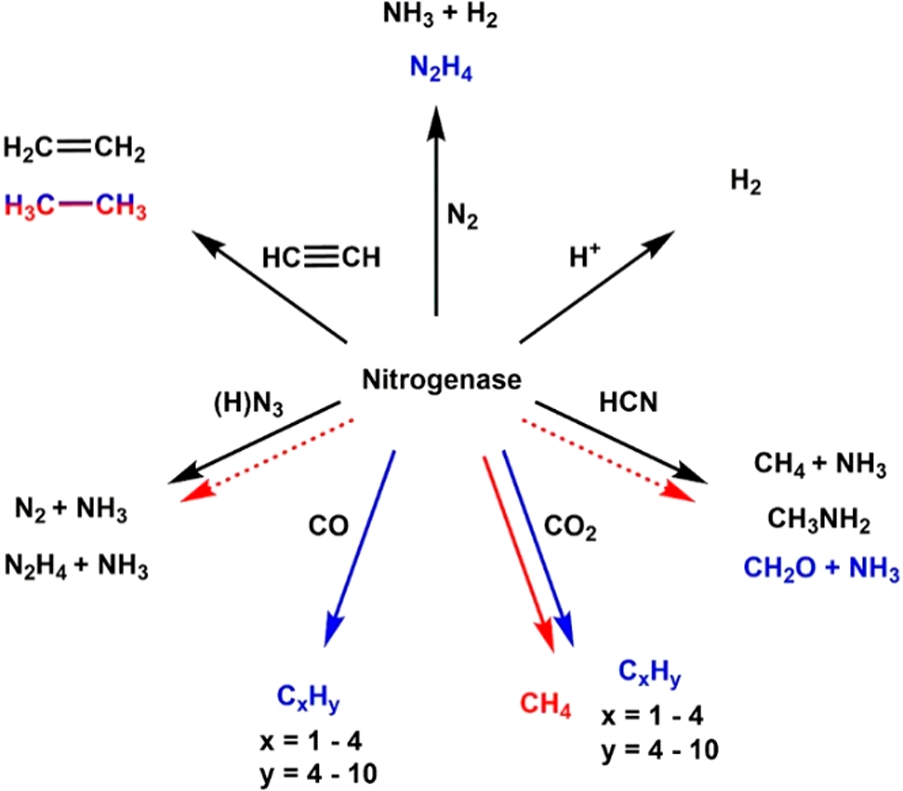
Brief summary of catalysis reaction conducted by alternative nitrogenases. Reproduced with permission from ref (523). Copyright 2020 American Chemical Society.

Comprehensive studies have been conducted on the reactivity of nitrogenases. N_2_ is reduced using 8 H^+^ and 8 e^–^ equivalents in Mo-depended nitrogenase, as one equivalent of H_2_ is produced along with an equal ratio of N_2._ To further explore the substrate scope of nitrogenase, N_2_ analogues such as acetylene (C_2_H_2_), carbon monoxide (CO), hydrogen cyanide (HCN), azide (N_3_^–^), nitrite (NO_2_^–^), and nitric oxide (NO), unsaturated cyclic compounds (cyclopropene, diazirine), and alkyne species with terminal triple bonds such as propyne (HC≡C–CH_3_) and propargyl alcohol (HC≡C–CH_2_–OH)^514,519−522^ have been tested (Figure 32).^523^ Ribbe also gave a comprehensive summary of nitrogenases with altered active centers, suggesting V- and Fe-only nitrogenases are more versatile small molecule reductases than Mo-only nitrogenases. Given the wide substrate scope of nitrogenases, some researchers tried introducing the nitrogenase or its active centers into electrodes for electrochemical applications.^524,525^ As moisture- and air-sensitive enzymes, surprisingly, nitrogenases are able to rapidly switch structures by shifting the enzyme into an inactive but oxygen-tolerant state, providing enlightenment to design switchable nitrogenase-mimicking catalysts.^526−529^ In conclusion, nitrogenase enables reducing dinitrogen N_2_ to ammonia NH_3_ under ambient conditions and serves as a multifunctional catalyst for many gas molecules. Though the mechanisms and structures of FeMo-co are still under debate, it is promising to reproduce its function by creating a similar coordination sphere or introducing other nitrogenase-mimicking catalysts into the MOF system.

MOFs have long been utilized as a platform for energy conversion, such as hydrogen evolution reaction (HER), oxygen evolution reaction (OER), nitrogen reduction reaction (NRR), and carbon dioxide reduction reaction (CO_2_RR). Owing to the high structural tunability and redox-active nature, pristine MOFs have been recognized as promising electrocatalysts for NRR.^530^ There are mainly three strategies to construct MOFs for NRR, including (a) creating defects in pristine high electron conductivity MOFs as NRR catalysts,^531−534^ which can serve as Lewis acid to enhance the active sites to withdraw π-electrons from N_2_ molecules and diminish the N≡N bonds; (b) introducing other functional materials like metal nanoparticles into MOF as NRR catalysts,^535^ which can overcome the MOFs’ lack of conductivity and prone to HER;^536,537^ (3) developing MOF-derived materials, such as MOF-derived carbon,^538−541^ metal oxides,^542^ and single atom catalysts (SACs).^543,544^

**Figure 33:**
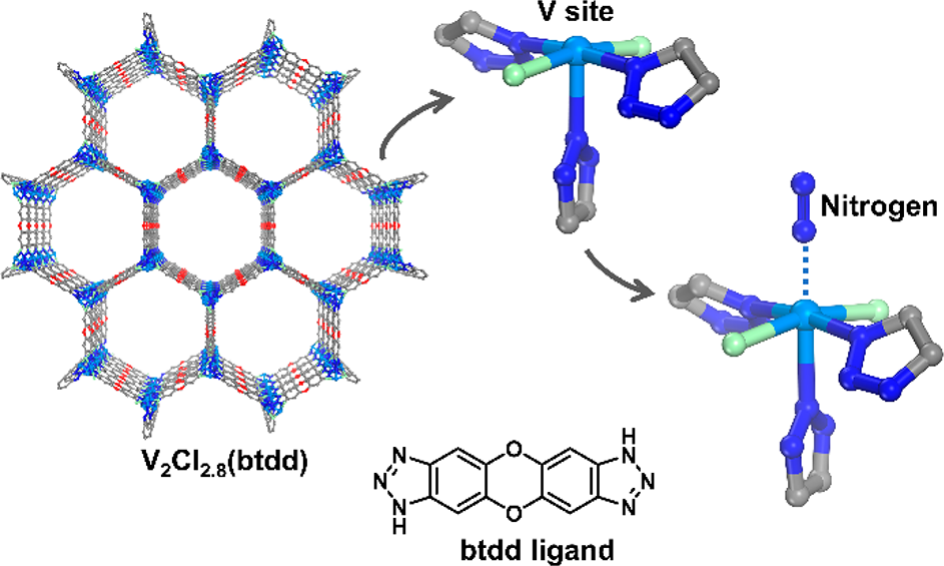
Structural illustration of a MOF V_2_Cl_2.8_ (btdd) with accessible V site to bond nitrogen.

Although the structure and catalytic mechanism of nitrogenases have been extensively studied, to our knowledge, there are no reported MOFs that can be described as an exact mimic of nitrogenase. Herein, some works featuring nitrogen fixation performance can be the first footprint to develop nitrogenase-mimicking MOFs. Sun and co-workers designed a novel 2D conductive MOFs based on molybdenum,^224^ enabling converting N_2_ into NH_3_ at room temperature with a very low overpotential of 0.18 V. The coordination sphere of Mo partially resembled the environment of Mo in FeMo-co, providing an efficient NRR electrocatalyst. Long and co-workers developed a MOF with exposed vanadium(II) centers,^225^ which can back-donate electrons to weak π acids for isolating N_2_ from other gases (Figure 33). The unsaturated vanadium(II) centers mimic FeV-co, especially in their electronic structures, leading to its excellent performance in separating N_2_. Jiang and co-workers deliberately designed MIL-53 (Fe^II^/Fe^II^) (MIL = Material from Institute Lavoisier) to mimic the mixed-valence metalloclusters in FeMo-co nitrogenases.^226^ The Fe^II^/Fe^III^ ratio was regulated from 0.18:1 to 1.21:1 by varying the addition amount of ethylene glycol (EG), resulting in an optimal Fe^II^/Fe^III^ ratio (1.06:1) to achieve the highest ammonia evolution rate up to 306 μmol h^–1^ g^–1^. In addition, they further mimicked the relationship between P and M clusters in nitrogenase to design MOFs with an active center and electron buffer tank to improve electron transfer and nitrogen fixation utility.^227^

**Figure 34:**
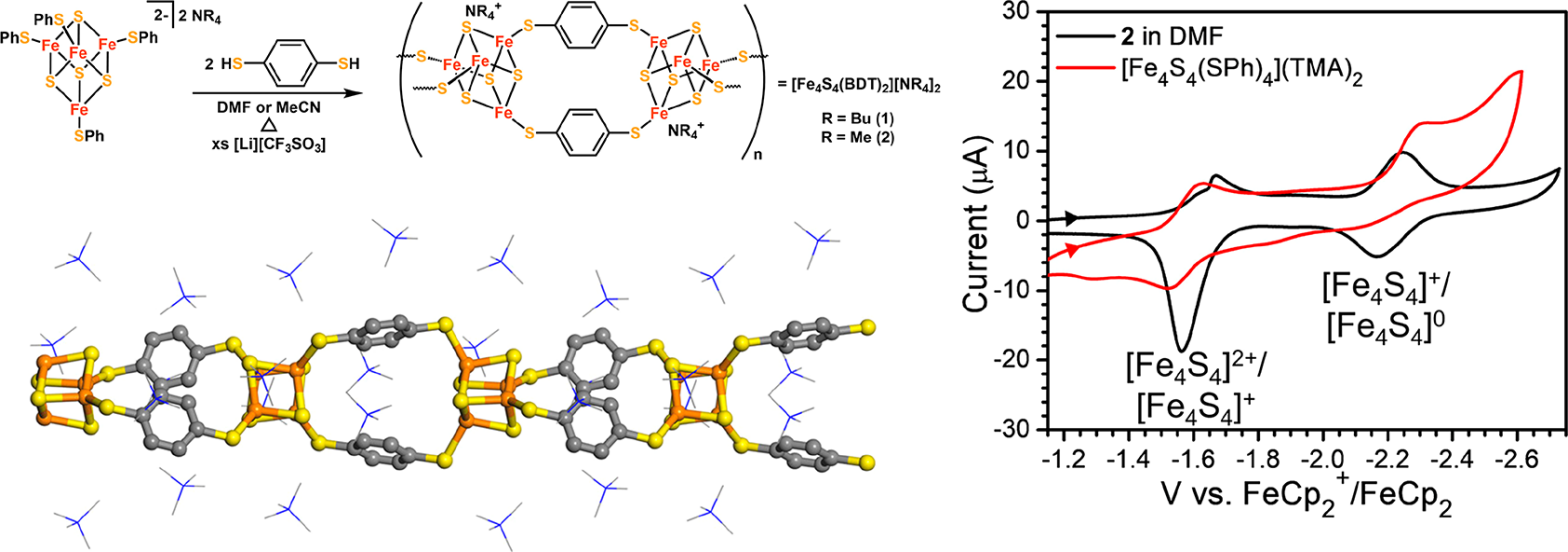
Synthesis of [Fe_4_S_4_]-based redox-active coordination polymers. Reproduced with permission from ref (228). Copyright 2019 American Chemical Society.

[Fe_4_S_4_] cluster in nitrogenase serves as an electron donor to transfer electrons concomitant with ATP hydrolysis to the cofactor. As a well-recognized electron transfer center, [Fe_4_S_4_] clusters are pervasive in all forms of life. Since 50 years ago, Holm and co-workers have made remarkable contributions to the controlled synthesis of [Fe_4_S_4_] analogues.^10,545,546^ In 2019, Anderson and co-workers first incorporated the [Fe_4_S_4_] cluster into a coordination polymer (Figure 34).^228^ Once charge carriers are introduced by reducing the [Fe_4_S_4_] clusters, the electrical conductivity of the material could be increased by up to 4 orders of magnitude. Moreover, the substitution effect on the electronic structures was studied by replacing the ligand with either 2,5-dimethyl-1,4-benzenedithiol (DMBDT) or 2,3,5,6-tetramethyl-1,4-benzenedithiol (TMBDT).^229^ A recent report indicated that the [Fe_4_S_4_] cluster of a soil bacterium, *Azotobacter vinelandii*, enabled catalyzing the conversion from CO_2_ to CO. The Fe protein serves as a reductase that can work under in vitro conditions with a strong reductant presented. Recently, Hu and co-workers systematically summarized C1-substrate reduction and corresponding mechanistic studies using protein-bound and free [Fe_4_S_4_] clusters, uncovering the application potentials of [Fe_4_S_4_] clusters.^547^

In conclusion, nitrogenases are vital to dinitrogen fixation and other small molecular activation. Studies on reproducing the nitrogenase’s active center in MOFs are still limited, which could be a promising direction in catalysis, mechanism study, and material design. The achievements in the nitrogenase-mimicking MOFs have a high potential to provide a deeper understanding of the mechanism and functions of nitrogenases.

### 3.6 Hydrogenase-Inspired MOFs

Given the increasing threat from global warming, developing sustainable and clean energy is closely associated with the future of humans. H_2_, with high combustion enthalpy and clean products, is widely recognized as the most promising eco-friendly fuel.^548,549^ However, the industrial production of hydrogen is always energy-consuming and high-cost, involving electrolysis of water–methane pyrolysis,^550−553^ and steam methane reforming,^554^ which fails to meet the requirement of the green economy. Therefore, it is urgent to develop a novel hydrogen production approach. Interestingly, it is estimated that 99% of organisms have molecular hydrogen metabolic functions. Most of these species are microorganisms, including bacteria, archaea, cyanobacteria, and some eukaryotes, such as protozoa. Their ability to metabolize H_2_ comes from the expression of metalloenzymes known as hydrogenase *in vivo*.^555^ Hydrogenase is a series of metalloenzyme containing metal elements such as iron and nickel, which can catalyze the reversible oxidation of H_2_.^556^ Although hydrogenases are usually sensitive to oxygen, some of them can catalyze hydrogen cycling in the presence of oxygen.^557,558^ Intense research has been devoted to the mechanism of O_2_ inactivation of hydrogenases.^559,560^

**Figure 35:**
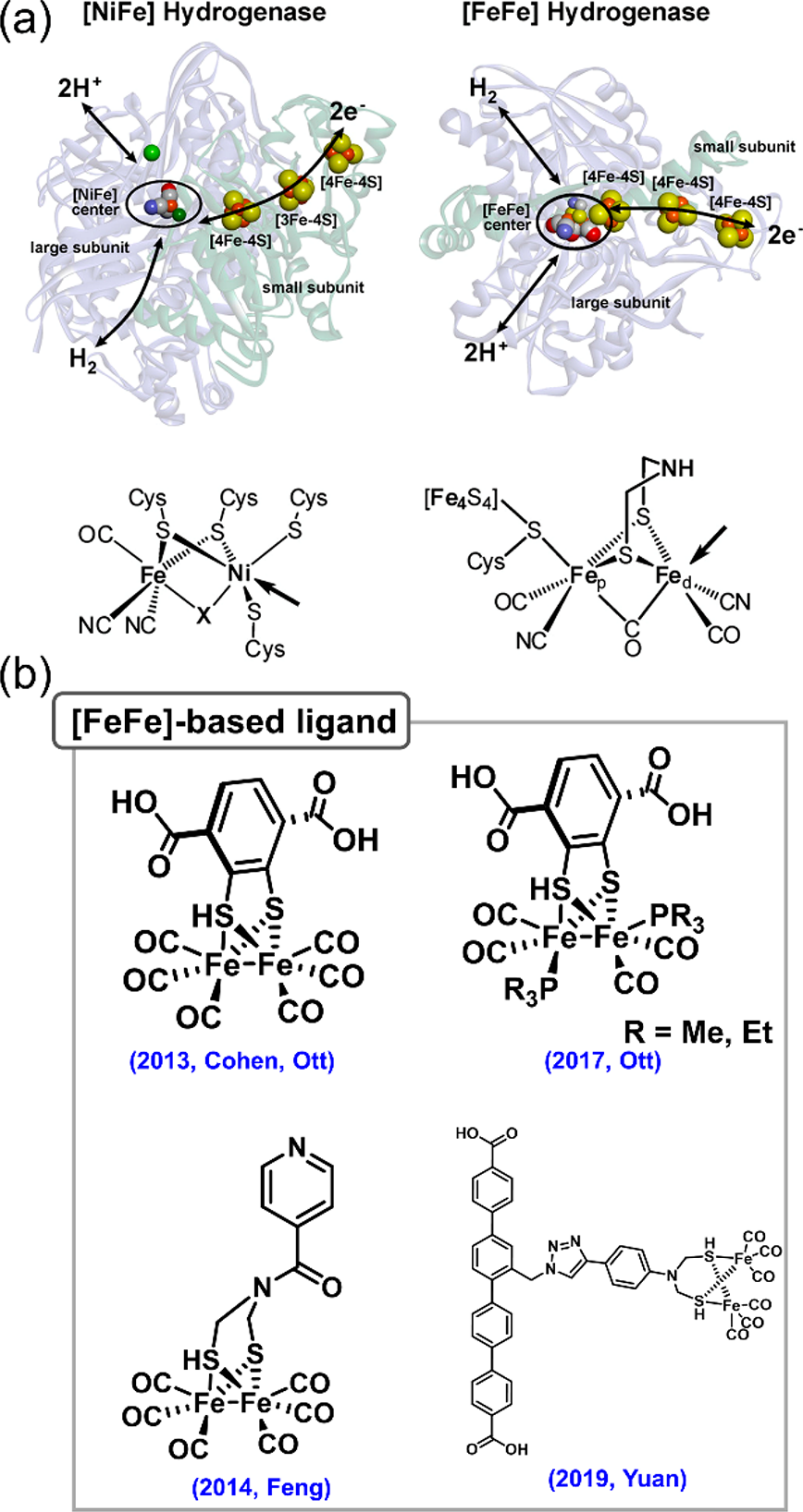
Illustration of hydrogenases and hydrogenase-mimicking ligands. (a) Active sites of [NiFe]–H_2_ases and [FeFe]–H_2_ases. (b) Ligand design of [FeFe]–H_2_ase-mimicking MOFs. Reproduced with permission from ref (561). Copyright 2014 American Chemical Society.

The three most naturally abundant hydrogenases are [Fe]–H_2_ases, [NiFe]–H_2_ases and [FeFe]–H_2_ases, according to the different metal compositions of the active centers (Figure 35a).^561−563^ [FeFe]–H_2_ases are the most efficient H_2_ generator with a reported TOF of ∼6000–9000 s^–1^ per site.^564^ The [FeFe] and [NiFe] hydrogenases are redox catalysts, driving H_2_ oxidation and proton (H^+^) reduction at a very high rate without any overpotential.^565^ However, the [Fe] hydrogenases are only found in archaea methanogens, possessing a fundamentally different enzymatic mechanism in terms of redox activity and electron transfer.

While [FeFe]–H_2_ases vary in size (45–130 kDa), all of them possess an active site known as the H-cluster,^563,566^ in which the binuclear Fe center [FeFe] shares a cysteine ligand with a [4Fe–4S] cluster. The two Fe centers are bridged by an azadithiolate (ADT) and a μ_2_-CO linker, which are terminated with CO and CN ligands. The diiron cluster is coupled with a Fe_4_S_4_ ferredoxin cluster via a cysteinyl residue. It should be noted that the hydrogen bonds around CN ligands are crucial to determine the orientation of the diiron cluster.^567,568^ In the H_ox_ state, the distal Fe is in an octahedral coordination environment, while in the H_red_ state, the distal iron will adopt a square pyramidal geometry bearing an open coordination site.^569^ The H^–^/H_2_ binding takes place on the reactive site of distal iron, which may coordinate with exogenous CO and lead to the deactivation of the enzyme.

The [NiFe]–H_2_ases are more abundant and O_2_-tolerant than the [FeFe]–H_2_ases.^556,570^ Structural characterization of [NiFe] hydrogenases reveals that these enzymes consist of two subunits.^571,572^ The smaller unit contains three aligned iron–sulfur clusters. The larger subunit accommodates the redox-active Ni(S-Cys)_4_ center, of which two S-Cys ligands bridge to a redox-inactive Fe-(CN)_2_ (CO) fragment.^573^ Interestingly, the Ni adopts a seesaw geometry similar to the SF_4_ molecule, and the (Cys-S)_2_Fe(CN)_2_ (CO) center resembles a distorted pyramid. In some [NiFe] hydrogenases, one of the Ni-bound cysteine residues is replaced by selenocysteine.^574,575^ Besides, the metals are separated precisely with a distance of 2.57 Å comparable to the sum of the Ni and Fe covalent radii, which is significant for the catalytic activity.^576,577^ Though several mechanisms for H_2_ uptake in [NiFe]–H_2_ases had been proposed, a consensus has never been reached.^578^

[Fe]–H_2_ase is not a redox enzyme and does not contain Fe–S clusters, which requires a hydride acceptor/donor substrate to react with or produce H_2_.^579−581^ Historically, its active center was once regarded as a free iron cation.^582^ [Fe]–H_2_ase has an active center around a Cys-ligated Fe-guanylylpyridinol cofactor (Fe-GP).^583,584^ Unlike the [FeFe]–H_2_ases and [NiFe]–H_2_ases, [Fe]–H_2_ases enable heterolytic splitting of H_2_ into a hydride and a proton. The as-formed hydride will transfer to the carbocation-containing substrates, methenyl-tetrahydromethanopterin (methenyl-H_4_MPT^+^), producing methylene-tetrahydromethanopterin (methylene-H_4_MPT).^585^ The iron center remains EPR-silent throughout the catalytic cycle, speculated to be a low spin Fe(II) center acting as a Lewis base for H_2_ coordination and activation. Crucial experimental evidence is still limited to support the proposed mechanisms.^568^

The current studies of hydrogenases and their enzymatic reactions can provide a solid theoretical basis and guidance to develop “artificial hydrogenases” for future hydrogen production.^561^ MOFs feature highly porous structures and open channels, making the active sites readily accessible for substrates. In recent years, MOFs have attracted continuous attention as photocatalysts for visible-light-driven H_2_ production.^586−588^ Compared to traditional photocatalysts, such as TiO_2_ and g-C_3_N_4_, MOFs have unique advantages in structural tunability and porosity.^234^ Nevertheless, studies on hydrogenase-mimicking MOFs are still limited. Many publications have highlighted the superiority of using MOFs and MOF-derived materials for the photoreduction of water.^589−591^ The framework confinement of MOFs provides a high density of active sites without the issue of agglomeration. In addition, the porous nature of MOFs/MOF-derived materials can separate the photoexcited electron–hole pairs and provide extra pathways for the photoexcited electron migration, thus facilitating the migration of the charge carrier. Among all photocatalytic MOFs, metal-sulfide-based MOFs usually feature the best performance,^592^ in which the catalytic sites resemble the active centers of hydrogenase. Therefore, it is promising to mimic the hydrogenase to improve hydrogen production performance. This section will summarize the state-of-the-art research on introducing hydrogenases’ active centers into MOFs.

#### 3.6.1 [FeFe]-Based MOFs

**Figure 36:**
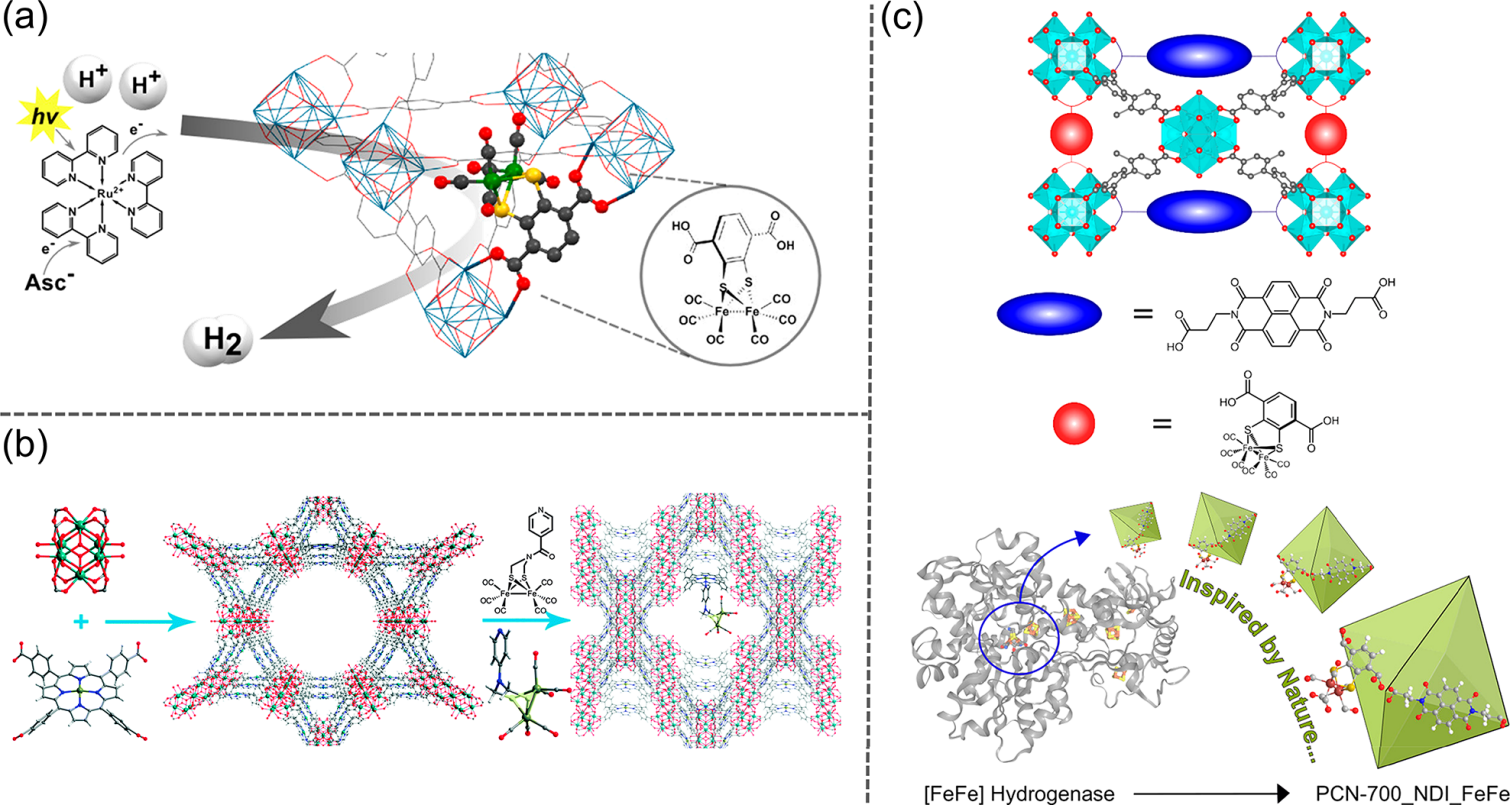
Three strategies to synthesize [FeFe]–H_2_ase-mimicking MOF. (a) Introducing [FeFe] ligand through ligand exchange to afford a multivariate MOF enabling photochemical hydrogen production. Reproduced with permission from ref (230). Copyright 2013 American Chemical Society. (b) Installing [FeFe] model compounds to accessible metal sites in a porphyrin MOF. Reproduced with permission from ref (232). Copyright 2014 Royal Society of Chemistry. (c) Integrating redox-active ligands and [FeFe]–H_2_ase active sites into a defective MOF through linker installation to mimic the electron transport chain. Reproduced with permission from ref (235). Copyright 2021 American Chemical Society.

There are mainly two design strategies for hydrogenase-mimicking MOFs: (1) Incorporating proton reducing agents into photocatalytic MOFs, and (2) designing MOFs featuring proton reducing groups and employing an external photosensor to initialize the reaction (Figure 35b). The first [FeFe]–H_2_ase-mimicking MOF was reported by Cohen, Ott, and co-workers^230^ (Figure 36a). A Zr-based MOF UiO-66 was selected as the pristine framework owing to its superior chemical stability. To overcome the inherent lability of the organometallic units, a postsynthetic modification method was employed to incorporate the [FeFe](dcbdt)(CO)_6_ units into the UiO-66. In conjunction with a photosensitizer [Ru(bpy)_3_]^2+^ and an electron donor ascorbate, the UiO-[FeFe](dcbdt)(CO)_6_ could catalyze photochemical hydrogen evolution in water at a pH of 5, of which the catalytic performance exceeded the homogeneous system in terms of rate and total hydrogen production yield. Cohen and co-workers also reported a facile approach to fabricating [FeFe](dcbdt)(CO)_6_-incorporated UiO-66 films with exceptionally high crystallinity and tunable thickness on a transparent and conductive glass substrate.^231^ Later in 2014, Feng and co-workers combined reducing groups and photocatalytical centers in another stable zirconium-porphyrin MOF.^232^ A homogeneous complex [(ı′-SCH_2_)_2_NC(O) C_5_H_4_N]–[Fe_2_(CO)_6_] was introduced into a highly robust zirconium-porphyrin based MOF PCN-222, in which the ZnTCPP serves as the photosensitizing center and the [(ı′-SCH_2_)_2_NC(O)C_5_H_4_N]–[Fe_2_ (CO)_6_] is structurally analogous to [FeFe]–H_2_ases’ active centers (Figure 36b). The cooperation between the photosensitizer and the hydrogen-evolution catalyst enhanced the stability of the [FeFe] catalyst and the efficiency in photochemical hydrogen evolution. Based upon Cohen’s MOF system, Ott and co-workers systematically investigated different model molecules of [FeFe]–H_2_ases,^233^ in which the CO ligands of [FeFe](dcbdt)(CO)_6_ are partially substituted by phosphines (PX_3_, X = Me, Et, Ph). They found that smaller phosphines (PX_3_, X = Me, Et) indicated higher selectivity compared to analogous reactions in the solution phase. Moreover, the [Fe_2_ (dcbdt)(CO)_4_ (PX_3_)_2_] complexes installed in the UiO-66 matrix behave much more like typical [FeFe] hydrogenase active site than the discrete [FeFe](dcbdt)(CO)_6_, which increased the electron density at the Fe_2_ sites and potentially allowed the formation of hydride species. This strategy provides a novel perspective to fine-tune the charge density at the active centers. Yuan and co-workers developed a novel approach to incorporating photosensitizer and catalyst molecules together into another UiO-type MOF.^234^ The UiO-MOF was constructed from two dicarboxylate ligands, in which a [Ru(bpy)_3_]^2+^-derived dicarboxylate ligand served as the photosensitizer and an azide-modified dicarboxylate ligand allowed linking the [Fe_2_S_2_] catalyst through covalent bonds. In 2021, Ott, Cohen, and co-workers synthesized a redox-active MOF derived from PCN-700 that featured both a biomimetic model of the [FeFe]–H_2_ase active site and a redox-active linker as an electron mediator,^235^ thereby mimicking the function of [4Fe4S] clusters in [FeFe]–H_2_ase (Figure 36c). Given the fact that the overall catalytic efficacy is often limited by charge transport, the difference in charge transport between in the MOFs and the enzyme were discussed, suggesting that additional mediator linkers with a higher electron self-exchange rate (*D*_e_^app^) than the catalyst linker would result in a better performance.

#### 3.6.2 [NiFe]-Based MOFs

Many reported NiFe-based MOF and their derived materials show great potential for both HER and OER.^593−596^ However, most studies mainly focus on incorporating Ni and Fe metal cations into MOFs without rationally regulating the coordination sphere and the distance between the metal centers. Du and co-workers introduced a binuclear nickel complex into the MOF system.^236,237^ The resultant 2D layered MOF [Ni_2_ (PymS)_4_]*_n_* (PymSH = pyrimidine-2-thio) showed high catalytic activity for visible-light-driven hydrogen production under white LED light or even sunlight. The TOF of the catalyst can reach 10.6 h^–1^. Admittedly, the binuclear nickel complex may not be a close analogue of the active center in [NiFe] hydrogenase. To better mimic the [NiFe] center, Gennari and co-workers introduced a [NiFe]-hydrogenase model complex, [L^N2S2^Ni^II^Fe^II^Cp(CO)]BF_4_ into PCN-777.^238^ The cationic complex was encapsulated into the MOF cavity through noncovalent host–guest interactions,^597−599^ for which only a few precedents have been reported. Moreover, this method allowed them to achieve similar catalyst loading (∼30%, based on the NiFe: MOF linker ratio) in comparison to covalently attached [FeFe]–hydrogenase mimics (∼14–35% range), confirming this strategy’s loading efficiency. To date, examples of NiFe-MOFs mimicking the [NiFe]–hydrogenase are still rare, attributed to the smaller number and labile nature of the model compound resembling the [NiFe] active center. With the development of the postsynthetic modification methods of MOFs, more works on [NiFe]-based MOFs are expected in the future.

#### 3.6.3 Other MOFs

In addition, there are some unconventional hydrogenase-mimetic MOFs bearing other active centers. For example, Zeng and co-workers reported a 2D layered Mn-MOF [Mn_2_(TylP)_4_]*_n_* (TylP = 5-(1,2,4-triazol-1-yl) isophthalic) with a thiolate-bridged binuclear Mn(II) node similar to the active site of the [MnAu]–hydrogenase.^600^ This MOF showed a high catalytic activity for visible-light-driven hydrogen production under white LED light or sunlight, in which the turnover frequency can reach 6 h^–1^. In 2015, Du and co-workers prepared a 2D layered MOF [Ni_2_ (PymS)_4_]*_n_* with a thiolate-bridged binuclear Ni(II) node, mimicking the active site of the [NiFe] hydrogenases. This MOF possesses high catalytic activity for visible-light-driven hydrogen production under white LED or even sunlight with a turnover frequency (TOF) of 10.6 h^–1^. In addition, the [Ni_2_ (PymS)_4_]*_n_* shows high stability in aqueous solutions over a wide range of pH while its catalytic efficiency is still maintained.^601^ In 2017, Yuan and co-workers coupled a Ni(dmgH)_2_ complex with MIL-101(Cr) for photocatalytic H_2_ evolution under visible light irradiation. The optimal MIL-101(Cr)/Ni(dmgH)_2_ hybrid displays a H_2_ production rate of 45.5 μmol h^–1^, 10 times higher than the pristine MIL-101(Cr) sample.^602^ Hupp and co-workers also introduced MoS_*x*_ units to NU-1000 to partially mimic the metal–sulfur active site of hydrogenase.^603^ To overcome the MOF’s insulating nature that hindered high electrocatalytic performance, an archetypal redox mediator (RM), methyl viologen (MV_2_^+^), was added and resulted in more than 20-fold enhancement in the turnover frequency, implying efficient RM-assisted electron transfer to otherwise electrochemically silent MoS_*x*_ moieties.

In conclusion, the structure and function of hydrogenase may inspire researchers to solve environmental problems and develop efficient fuel cells. However, the utilization of hydrogenases still has many undeveloped concepts because most research only focuses on [FeFe] hydrogenase.^604^ It is intricated to design and synthesize MOFs reproducing the function of [FeFe] hydrogenase. A MOF with mesopores or macropores could be an ideal platform to immobilize the active site to ensure the substrate scope. Moreover, given the crystalline nature of MOFs, capturing some long-sought intermediates in hydrogenase mimicking MOFs could be promising, which may bring essential evidence for mechanistic studies. Although research of [NiFe] hydrogenase and [Fe] Hydrogenase is still scarce, more and more novel model complexes have been recently developed,^568^ including diiron(I) dithiolato carbonyl complexes and nickel hydride complexes,^605−607^ providing the other potential designs for photocatalytic MOFs.

### 3.7 Peroxidase-Inspired MOFs

Peroxidases or peroxide reductases represent a large group of enzymes and play a significant role in various chemical and biological processes. Peroxidases fueled the strong interests of researchers in the early days of enzymology, owing to the relative ease of preparing reasonable amounts of purified materials and observing the formation and decay of catalase intermediates.^608−610^ Most peroxidases only catalyze the hydrogen peroxide decomposition, while some are more active with specific organic hydroperoxides and enable the elimination of toxicity of hydrogen peroxide, phenols, amines, aldehydes, and benzene. In general, peroxidases can be divided into two categories: heme peroxidases and nonheme peroxidases. Heme peroxidases include catalases, dyp-type, nonanimal, animal, diheme cytochrome, and haloperoxidases, while nonheme peroxidases consist of haloperoxidases, alkylhydroperoxidases, thiol, NADH, and manganese peroxidases.

Some coordination complexes have been proved to efficiently catalyze the decomposition of hydrogen peroxide, of which porphyrin-based and salen-based complexes are the two most representative catalysts mimicking peroxidases. Porphyrin is the crucial part of hemoglobin, the functional part of peroxidases. Salens consist of four coordination donors to chelate different metal ions as active sites, displaying similar catalytic properties to porphyrin-based complexes. It should be noted that the chirality of salen-based complexes endows the catalyst with enantioselectivity.^5,6^ Some other complexes with appropriate structures have also been reported for H_2_O_2_ decomposition.^611^

#### 3.7.1 Porphyrinic MOFs

**Figure 37:**
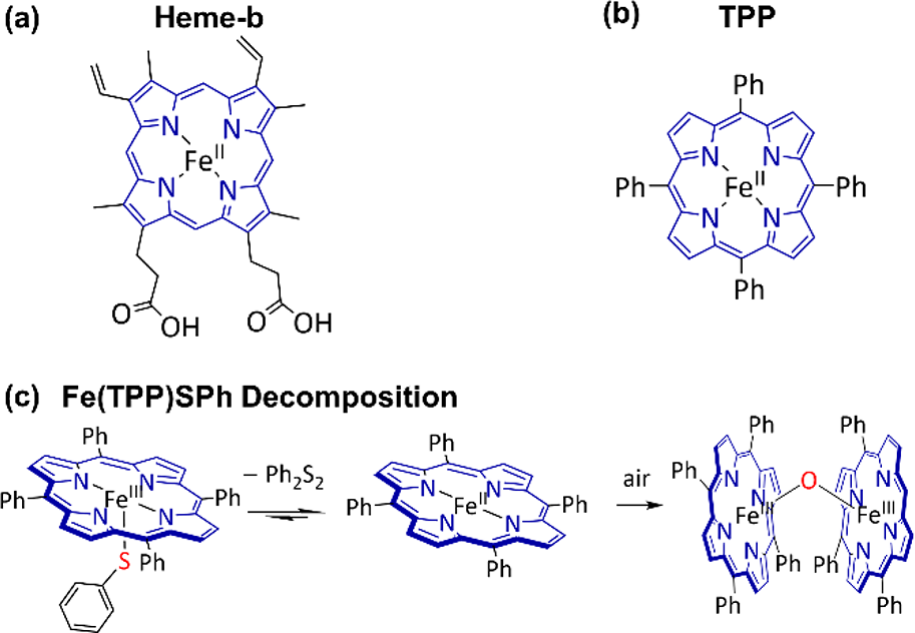
Structural illustration of (a) Heme-b and (b) TPP. (c) Decomposition of Fe(TPP) SPh to produce μ-oxo-bridged dimers.

Porphyrin complexes are well-known for their biological functions in aqueous media, such as light-harvesting, oxygen transportation, and catalysis.^612^ As the structural analogues of hemes,^613^ porphyrins were first oxidized to form highly active porphyrin radical intermediates in the catalytic oxidation cycle, which would insert the oxygen atom into organic substrates (Figure 37a). Synthetic metalloporphyrin model complexes provide simplified environments to study structure–function relationships, elucidate mechanisms, and identify reactive intermediates. Most heme model systems are metal–ligand complexes derived from iron tetraphenylporphyrin (FeTPP) (Figure 37b). However, the application of small molecule complexes has been limited by their oxidative instability. Without sufficient protection, Fe(TPP)-like complexes are prone to bimolecular decomposition into catalytically inactive μ-oxo porphyrin dimers (Figure 37c). The incorporation of axial thiolate ligands has been an even more challenging topic due to their tendency to form bis-axial coordination complexes and the sensitivity of the Fe–S bond to oxidation. These issues have been somewhat circumvented by electronic and steric tuning of the coordination sphere and strategically designed porphyrin ligands. Issues aside, the small molecule approach is fundamentally different from the native enzyme. Enzymatic heme is an immobilized cofactor embedded within a pocket created by the folding of the polypeptide backbone and is confined from the external environment and other active sites. Solid supports such as organic polymers, silica, and zeolites have been used to mimic the pore confinement effect, which has led to improved stability and catalytic efficiency, but their disordered structures preclude detailed elucidation and characterization of active species. Based on this, installing porphyrins into MOFs has been considered an efficient approach to avoid self-aggregation and prolong the lifetime of the catalysts.^257^ In addition, the crystalline nature of MOFs allows for characterizing the porphyrin structures through X-ray diffraction techniques.

Constructing highly efficient catalytic MOFs utilizing metalloporphyrin has fueled the considerable interest of researchers over the past few years. In 1994, Robson and co-workers succeeded in creating a crystalline framework with permanent micropores by utilizing copper porphyrins as building blocks. This work emphasized the catalytic potential of porphyrinic MOFs.^614^ In 2002, Suslick and co-workers reported a functional microporous material based on the supramolecular assembly of carboxylate-substituted porphyrins with cobalt ions.^615^ In 2009, Hupp and Nguyen reported that ZnPO-MOF can catalyze acyl-transfer reactions and preconcentrate substrates within its pores.^616^ Later, they modified the porphyrin center of ZnPO-MOF with Al^3+^, Zn^2+^, Pd^2+^, Fe^3+^, and Mn^3+^ to be competent for the oxidation of alkenes and alkanes.^617^ In 2012, Ma and co-workers constructed a highly stable mesoporous porphyrinic MOF MMPF-6 with 1.1 and 3.3 nm 1D open channels. MMPF-6 demonstrated interesting peroxidase activity comparable to that of myoglobin as well as exhibited solvent adaptability during the catalysis.^618^ Rosseinsky and co-workers used free-base *meso*-tetra (4-carboxyl-phenyl) porphyrin and AlCl_3_·6H_2_O to obtain a highly stable and porous Al-PMOF. The visible-light photocatalytic activity of this porphyrin-based material is shown for the sacrificial hydrogen evolution from water.^619^

**Figure 38:**
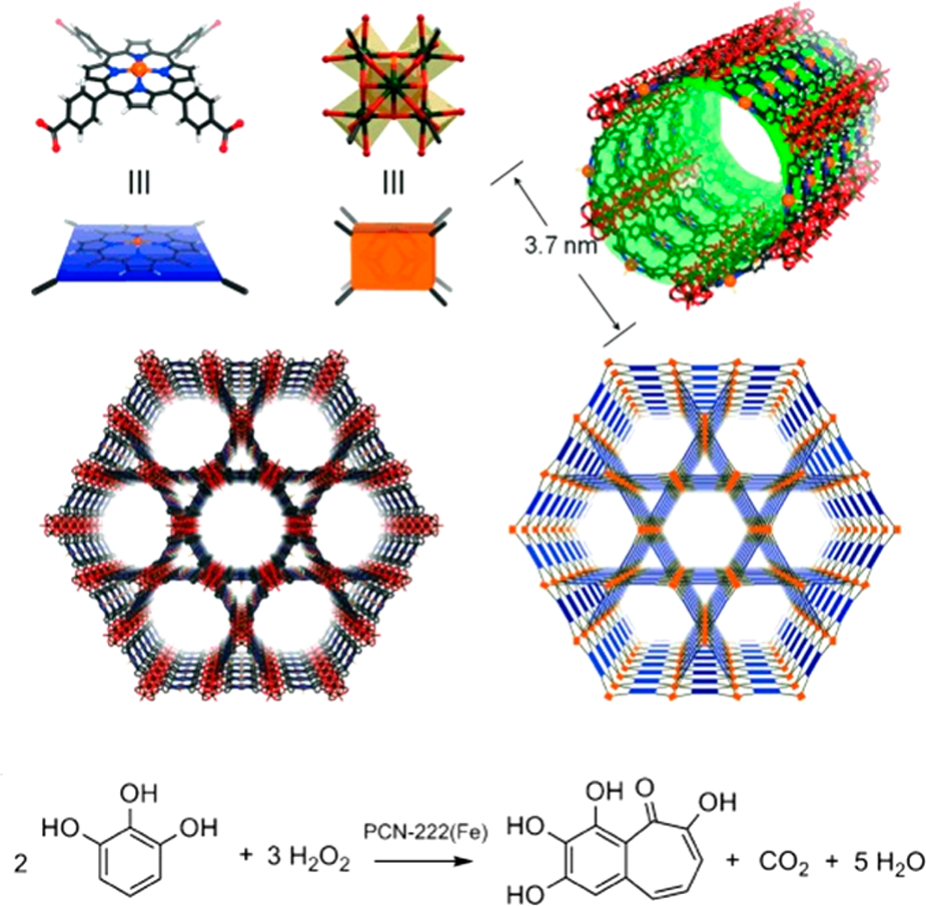
Crystal structure of PCN-222(Fe) with a **csq** network topology, enabling oxidizing pyrogallol by hydrogen peroxide. Reproduced with permission from ref (64). Copyright 2012 John Wiley and Sons.

In 2012, the Zhou group combined Fe-TCPP and highly stable Zr_6_ clusters to construct a biomimetic porphyrin MOF, PCN-222(Fe), with 3.7 nm 1D open channels.^64^ PCN-222(Fe) is stable even in concentrated hydrochloric acid for more than 24 h without changes in crystallinity and porosity. Furthermore, PCN-222(Fe) shows peroxidase-like catalytic activity and broad substrate scope in an aqueous solution. Remarkably, PCN-222(Fe) features an excellent catalytic activity (*k*_cat_  = 16.1  min^–1^) for pyrogallol, superior to molecular hemin (*k*_cat_  = 2.4  min^–1^) in aqueous media. The catalytic center of PCN-222 is decorated on the wall of the open channel. The integration of dense catalytic centers, ultralarge open channels, and the extraordinary chemical stability of PCN-222(Fe) sheds light on building MOF-based enzyme-mimic catalysts (Figure 38).

**Figure 39:**
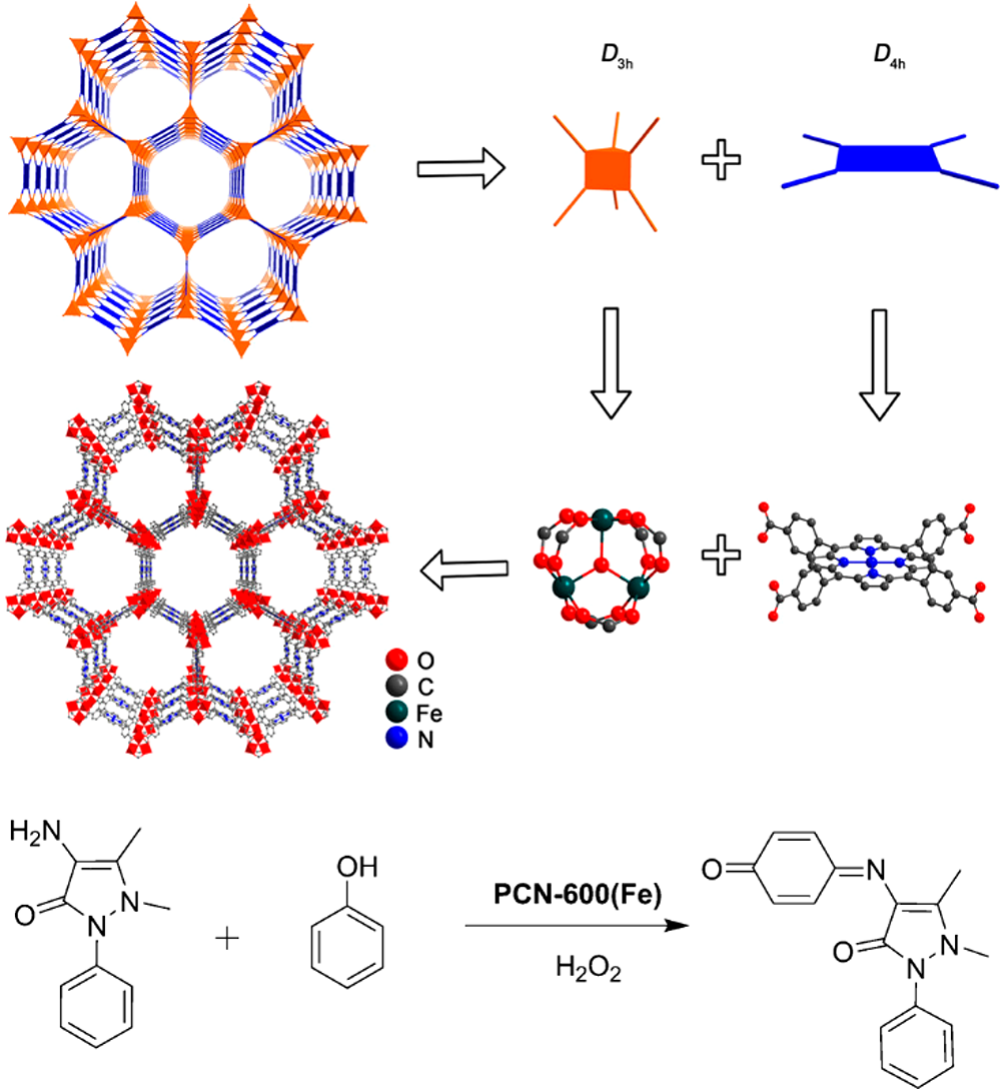
Crystal structure and building blocks of PCN-600 for catalytic oxidation. Reproduced with permission from ref (65). Copyright 2014 American Chemical Society.

In 2014, Zhou group constructed a series of mesoporous metalloporphyrin MOFs, PCN-600(M) (M = Mn, Fe, Co, Ni, Cu) using a preassembled [Fe_3_O(OOCCH_3_)_6_] cluster.^65^ PCN-600(M) exhibits 3.1 nm 1D channels and remains stable in aqueous solutions with pH values ranging from 2 to 11. During the catalytic oxidation, PCN-600(Fe) has a much smaller Michaelis–Menten constant (*K*_m_) (*K*_m_ = 6.37 mM) than wild-type cytochrome c from bovine hearts (*K*_m_ = 89.4 mM), indicating that PCN-600(Fe) has a higher affinity for substrates (Figure 39).

In 2015, Cui and co-workers encapsulated Hemin into a Cu-MOF HKUST-1 to construct Hemin@HKUST-1 composites.^62^ The synthesized Hemin-functionalized MOF exhibits an excellent catalyst activity and can be cyclically utilized as solid peroxidase-mimic in the neutral condition. Furthermore, the obtained Hemin@HKUST-1 composites have been used to develop practical sensors to detect H_2_O_2_ and glucose with a wide response range and a low limit of detection (LOD), based on the catalysis efforts.

Based on the above discussions, porphyrinic MOFs, as peroxidase mimics, possess great application potentials to catalyze hydrogen peroxide decomposition. Apart from that, Natale and co-workers have summarized chemical sensor applications of porphyrin-based complexes in 2017.^284^ Xie and co-workers have reviewed the recent development of ion chemosensors based on porphyrin-based complexes in 2017.^620^ Dong and co-workers have summed up the synthesis and applications of rhodium porphyrin complexes in 2018.^621^ Kaskel and co-workers have summarized porphyrin-based metal–organic frameworks for biomedical applications in 2021.^622^ Cao and co-workers have listed the porphyrin-based frameworks for oxygen electrocatalysis and catalytic reduction of carbon dioxide in 2021.^250^ All of these highlights the catalytic potential of porphyrinic MOFs.

In biomimetic catalysis, active sites can be installed into MOFs. For example, cytochrome P450, consisting of a large family of cysteinato-heme enzymes, is capable of catalyzing various oxidative transformations in multiple organisms.^623^ The source of its catalytic activity, functionalized metalloporphyrins, has been assembled into the MOFs. The Zhou group prepared stable Zr-MOFs with a high density of metalloporphyrinic centers. PCN-222 series were designed and synthesized with metalated tetrakis (4-carboxyphenyl) porphyrin (M-TCPP) linkers. PCN-222(Fe) built from Fe-TCPP linkers exhibited excellent peroxidase catalytic activity.^64^ The oxidation of pyrogallol was catalyzed by PCN-222(Fe), in which enzyme-mimetic kinetics were observed according to the linear Lineweaver–Burk plot of the variable oxidation rates and substrate concentrations. PCN-222 series are reckoned as an ideal platform for mimicking cytochrome P450 enzymes due to their stability, mesoporosity, and high density of metalloporphyrin centers. Another example is hemoglobin-like MOFs, which can selectively bind with the O_2_ in the air. The solid-state material is beneficial to the industrial separation of O_2_ from the air. The Long group employed unsaturated copper(II) and iron(II)-based MOFs to investigate the O_2_-binding activities.^624^ The O_2_ uptake for the Cr_3_ (btc)_2_ MOF rose sharply to 11 wt %, while the N_2_ uptake turned out to be 0.58 wt %. A remarkable O_2_/N_2_ selectivity factor of 22 was achieved. However, further research is needed to explore its catalytic activities based on the O_2_ affinity.

#### 3.7.2 Salen MOFs

Salen is the abbreviation of salicylaldehyde and ethylenediamine, representing a common chelating ligand in coordination chemistry and homogeneous catalysis. It is generally chiral and soluble in polar organic solvents. Salens consist of four coordination donors that can chelate different metal ions as active sites and display similar catalytic properties to porphyrin-based complexes.^625^ In the past few years, chiral salen complexes have played a crucial role as the functional ligands in MOF-based catalysts, especially asymmetric catalysts, due to their unique structural features and catalytic activity.

Although plenty of MOFs have been applied as heterogeneous catalysts, most of them only enable simple organic transformations without stereoselectivity. Based on their inherent chirality, salen MOFs have been widely applied as asymmetric catalysts. In 2006, Hupp and co-workers reported a microporous Mn-salen-based MOF as an effective asymmetric catalyst for olefin epoxidation, with a 71% yield and an 82% ee value.^626^ In 2011, Lin and co-workers synthesized CMOF-1 with chiral Mn-salen ligand, and used it in highly regio- and stereoselective sequential alkene epoxidation and ring-opening reactions, which is the first MOF-catalyzed sequential asymmetric reaction.^627^ In 2011, Lin and co-workers constructed a pair of interpenetrated and noninterpenetrated CMOF-1 and CMOF-2 with Ru-salen units, which exhibited remarkable catenation-dependent catalytic activity; noninterpenetrated R-CMOF-2 was highly active, whereas interpenetrated R-CMOF-1 was nearly inactive because of its inability to transport the substrates through its small channels.^628^ In 2014, Cui and co-workers reported two chiral porous Fe-salen-MOFs, which featured efficiency and enantioselectivity in catalyzing the oxidation of sulfides to sulfoxides, comparable to the homogeneous catalysts.^629^

**Figure 40:**
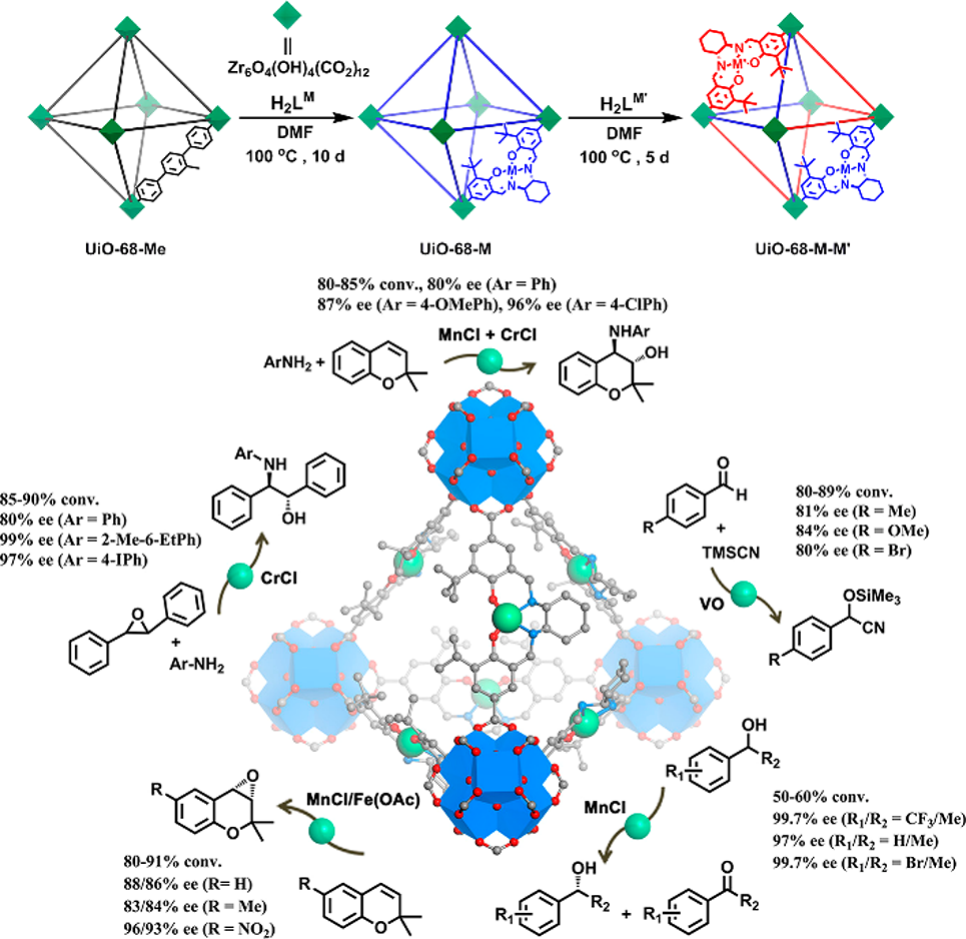
Preparation of mixed-metal-salen MOFs through postsynthetic linker exchange. The salen-based MOFs enable conducting multiple asymmetric catalysis efficiently. Reproduced with permission from ref (630). Copyright 2018 American Chemical Society.

In 2018, Cui and co-workers capitalized on a postsynthetic modification to exchange the achiral linkers in Zr-based UiO-68 with chiral metal-salen (M = Cu, Fe, Cr, V, and Mn) linkers, to obtain UiO-68-Me.^630^ Even further, they modified the MOF UiO-68–Mn by ligand exchange to get mixed-metal-salen MOFs UiO-68–Mn-Cr and UiO-68–Mn-V (Figure 40). The two MOFs feature distinct catalytic performances: the single-metal-salen MOFs are active catalysts for asymmetric cyanosilylation of aldehydes, ring-opening of epoxides, oxidative kinetic resolution of secondary alcohols, and aminolysis of stilbene oxide, while the mixed-metal-salen MOFs catalyze sequential asymmetric alkene epoxidation and epoxide ring-opening reactions. All of the chiral MOFs are highly enantioselective, heterogeneous, and recyclable, confirming that the postsynthetic modification was a feasible and efficient approach to fabricating MOF-based chiral catalysts.

**Figure 41:**
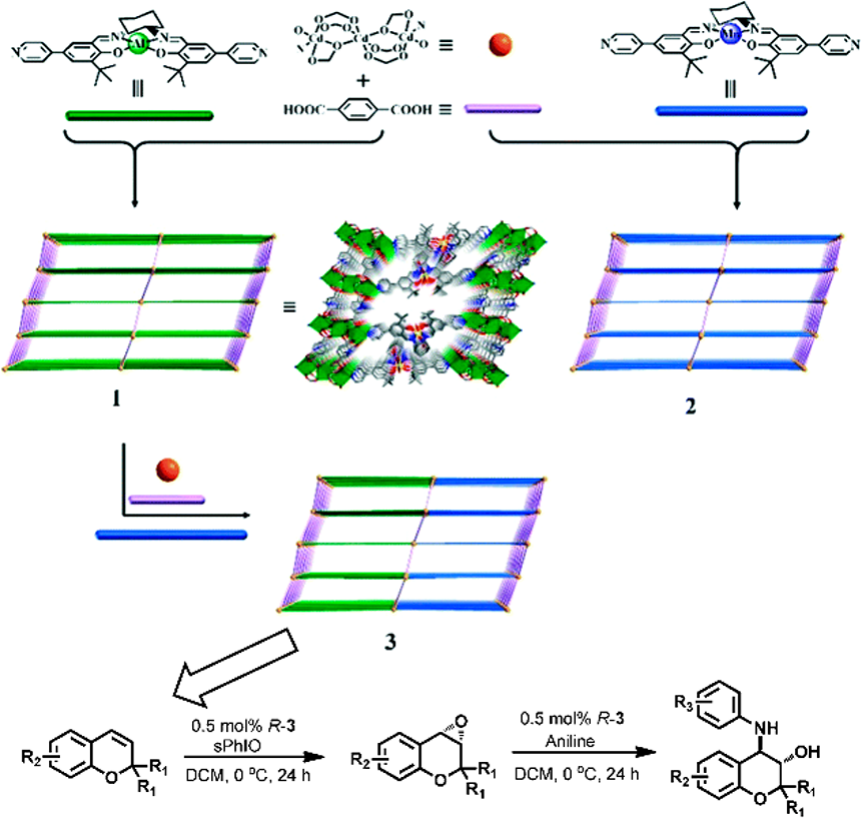
Constructing chiral MOF@MOF composites from M-salen ligands for asymmetric epoxidation/ring-opening reactions. Reproduced with permission from ref (631). Copyright 2019 Royal Society of Chemistry.

In 2018, Cui and co-workers successfully constructed two chiral metal-salan frameworks MOF-1 and MOF-2, using dipyridylfunctionalized Al–salen and Mn–salen ligands.^631^ Apart from that, a heterostructure named MOF-3 could be constructed by encapsulating MOF-1 into MOF-2. All of the MOFs possess excellent catalysis functions. Notably, the composite MOF featuring distinct M-salen sites are efficient and recyclable heterogeneous catalysts for asymmetric sequential alkene epoxidation and epoxide ring-opening reaction, with good reactivity and stereoselectivity beyond homogeneous catalysts. The successful preparation of the composite MOF provides a new strategy for constructing efficient and multifunctional heterogeneous catalysts (Figure 41).

Based on the above discussions, salen MOFs are promising enantioselective catalysts for some typical oxidation reactions. Apart from that, Jacobsen summarized the privileged chiral catalysts, mainly including salen and cinchona, and their properties in enzymatic reactions in 2003.^251^ Cozzi discussed the practical guidelines for the preparation and use of different metal–salen complexes in the field of catalytic transformations in 2004.^625^ Kleij focused on the π-conjugated salen systems and their interesting photophysical and supramolecular properties in 2012.^252^ Correia concluded the advantages and disadvantages of metal–salen complexes in catalysis and medicinal applications in 2019.^632^ White primarily summarized the developments in chiral metal–salen catalysis with particular emphasis on those applications of importance in asymmetric synthesis in 2019.^633^ All of these exhibit the extensive applications of salen-based complexes as chiral catalysts.

#### 3.7.3 Other MOFs

Except porphyrinic and salen MOFs, some other MOFs were also confirmed as peroxidase-mimicking catalysts, especially Fe-MOFs, Cu-MOFs, and mixed-metal MOFs. In 2013, Jiang and co-workers reported that MIL-53(Fe) possessed intrinsic peroxidase-like activity for catalyzing the oxidation of 3,3′,5,5′-tetramethylbenzidine and *o*-phenylenediamine in the presence of H_2_O_2_, in which the ascorbic acid showed inhibition effect on the oxidation of *o*-phenylenediamine.^611^ In 2020, Li and co-workers synthesized a Fe-loaded MOF-545 (Fe) and utilized its peroxidase-like activity to remove dyes.^634^ In 2022, Meng and co-workers reported a hybrid material based on gold nanorods and a Fe-MOF, which showed excellent stability and reproducibility for photoenhanced peroxidase-like catalysis.^635^

In 2017, Tan and co-workers reported that Cu-MOF nanoparticles with an average diameter of 550 nm enabled catalyzing the yellow chromogenic reaction of 3,3′,5,5′-tetramethylbenzidine in the presence of H_2_O_2_.^636^ In 2019, Wang and co-workers synthesized a stable [Cu(PDA)(DMF)] under solvothermal conditions, which can catalytically oxidize the colorless substrate 3,3′,5,5′-tetramethylbenzidine to a blue product in the presence of H_2_O_2_.^637^ In 2020, Liu and co-workers designed and synthesized a heteropoly acid-encapsulated Cu-MOF with metal–carbene structure, which can act as a bifunctional enzyme-mimetic catalyst for colorimetric detection of H_2_O_2_ and ascorbic acid.^638^

**Figure 42:**
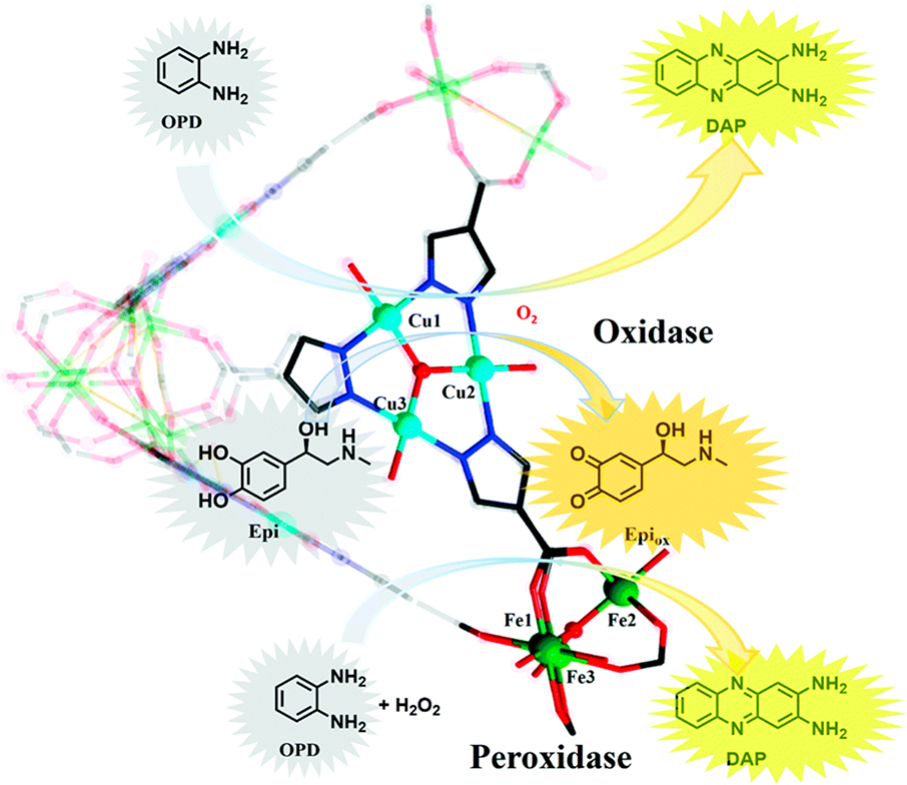
Illustration of bimetallic MOF-919 (Fe–Cu) mimicking bifunctional oxidase–peroxidase catalytic activity. Reproduced with permission from ref (640). Copyright 2022 Royal Society of Chemistry.

In 2019, Zhao and co-workers synthesized a bimetallic Co/Mn-MOF via a one-step hydrothermal reaction. Based on Co/Mn-MOF’s excellent peroxidase-like activity, a colorimetric sensor for detecting H_2_O_2_ was successfully fabricated.^639^ In 2022, Yeh and co-workers constructed a bimetallic MOF-919(Fe–Cu) nanozyme with the ability of bifunctional enzyme-mimicking catalysis^640^ (Figure 42). In 2022, Wang and co-workers developed bimetallic Fe_*x*_Ni_*y*_-MOFs with enhanced peroxidase-like activity, owing to the improved redox capacity and accelerated electron transfer between 3,3′,5,5′-tetramethylbenzidine and H_2_O_2_.^641^

In summary, in peroxidase-mimicking catalysis, MOFs benefit from their high density of active sites, predesigned reaction pockets, and hierarchical structures, indicating great potentials for heterogeneous catalysis. In addition, the combinations of highly active metal centers and functional ligands can synergistically improve the catalytic activities.

## 4 MOFS as Enzyme Mimics

**Figure 43:**
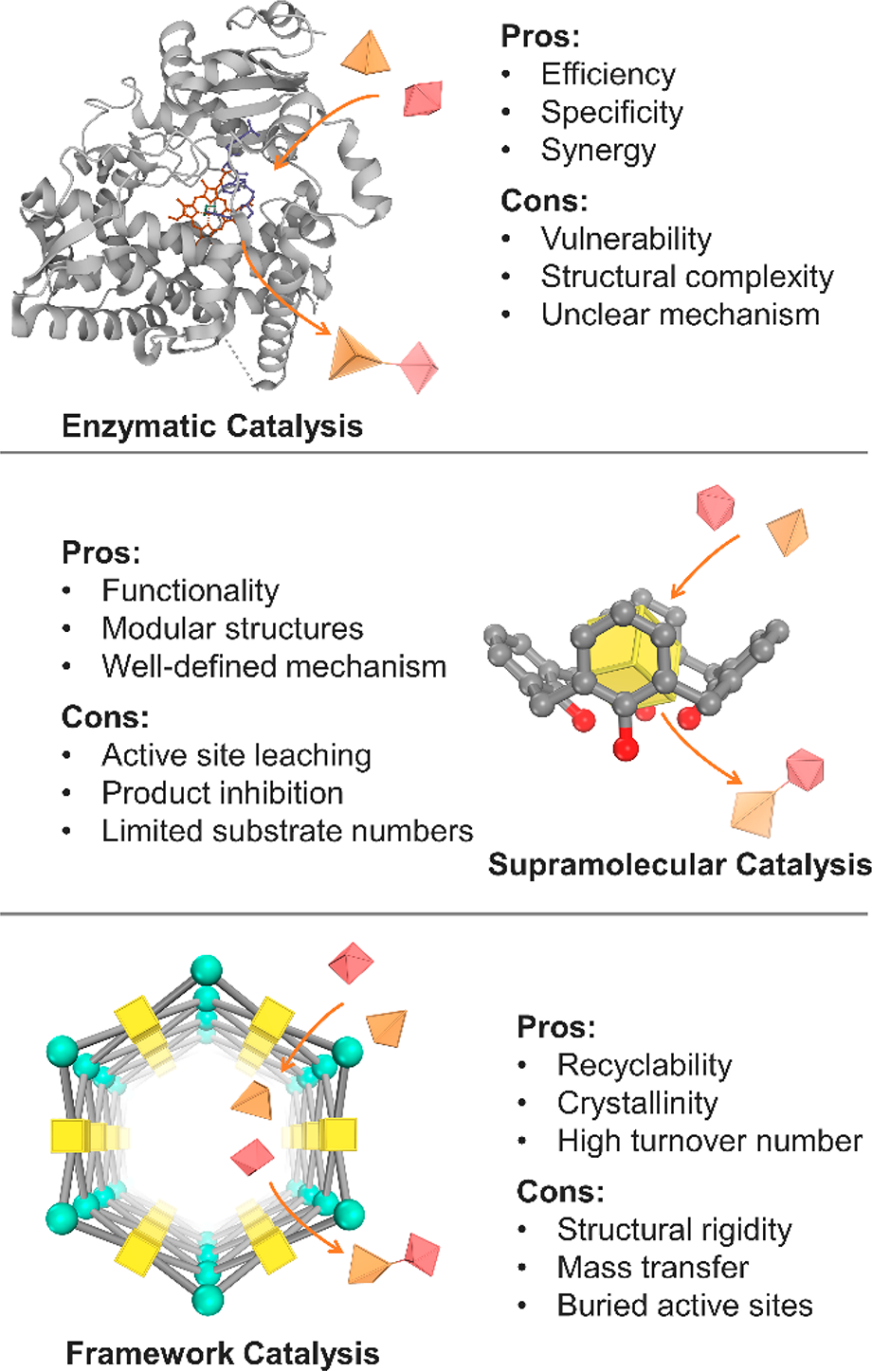
Comparison between enzymatic catalysis, supramolecular catalysis, and framework catalysis.

Enzymes are highly efficient and specific biocatalysts that can work in synergy to catalyze sophisticated reactions and produce complex compounds. Yet, the applications of enzymes are largely hindered by their complex and vulnerable structures. In addition, mechanical studies are also intricated in enzymatic catalysis (Figure 43). To borrow rate acceleration and chem-/stereoselectivity of enzymes, supramolecular catalysts have been developed by introducing active sites into supramolecules, in which the host–guest interactions play significant roles in capturing reagents and stabilizing transition states.^642^ Owing to their modular structures, supramolecular catalysts feature diversity in geometries and function groups. Detailed mechanistic studies are plausible through multiple spectroscopic techniques. Nevertheless, supramolecular catalysts usually suffer from active site leaching and product inhibition. Their small cavities may also limit the intermolecular reaction involved with multiple substrates (Figure 43). As a new class of supramolecular catalysts with periodically aligned cavities, MOFs inherit the functionality and modularity of supramolecules. In addition, MOFs possess unique merits in catalysis, such as high recyclability and large turnover number, because the frameworks can prevent the aggregation and leaching of active sites. MOFs’ crystallinity ensures structural characterization using X-ray diffraction techniques. Admittedly, the rigid skeletons of MOFs may lead to mismatch between the active sites and transition states, deteriorating the catalytic selectivity. Besides, most reported MOFs have micropores less than 2 nm, which lead to insufficient mass transfer and inaccessible active sites (Figure 43). These factors place obstacles in developing MOF-based enzyme mimics, which may be overcome by constructing flexible and hierarchically porous MOFs to emulate enzymes. In this section, we will discuss how the nanopores are regulated to endow MOFs with enzymelike catalytic activity.

### 4.1 Confinement Effect

**Figure 44:**
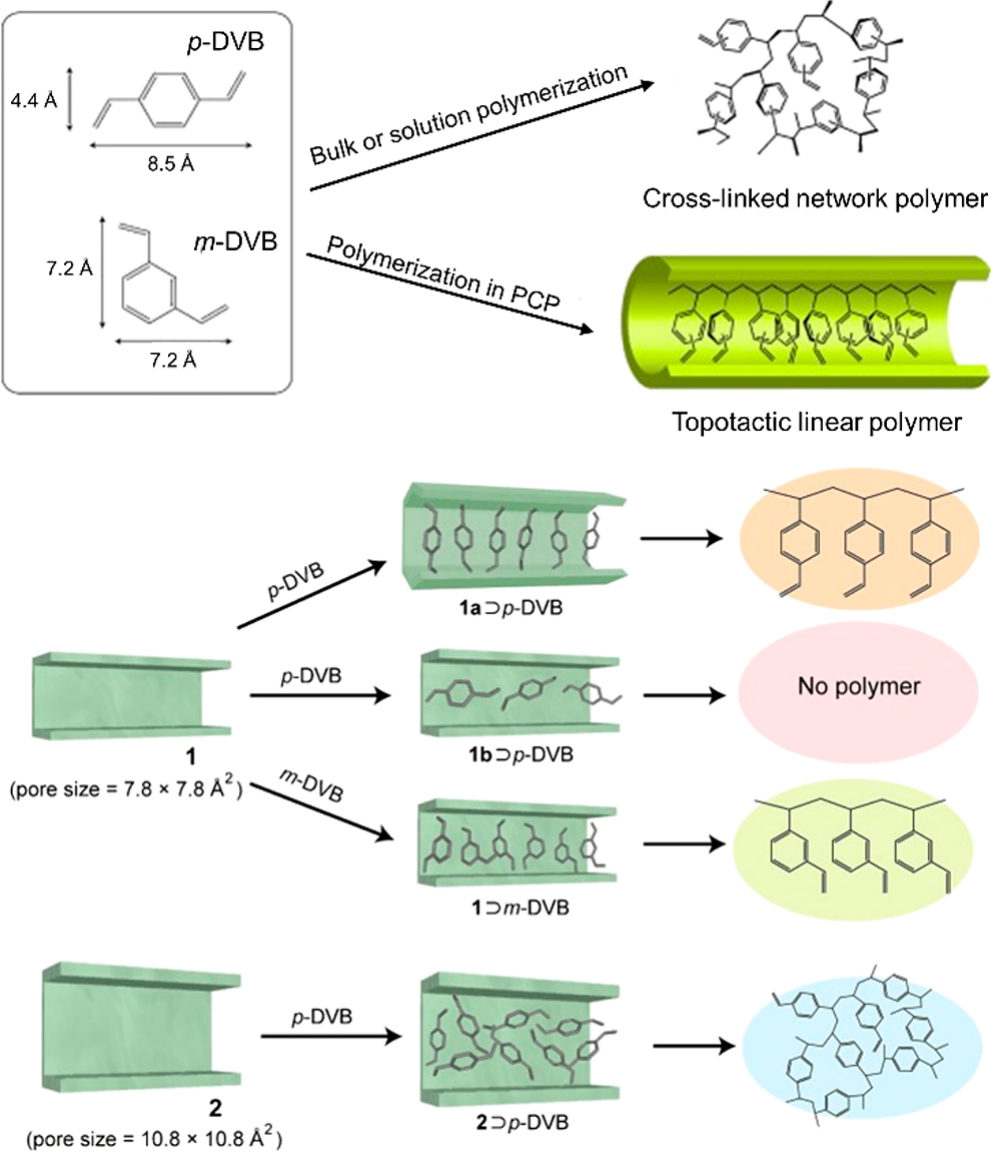
Polymerization within MOFs with different channel sizes resulting in diverse products. Reproduced with permission from ref (645). Copyright 2007 John Wiley and Sons.

MOFs are known to exhibit dynamic features and confinement effects to guest molecules. The confinement can be exerted upon substrate molecules and catalysts. MOFs’ adsorption capability toward substrates can be altered by the rotation of organic linkers. The substrate molecules show covalent/noncovalent interactions with MOF pores. MOFs that provide covalent interactions are generally termed as “nanoreactors”, while MOFs that noncovalently interact with molecules are termed as “nanovessels”. MOF pores and guest molecules mutually interact with each other during catalysis. For instance, Matsuda and co-workers reported shape-responsiveness of a copper-based metal–organic framework CPL-2 toward the guest molecule benzene.^643^ The coordination sphere of copper changes from square planar to square pyramidal upon binding with benzene molecules. Later Matsuda et al. and Kubota et al. reported other copper-based MOFs, named [Cu_2_(pzdc)_2_(bpy)]^644^ and CPL-1 (Figure 44).^645^ Upon binding with acetylene, CPL-1’s pores undergo a phase transfer process to contain the guest acetylene molecules. This feature has been applied in the controlled polymerization of substituted acetylenes by Uemura and co-workers. A pillared-layer microporous compound with one-dimensional channels, [Cu_2_(pzdc)_2_(pyrazine)]*_n_*, was reported to exhibit electron-sufficient oxygens in the pores which can specifically adsorb acetylene molecules. The adsorption is enhanced with the acidity of the acetylene’s protons. Thus, methyl propiolate (MP), a monosubstituted acetylene derivative, was studied as the monomer. PolyMP was obtained and characterized with a molecular weight controlled by the nanochannel.^646^

**Figure 45:**
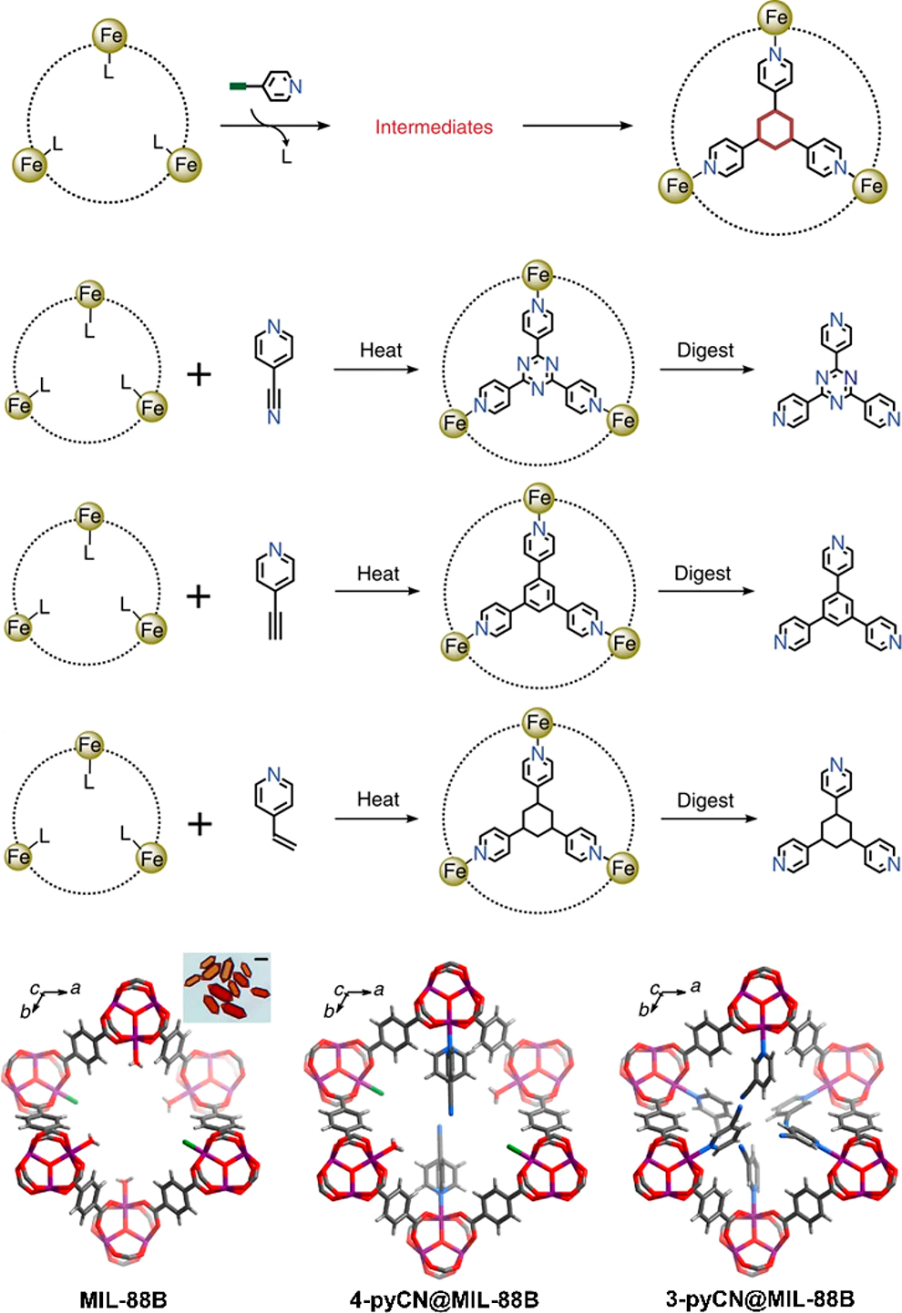
Fixation and [2+2+2] cyclotrimerization of substituted pyridines bearing unsaturated functional groups within MIL-88B (Fe). The locations of pyridine monomers are confirmed by SCXRD. Reproduced with permission from ref (647). Copyright 2015 Springer Nature.

In another case reported by Zhang and co-workers, 4-cyanopyridine, 4-ethynylpyridine, and 4-vinylpyridine were selected as the monomers in MIL-88B (Fe) catalyzed [2+2+2] cyclotrimerization^647^ (Figure 45). Revealed by SCXRD data, the pyridines serve as electron donors and anchor the monomers to the open metal sites of Fe_3_ clusters, while the unsaturated groups point to the center of MOF channels. Upon heating, the trimerization takes place with geometric selectivity, which is impossible by conventional pathways.

**Figure 46:**
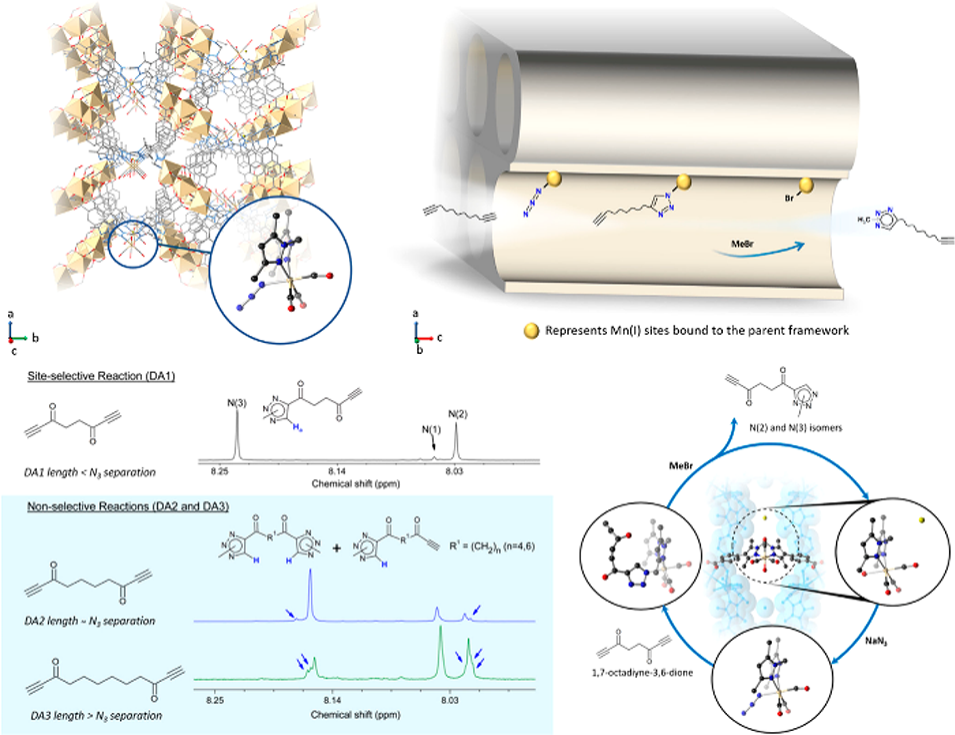
(top) Illustration of the azide sites and the site-selective click reaction of a dialkyne within a Mn-based MOF. Bottom: The click reaction products of dialkynes with varied chain length. The site-selective click reaction involves the immobilization of azides and regeneration via alkylation with MeBr. Reproduced with permission from ref (648). Copyright 2018 American Chemical Society.

In 2018, Doonan, Sumby, and co-workers reported the site-selective click reaction within MOFs, providing another example of MOFs as “nanovessels”^648^ (Figure 46). Initially, a Mn(II)-based MOF was metalated with [Mn(CO)_5_Br], and azide anions were immobilized within the one-dimensional channel of the MOF, anchoring to the Mn(I) sites. As a result, the azide-embedded MOF can conduct site-selective [3+2] azide–alkyne cycloaddition of a symmetrical dialkyne named 1,7-octadiyne-3,6-dione, generating a mono-“click” product and trace bis-triazole as the side-product. When extending the length of the dialkyne, the selectivity will be lost, implying that the spatial isolation of azides plays a critical role in the site-selective click reaction. This work indicates that MOFs with elaborately tailored pore environments can serve as the physical protecting groups for site-selective reactions.

The Zhou group developed a series of core–shell MOF PCN-222@Zr-BPDC, PCN-222@Zr-NDC, and PCN-222@Zr-AZDC. It is worth noting that Zr-BPDC, Zr-NDC, and Zr-AZDC are the alternative denotations of the UiO-series. PCN-222(Fe)@Zr-BPDC proved high conversion in the catalytic epoxidation of alkenes.^649^ Small olefins exhibit higher conversion compared to bulkier olefins. The difference in conversion was explained by the shielding of the shell MOFs. The presence of shell MOFs limits the diffusion rates of the substrates and reduces the accessibility toward the catalytic centers, enhancing the size exclusion effect of MOF catalysts.

There are other examples of MOFs as “nanovessels”. Controlled linear polymerization of divinylbenzenes (DVBs) was achieved inside the nanochannels of [M_2_(1,4-bdc)_2_(TED)] (bdc = benzenedicarboxylate; TED = triethylenediamine; M = Zn^2+^, Cu^2+^).^650^ The discovered topotactic selectivity is attributed to the consequential confinement of monomers inside the micropores. Conversely, MOFs with larger pores resulted in nonselective polymerization and cross-linked polymer networks. The strength of this method is that it ensures the trans-selectivity in chain propagation and avoids trimolecular cyclization. So far, a group of conventionally unavailable chemical transformations have been achieved by the using MOFs as nanoreactors or nanovessels. However, few examples cover the recyclability of MOFs, suggesting the stability of catalysts under reaction conditions needs to be improved.

**Figure 47:**
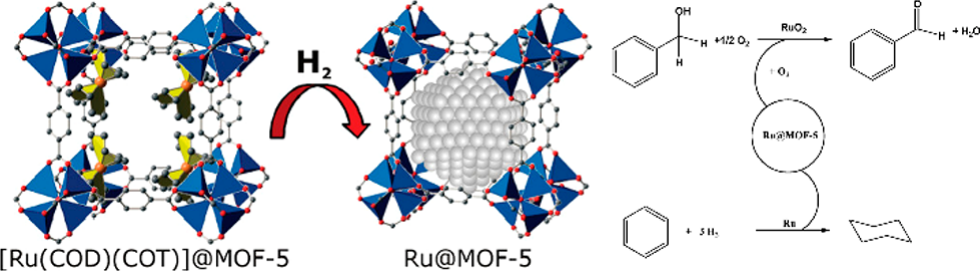
Quantitative hydrogenolysis to generate catalytic Ru@MOF-5 composites. Reproduced with permission from ref (651). Copyright 2005 John Wiley and Sons.

The high surface area of the MOF pores provides sufficient confinement for a monodisperse loading of catalytically active species. For example, nanosized metal particles, Pt, Au, Pd, and Ru, were reported to be successfully encapsulated inside the pores of MOF-5 and MOF-177 (Figure 47).^651,652^ Remarkably, owing to the framework confinement, the metal nanoparticles usually featured a defined size that was smaller or matched the MOF pore size. The MOF-5-encapsulated Pd, reported by Opelt and co-workers, was utilized for the catalytic hydrogenation of alkenes, showing twice high activity as that of a commercial Pd/C catalyst.^653^ The Opelt group synthesized another Pd-based MOF, [Pd(2-pymo)_2_]*_n_* (2-pymo = 2-pyrimidinolate), which showed high selectivity in hydrogenation of 1-octene and cyclododecene.^654^ The bulkier cyclododecene being excluded by the pores of [Pd(2-pymo)_2_] led to the shape- and size-selectivity toward 1-octene in hydrogenation. In addition, strong adsorption of substrates plays a significant role in MOF-based catalysis, endowing the catalyst with enhanced activity and selectivity. For instance, Sadakiyo and co-workers systematically studied the substrate adsorption strength of seven different MOFs.^655^ The results indicate that MOFs bearing amino groups feature improved affinity for acetic acid, accounting for their high reactivity in acetic acid hydrogenation.

In a confined space,^656,657^ catalysts can be localized in specific pores. Pore size can be tailored to fit target guests and pore environment can be modified to bind with them chemically/physically. Guests include metal atoms,^653,658−661^ clusters,^657^ oxides,^662^ and nanosheets (metal halide sheets).^656^ The Deng group loaded TiO_2_, mainly in the form of anatase, into the mesopores I and II (pore sizes of 29 Å, 34 Å, respectively) of MIL-101 while preserving the crystallinity of the MOF. Various loading ratios were achieved, ranging from 13% to 47%, as detected by a 3D electron-density map and were concurrently visualized by scanning tunneling electron microscope (STEM). The catalytic activity of TiO_2_ in MIL-101(Cr) was tested in CO_2_ photoreduction, where a significant enhancement in the turnover frequency (TOF) was observed: 5.9 for TiO_2_ units in compartment II, 0.13 for TiO_2_ units in compartment I, and in contrast, 3.4 × 10^–3^ for MOF-surface located TiO_2_, 1.7 × 10^–4^ for sole TiO_2_. Interestingly, X-ray photoelectron spectroscopy (XPS) and X-ray absorption spectroscopy (XAS) revealed the participation of Cr-based SBUs as the catalytic sites for CO_2_ photoreduction, which is rarely reported by previous research. Additionally, the inappropriate bandgap overlaps between TiO_2_ and the MOFs make the electron transfer unable be illustrated by traditional heterojunction theory, where MOFs are treated as semiconductors. These results suggest more complicated interactions between the loaded anatase TiO_2_ and MOFs, demonstrating this strategy’s potential to overcome the bandgap limitation of traditional semiconductors.

### 4.2 Lewis Acid/Base Sites

Captured in the pores of MOFs, substrates have a significantly higher collision probability with the active sites within the frameworks. Thus, the rational design of pore environment takes a predominating role in tuning MOFs’ catalytic activity. Tunable metal clusters and functionalized linkers of MOFs allow pore environment alteration. Oxos in the clusters, functional groups in the linkers, can be proton acceptors and exhibit Brønsted-basic properties. Confinement effect operates in MOFs with Brønsted-basic properties to construct an elevated concentration of active sites. Hartmann and co-workers synthesized three basic MOFs: NH_2_-MIL-101(Fe), NH_2_-MIL-101(Al), and CAU-1, where amine groups are introduced by amine-functionalized terephthalic acid.^663^ Noncoordinated primary amines can participate in the Brønsted-basic catalysis, without the assistance of polar protic solvents. The Knoevenagel condensation of benzaldehyde and ethyl cyanoacetate was selected to study the catalytic capability of the amino-functionalized MOFs. NH_2_-MIL-101(Fe) and NH_2_-MIL-101(Al) exhibited excellent catalytic activity with ∼90% yield for the Knoevenagel condensation reaction, while CAU-1 proved a poor catalyst because small windows of the framework hinder the mass transfer.

The electronic modification of the pore environment can also tune MOFs’ catalytic performance. The Speybroeck and De Vos group studied the influence of linker substitution upon the catalytic properties of UiO-66.^664^ The cyclization of (+)-citronellal was selected as the model reaction. Unsaturated zirconium clusters provide active Lewis-acidic sites. With electron-withdrawing groups installed, there was an enhancement in the Lewis acidity of the Zr ions. Therefore, a positive correlation between the log *k* (reaction rate) and σ_*m*_ (inductivity factors of the functional groups) was discovered. The nitro-group substituted UiO-66 was confirmed as the most active material. Interestingly, the results provided a linear free-energy relationship (Hammett-type LFER) between the degree of substituents’ electron-withdrawing character and the carbonyl-ene reaction’s rate, which was the first LFER observed in the MOF-based catalysis. They also utilized simulation to calculate the transition state of the UiO-66···citronellal complex. UiO-66-NO_2_ provides nitro groups with outstanding Lewis acidity to assist the proton transfer of the substrate. The transition-state energy of UiO-66-NO_2_···citronellal is 19.0 kJ mol^–1^ lower than UiO-66···citronellal, illustrating the faster reaction rate.

MOFs can be multifunctional. Amine, carboxylate, halides, and many other functional groups can be installed by prefunctionalized linkers and postsynthetic functionalization. In specific applications, a confined space is combined with an acid and/or base moieties. The Duan group designed and synthesized a terbium-clustered MOF named Tb-TCA (H_3_TCA = tricarboxytriphenylamine).^665^ The Tb^3+^ ions showed Lewis-acidity, while the triphenylamines acted as the Lewis-base. The MOF features a tetragon nanochannel with a size of 7.5 Å × 8.5 Å. The pores can accommodate salicylaldehyde molecules, leading to a high conversion >90% in the Knoevenagel condensation reaction with cyanotrimethylsilane. In previous reports by the Ahn group, Co-MOF-74 containing cobalt open metal sites with strong Lewis-acidity performed high CO_2_ adsorption and high catalytic yield (96%) in the cycloaddition of CO_2_ to styrene epoxide.^666^

In 2020, the Lin group reported the orthogonal incorporation of Lewis acid and palladium nanoparticles (NPs) in an Al MOF bearing 2,2′-bipyridine-5,5′-dicarboxylate (dcbpy) and 1,4-benzenediacrylate (pdac) ligands.^667^ The Al_2_ (OH)(OH_2_) sites can be modified by using trimethylsilyl triflate to afford strong Lewis-acidic sites for dehydroalkoxylation. Subsequently, Pd(MeCN)_2_Cl_2_ can coordinate to dcbpy ligands, followed by in situ reduction to provide Pd nanoparticles. This tandem catalytic system enables dehydroalkoxylation–hydrogenation of etheric, alcoholic, and esteric C–O bonds to generate saturated alkanes under relatively mild conditions.

Recently, there are reports on simultaneous incorporation of Lewis acid and base into MOFs to afford frustrated Lewis pair (FLP). FLP refers to a mixture of Lewis acid and base forming electron donor–acceptor adduct, while they are not combined due to steric hindrance. Owing to the confinement of MOFs, the introduction of frustrated Lewis acid and base moieties becomes a handy but powerful paradigm for catalysis. The Ma group have conducted systematic work in this field. In 2018, Ma and co-workers immobilized a Lewis acid B(C_6_F_5_)_3_ and a Lewis base 1,4-diazabicyclo[2.2.2]octane (DABCO) onto MIL-101(Cr).^668^ One end of DABCO was coordinated with the open metal site of Cr clusters, while the other end can pair with B(C_6_F_5_)_3_. The resultant MIL-101(Cr)-LP showed 100% yield in the catalytic reduction of imine. The FLP, due to its electron donor–acceptor structure, can activate dihydrogen to form H_2_ adduct. The activated MIL-101(Cr)-FLP-H_2_ was proved as an efficient hydrogenation reagent for α,β-unsaturated imines.^669^ In addition, chirality can be introduced into FLPs through rational design. In 2022, Ma, Tang, and co-workers successfully incorporated chiral FLPs (CFLPs) into MIL-101(Cr), which enabled the asymmetric hydrogenation of imines with high enantioselectivity and superior recyclability.^670^

As a short summary, introducing Lewis acid and base into MOFs inherits the structural tunability of homogeneous catalysts and the recyclability of heterogeneous systems, prompting diverse directions for developing highly selective and efficient catalysis.

### 4.3 Hydrophobic Pore Environment

**Figure 48:**
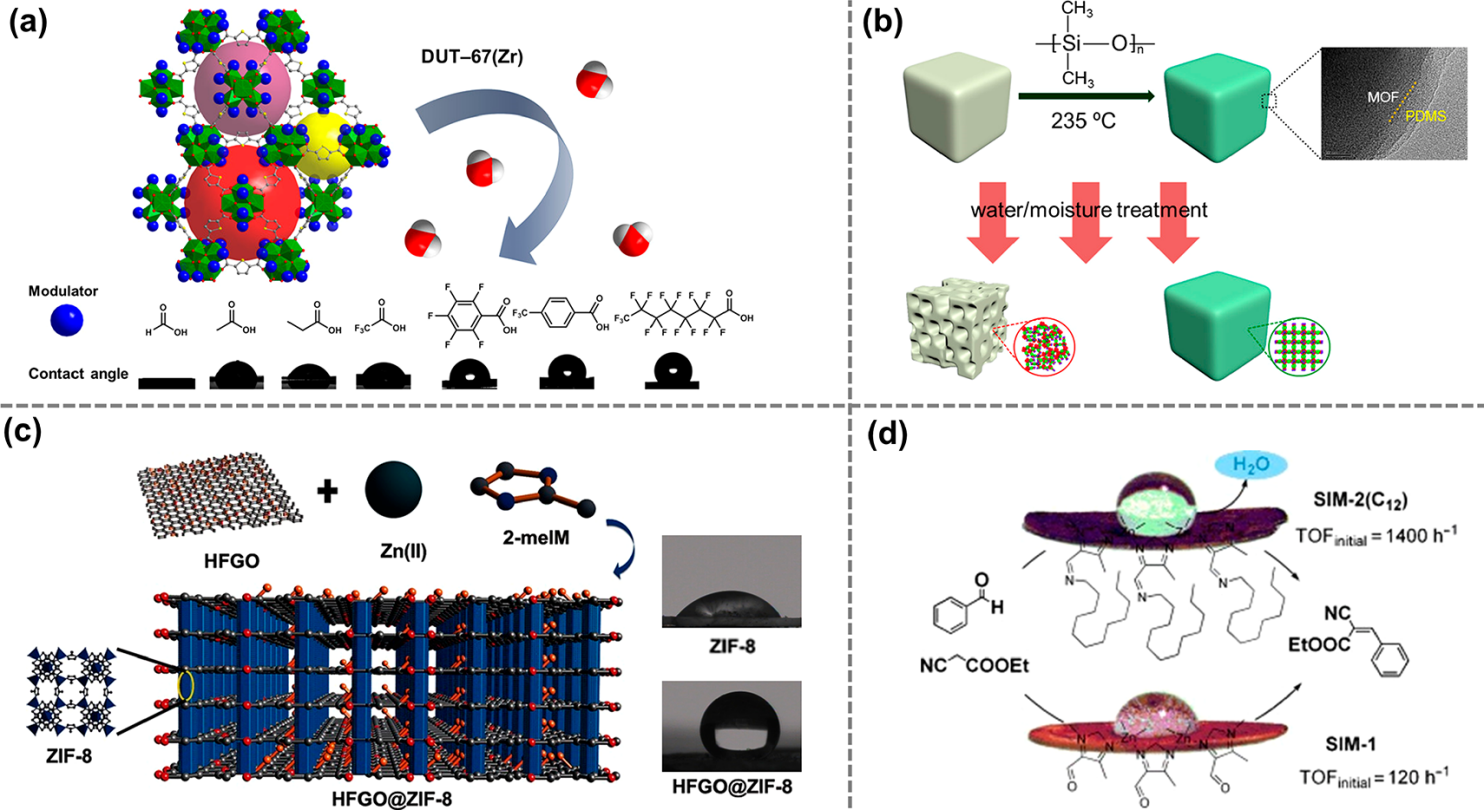
Overview of strategies to synthesizing hydrophobic MOFs or MOF-based composites. (a) Introducing hydrophobic modulator acid into the MOF. Reproduced with permission from ref (674). Copyright 2016 American Chemical Society. (b) Coating the MOF surface with poly(dimethylsiloxane) (PDMS). Reproduced with permission from ref (675). Copyright 2014 American Chemical Society. (c) Fabricating composites consisting of MOFs and highly fluorinated graphene (HFGO). Reproduced with permission from ref (679). Copyright 2016 John Wiley and Sons. (d) Functionalizing MOFs with aliphatic chains through covalent bonds. Reproduced with permission from ref (681). Copyright 2011 John Wiley and Sons.

Water, a strong poisoning molecule in many catalytic reactions, can quench the active species and lower catalytic yield in the corresponding reactions. Modifications of the pore environment can significantly affect the hydrophobicity. To introduce hydrophobicity into the framework,^671,672^ grafting hydrophobic units with fluorine and/or alkyl/aromatic groups on to ligands or metal clusters (Figure 48a),^673,674^ coating the hydrophilic MOFs with hydrophobic shells, such as COFs and SiO_2_, reduced graphene oxide (rGO) and poly(dimethylsiloxane) (PDMS) (Figure 48b),^675−678^ growing MOFs in a 2D hydrophobic highly fluorinated graphene (HFGO) or fluorinated graphene oxide (FGO) layer (Figure 48c),^679^ are three strategies to introduce hydrophobicity.

For the instance of hydrophobic units introduction, the Zhong group modified 4,4′-bipyridine linkers into 2,2′-dimethyl-4,4′-bipyridine, 3,3′-dimethyl-4,4′-bipyridine, each of them yielding MOF-508, SCUTC-18, and SCUTC-19 with the addition of BDC linkers.^680^ The addition of methyl groups excludes water from the confined pore space, boosting the MOF’s stability in moisturized air. The water-repelling feature of MOF pores in catalysis has been systematically studied by the Farrusseng group, who discovered SIM-1, a ZIF with free aldehyde moieties, which can bind with C_12_ aliphatic chains through postsynthetic modification (Figure 48d).^681^ The modified SIM-2(C_12_) showed a better catalytic performance for Knoevenagel condensation than the nonmodified SIM-1.

Hydrophobic coating is a direct approach to protect moisture-sensitive MOFs. In 2014, Yu and co-workers developed a strategy to modify hydrophobic polydimethysiloxane (PDMS) on the surface of MOF-5, HKUST-1, and ZnBT, which are representative moisture-sensitive MOFs. The MOFs were treated in air at 55% relative humidity for 1 day. The surface areas were nearly 100% retained with PDMS-coating, while gas uptake was lost for pristine MOFs.^662^ The Son group coated UiO-66–NH_2_ with a microporous organic network (MON). The MOF@MON hybrid structure showed water resistance, while the original MOF showed good wettability in water. The adsorption of toluene in water is greatly enhanced with a hydrophobic MOF@MON structure.^675^ Another example of hydrophobic coating is conducted by the Jiang group, who incorporated Pd nanoparticles into the pores of UiO-66 and coated the MOF with PDMS.^682^ The Pd/UiO-66@PDMS showed greatly enhanced activity in various catalytic reactions including styrene hydrogenation and reduction of nitrobenzene. Meanwhile, the coating introduces selectivity toward hydrophobic molecules, which is proved by the low yield when nitrophenol was tested as the substrate. In addition, hydrophobic groups can be introduced into MOFs’ ligands through postsynthetic modification. For instance, the Jiang group incorporated perfluoroalkyls into Pd@MIL-101 by amide condensation.^683^ The modified Pd@MIL-101-F_*x*_ (*x* = 3, 5, 7, 11, 15) showed improved preservation of loaded Pd nanoparticles and thus better recyclability. Li and co-workers adopted another route to introduce hydrophobicity into a MOF NH_2_-MIL-101(Fe) by coating it with hydrophobic NTU-COF shell.^684^ The hydrophobic COF shell can concentrate compatible molecules that significantly enhanced the conversion of styrene to benzaldehyde.

### 4.4 Asymmetric Pore Environment

Asymmetric heterogeneous catalysis is an emerging field. Increasing demand for enantiomerically pure compounds in the life sciences and pharmaceutical industry has stimulated the development of asymmetric catalysis. Heterogeneous systems, such as MOFs, have potential advantages of easy product separation, efficient catalyst recycling, improved handling, and process control. The structural tunability of MOFs provides a platform for well-defined linker design and modification, which can create a chiral pore environment to direct asymmetric transformations. The introduction of chirality into MOFs can be achieved through chiral moieties, such as chiral salens,^626,627,629,630,685−697^ 2,2′-bis(diphenylphosphino)-1,1′-binaphthlyl (BINAP),^677^ 1,1′-binaphthol and biphenols,^698−701^ phosphoric acid,^702,703^ proline, and other chiral amino acids.^272,281,704−718^

**Figure 49:**
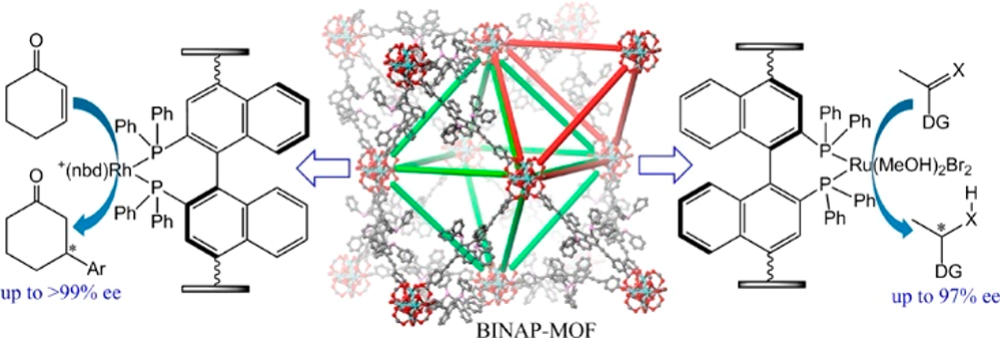
Catalytic activity of a homochiral BINAP-MOF metalated with Rh and Ru. Reproduced with permission from ref (677). Copyright 2014 American Chemical Society.

In particular, the Lin group incorporated ruthenium and rhodium complexes onto BINAP-based MOFs as asymmetric catalytic centers and accomplished enantioselective additions of arylboronic acids to 2-cyclohexenone with up to 99% ee (Figure 49).^677^ Another example is the Lin group’s work with tetracarboxylate ligands. In postsynthetic modification (PSM), they chelated the Ti(IV) metal center onto the linker’s dihydroxy groups to form asymmetric catalysts.^719^ High enantioselectivities were observed in the diethylzinc and alkynylzinc addition to aromatic aldehydes. The dicarboxylate-functionalized *N*,*N’*-ethylenebis(salicylimine) (salen) can also be incorporated into MOFs to induce chirality. The Jiang group built Ni(salen) into MOFs to prepare chiral-centered catalysts for the cycloaddition of CO_2_ with epoxides. The salen-derived framework exhibits a rare [4+4] 8-fold interpenetration with 2-connected Ni(salen) linkers and 4-connected cubic Cu_4_I_4_ clusters nodes. Despite the 8-fold interpenetration, two types of 1D channels were detected, 14.1 × 13.3 Å^2^ and 6.09 × 10.96 Å^2^, respectively. The embedded Ni(salen) moieties possess Lewis acidity and showed up to 84% conversion for the cycloaddition of CO_2_ with styrene oxide. Cycloaddition of epoxides with azides and alkynes was concurrently achieved to produce various β-hydroxy-1,2,3-triazoles at up to 89% yield.^720^

Recently, Cui, Farha, and co-workers incorporated single site Rh species in MOF with chiral spinol-based ligand to afford a chiral catalyst.^721^ The catalysis specifies in monophosphorus Rh anchored by the ligand, demonstrating high efficiency and enantioselectivity in asymmetric hydrogenations of α-dehydroamino acid esters and enamides. In addition, this chiral catalyst can be scaled-up to gram-scale in enantioselective synthesis of chiral drugs.

Chiral amino acids, one enzymatic catalytic site, have been investigated by chemists for efficient enantioselective catalysis. Proline is one of natural amino acids that have been vastly incorporated into MOF pores. In recent years, other chiral amino acids have been reported to assemble efficient MOF-based catalysts.^272,281,704−718^ For instance, in 2021, Manna group harnessed on amino acid-based postsynthetic modification.^722^ Iron was chelated by the tridentate nitrogen-donor ligand in the MOF, to which amino acid was anchored to provide connection and chirality. Even with a 0.5 mol % loading of iron on the MOF, it was able to afford complete hydrosilylation of ketones using (OEt)_2_MeSiH within 2 h. This work suggests an eco-friendly way and amino-acid-inspired way in enantioselective catalysis. In 2021, the Tang group designed three MOF with same ligand derived from 2-aminoterephthalic acid (H_2_BDC-NH_2_).^717^ By grafting the asymmetric unit on ligand, the MOF was presented with both enantioselectivity and light absorptive properties. Three different metal clusters were applied to fine-tune electron transfer properties, resulting in different catalytic performances. Overall, this work developed a new way to build and tune heterogeneous asymmetric catalysts.

### 4.5 Templating Effect

The highly ordered localization of functionalities and pore confinement can synergistically create a template for catalytic reactions, controlling the substrate-binding, electron transfer, and catalyst regeneration. Molecular assemblies and MOFs both satisfy the requirements to foster a templating environment for catalysis, while MOFs’ highly ordered frameworks bring additional effects in creating multiple active sites for tandem catalysis.

**Figure 50:**
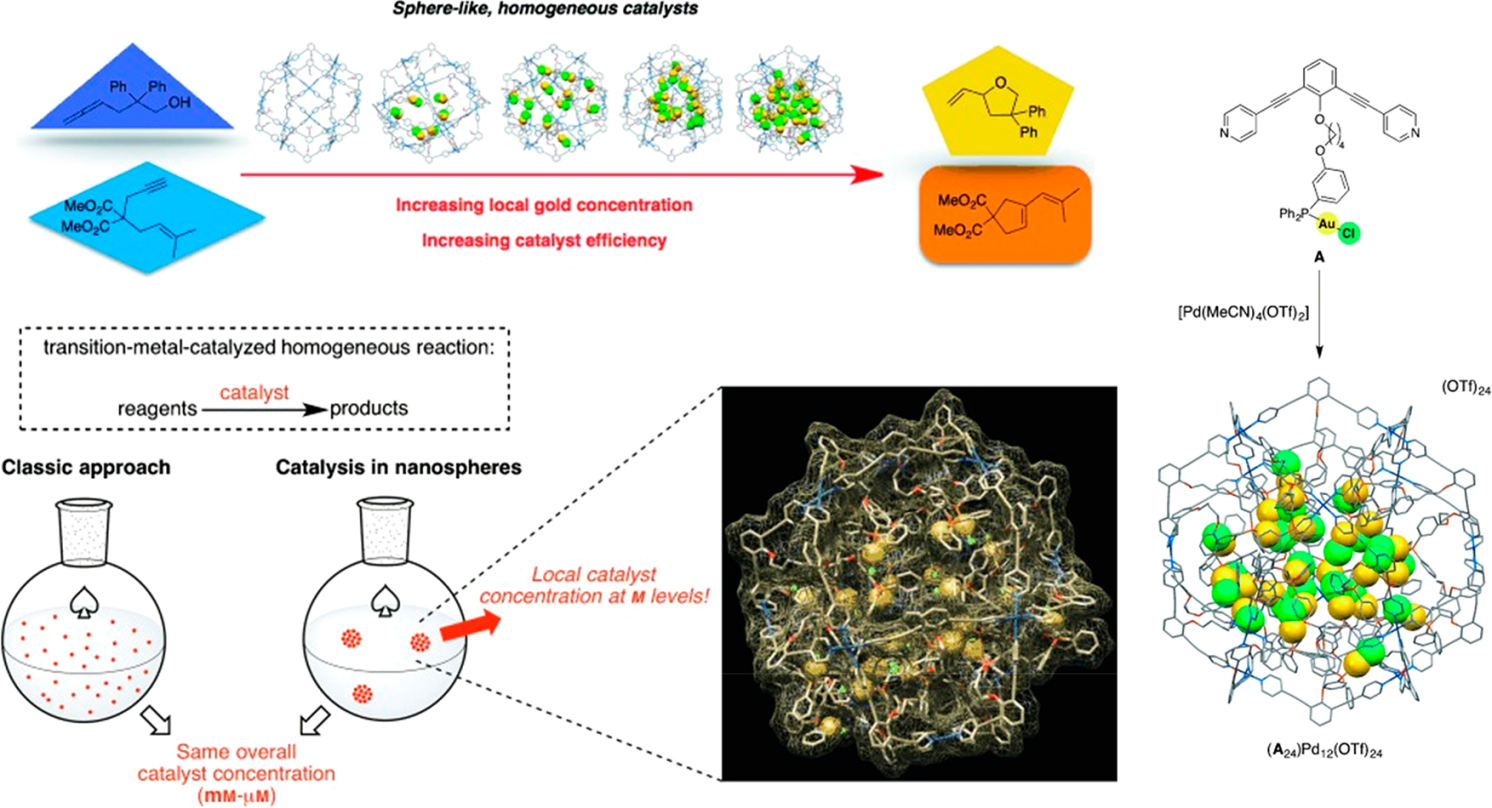
Structural of Pd_12_L_24_ molecular assembly that enables increasing the gold concentration and improving catalytic efficiency. Reproduced with permission from ref (723). Copyright 2014 John Wiley and Sons.

The well-defined structures of molecular assemblies provide ordered binding motifs, where transition-metal catalysts can be attached, achieving the “pre-organization” of catalysts. The preorganization turns out to be a good mimic of the biocatalytic environment. As a pioneer in this field, the Reek group encapsulated gold chloride through terminal-phosphine binding motifs into Pd_12_L_24_ molecular assembly (Figure 50).^723^ Through tuning the ratio between the phosphine-modified and regular ligands, the endohedral concentration of gold catalyst in the cavity can be altered. The local concentration of [AuCl] was tuned between 0.05 and 1.07 M, while 1.07 M brought a 90% yield in the catalyzed hydroalkoxylation of allenol. A switchable gold catalyst encapsulated in a self-assembled hexametric resorcin[4]arene cage was reported by Reek and co-workers.^724^ The limited size of the cage cavity confines the catalyst loading, breaking the dinuclear complex [(Au(NHC))_2_(μ–OH)] into mononuclear units. It was found that the dual-activation pathway is switched into reactivity typical for mononuclear catalysts. The encapsulation of the dinuclear complex provides an on/off switch pathway to avoid the effort to activate the catalyst. The Reek group also studied the reaction rates affected by confinement effects. M_12_L_24_ (M = Pt and Pd) nanosphere was designed with 24-fold endohedral guanidinium-binding motifs.^725^ The self-assembled nanospheres show interior binding to sulfonates and carboxylates through hydrogen bonding. Triphenylphosphinomonosulfonate gold chloride (TPPMSAuCl) was strongly bound to the Pd_12_L_24_ guanidinium binding sites and indicated much higher reaction rates in the cyclization of acetylenic acid upon endohedral preorganization of the gold catalysts. A ruthenium catalyst-loaded version of M_12_L_24_ (M = Pt and Pd) was obtained by their group later.^726^ The ruthenium-loaded Pt_12_L_24_ showed a two-order enhancement of the reaction rate. The preorganization of Ru(bda)(PySO_3_^–^)_2_ promotes the formation of dinuclear radical-oxo intermediate, accelerating the water oxidation reaction taking place in the cavity. Fujita and co-workers achieved a site-isolated cascade reaction.^727^ They mixed two Pd_12_L_24_ molecular assemblies, loading different MacMillan’s catalysts and accomplished oxidation and asymmetric D–A reaction in one pot, whereas the mixture of naked catalysts yielded no products.

**Figure 51:**
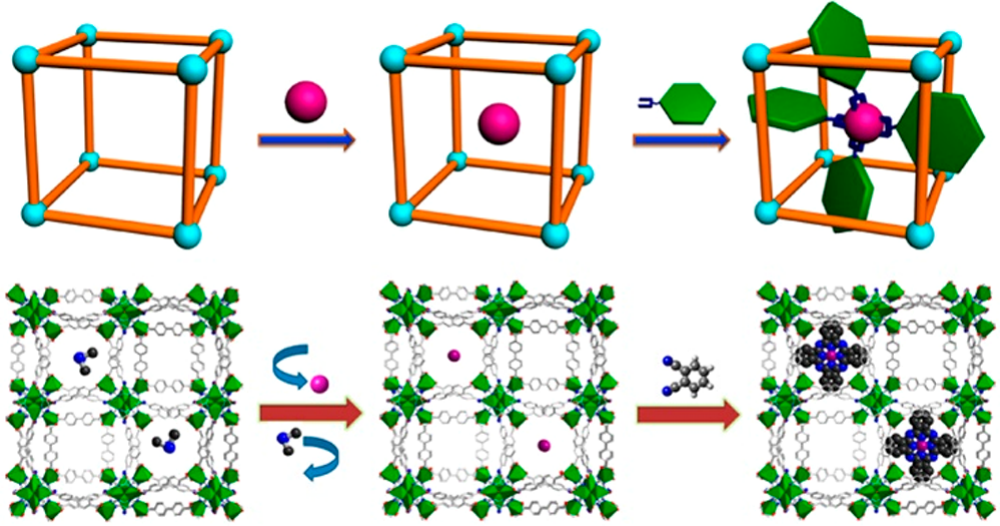
Self-assembly of Co (II) phthalocyanine within the bio-MOF-1’s nanopores, involving metal cation exchange and cation-directed assembly. Reproduced with permission from ref (39). Copyright 2014 American Chemical Society.

In 2014, Ma and co-workers incorporated Co (II) phthalocyanine (Co-Pc) into the nanoporous bio-MOF-1 through *de novo* assembly.^39^ (Figure 51) The encapsulation process involves two steps, namely cation exchange and cation-directed assembly. As a result, the as-formed Co-Pc can be fixed in bio-MOF-1, while the pores of bio-MOF-1 are too small for the ingress of phthalocyanine through direct exchange. The resultant Co-Pc@bio-MOF-1 featured superior conversion in styrene epoxidation compared with Co-Pc in solution because the presence of framework precluded the formation of Co-Pc oligomer. The Co-Pc@bio-MOF-1 composites also indicated size selectivity when varying the substrate molecules, attributed to the inaccessibility of large molecules to the catalytic sites.

In 2017, the Das group encapsulated a Keggin polyoxometalate (POM) [H_6_CoW_12_O_40_] into ZIF-8 to produce a water oxidation catalyst.^728^ The presence of ZIF-8 framework emulated the amino acid residues in photosystem-II, which enable regulating the microenvironment of the oxygen-evolving complex and facilitate oxygen evolution reaction in biological systems. Spectroscopic characterizations indicate electronic redistribution of the polyoxometalate cluster inside the MOF, favoring reversible electron transfer. As a result, the POM@ZIF-8 serves as a highly stable and efficient catalyst for water oxidation in neutral pH. Later, Farha and co-workers observed one interesting phenomenon in a hierarchically porous MOF named NU-1000, where the POM could migrate from mesopores to micropores under mild activation conditions.^729^ Note that the relocation of POMs can also lead to differed reaction rates and product selectivities in the catalytic oxidation of 2-chloroethyl ethylsulde, depending on the accessibility of the POM in pores with varied sizes.

In 2019, Fu, Smoukov, and co-workers confined nanosized RuO_2_ into MOF-808 and attained a MOF-supported catalyst with excellent performance in CO oxidation below 150 °C, which can be attributed to the weaker interactions between the confined RuO_2_ and CO/O species, precluding adsorption-induced surface passivation of catalysts.^730^ This work demonstrates an example of how the templating framework affects the catalytic functionality of guests.

Recently, Yang, Schröder, and co-workers successfully immobilized monoiron hydroxyl sites into PMOF-Ru, which is UiO-67 embedded with a photosensitizer [Ru^II^(bpy)_2_(bpydc)] and a polyvanadotungstate [PW_9_V_3_O_40_]^6–^ (bpy = 2,2′-bipyridine; H_2_bpydc = 2,2′-bipyridine-5,5′-dicarboxylic acid).^731^ Impressively, the resultant PMOF-RuFe(OH) demonstrated ability to activate the C–H bond of CH_4_ and convert CH_4_ to CH_3_OH with 100% selectivity, ascribed to the synergic cooperation of photosensitizers, polyvanadotungstates, and monoiron hydroxyl sites. Mechanism studies suggest that the CH_4_ has a lower energy barrier to generate •CH_3_ radicals when adsorbed at the iron-hydroxyl sites. Overall, the entire framework serves as a porous matrix to promote the synergy among all active species and attain superior catalytic activities.

**Figure 52:**
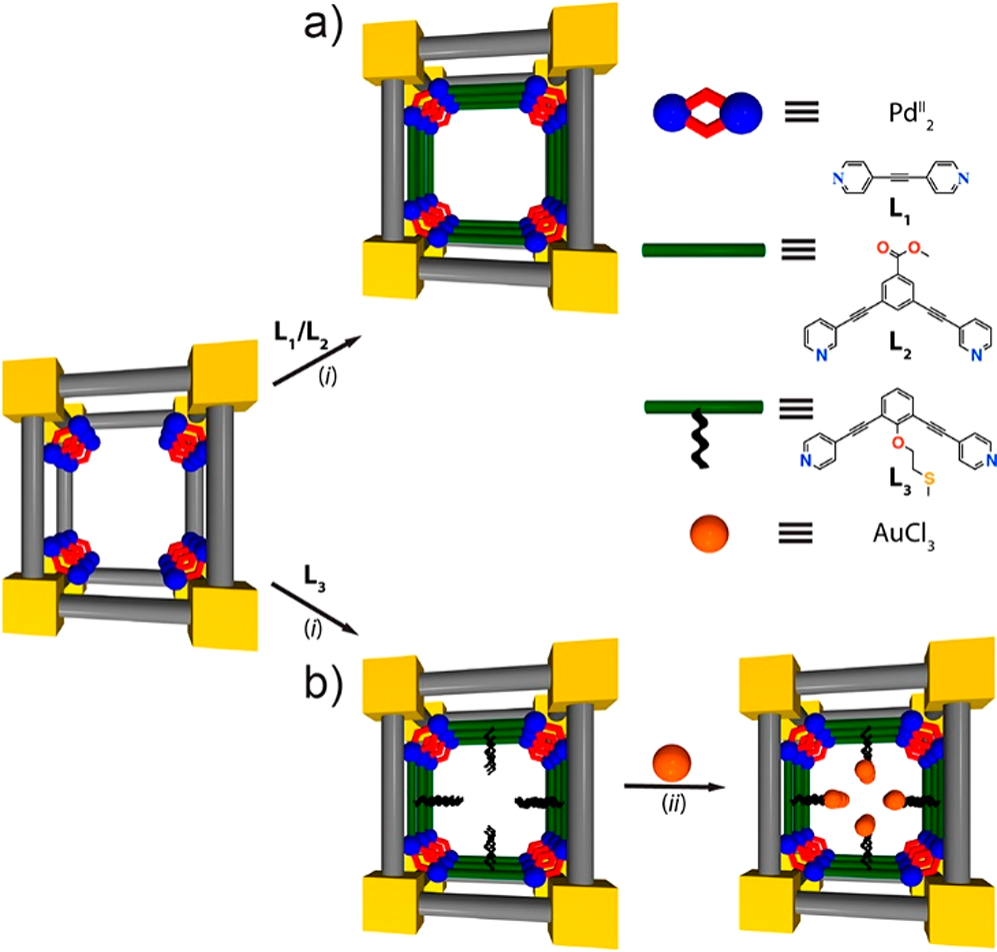
MOF-templated stepwise synthesis of homo- (a) and heterobimetallic (b) supramolecular coordination compounds within the confined MOF channels. Step (i) indicates the incorporation of organic ligand with desired structural and coordination information. Step (ii) indicates postsynthetic metalation. Reproduced with permission from ref (732). Copyright 2019 American Chemical Society.

MOFs can also template the growth of supramolecular coordination compounds within their cavities. In 2019, Ferrando-Soria, Pardo, Armentano, and co-workers presented an elite example of *in situ* self-assembly of metal–organic polyhedrons templated by the confined MOF channels^732^ (Figure 52). Impressively, three supramolecular compounds can be constructed within the MOF, including a Pd_8_ square metal–organic polygon, a Pd_16_ cage, and a bimetallic Au–Pd cage. The mechanical bonds between frameworks and as-formed supramolecular compounds stabilize the Pd catalysts under reaction conditions, leading to higher catalytic activity and selectivity compared with the corresponding compounds in solution.

MOFs feature cavities with well-defined geometries and tunable sizes, providing an appropriate platform for the preorganization of endohedral functionalities. Compared to the templating effects in molecular cages, MOFs have a stabilized confined space that allows the functionalities and installed linkers to gain extra stability. Also, the diffusion limit prevents the loss of crystallinity inside the MOF cavities, maintaining the templating moieties. The advantages covered above make MOFs a potential candidate for enzyme-mimetic catalysts.

### 4.6 Multiple Active Sites

**Figure 53:**
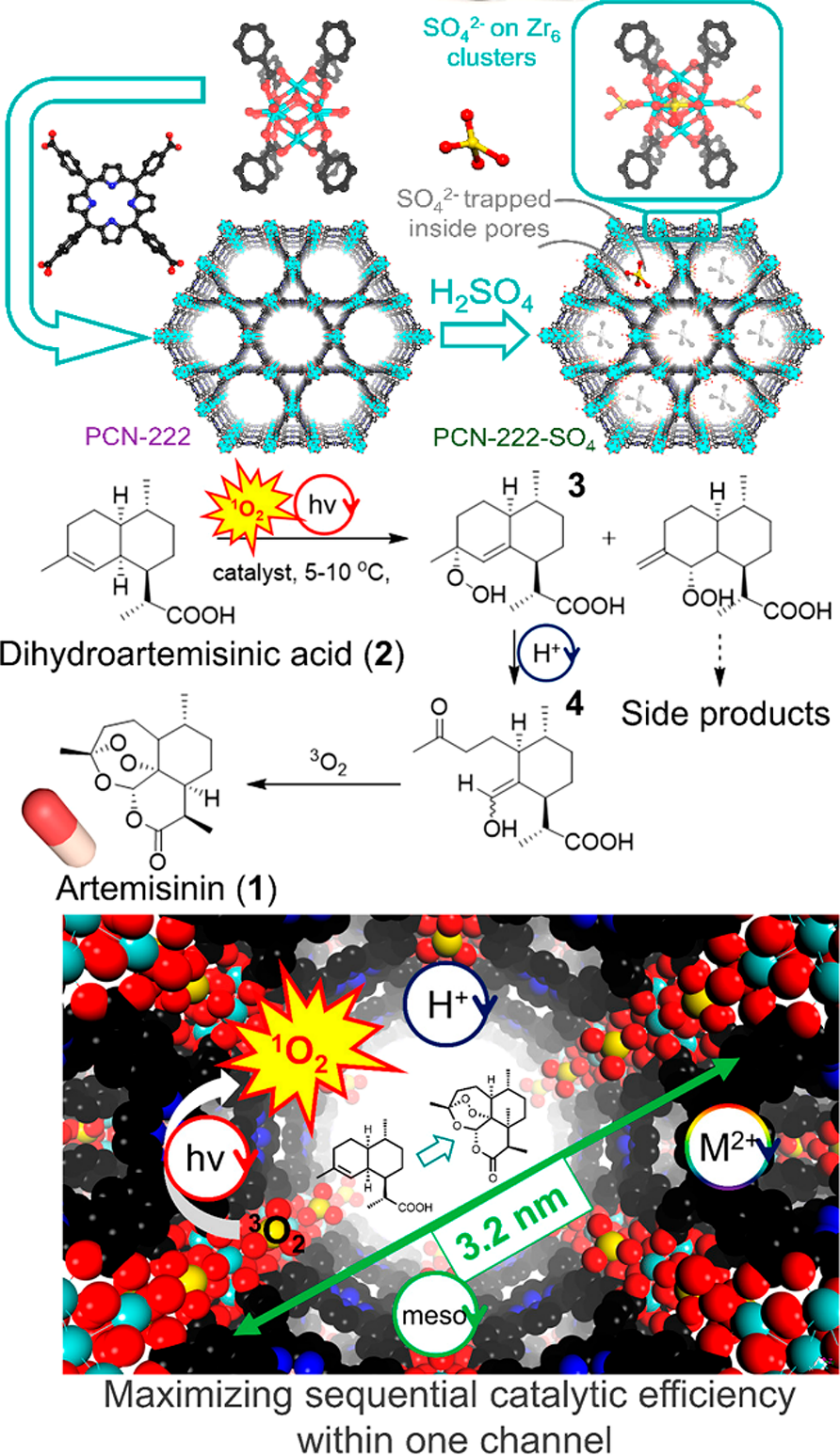
Installing sulfuric acid onto PCN-222 to generate a photoactive and acidic MOF, which can catalyze photocatalytic oxidation of dihydroartemisinic acid to artemisinin. Reproduced with permission from ref (733). Copyright 2019 American Chemical Society.

MOFs’ extended framework structure allows the incorporation of dual-active sites.^394^ The Zhou group crafted the porphyrin metal–organic frameworks with catalytically active porphyrin centers and handy postsynthetic modification to introduce additional active centers (Figure 53).^733^ The metal porphyrins can be coordinated with 8-connected Zr_6_ clusters to afford a MOF named PCN-222, which was then treated by an aqueous H_2_SO_4_ solution to form superacidic PCN-222-SO_4_. The semisynthetic photochemical preparation of artemisinin from dihydroartemisinic acid was achieved in the pores of PCN-222-SO_4_. Dual-catalysis takes place in the channel of PCN-222-SO_4_, with the metal porphyrin-catalyzed photogeneration of ^1^O_2_ and the acidification by the proton donated by the hydrogen sulfate. The multiple functionalities embedded in the MOF channels enable cooperation to achieve dual active-site catalysis.

**Figure 54:**
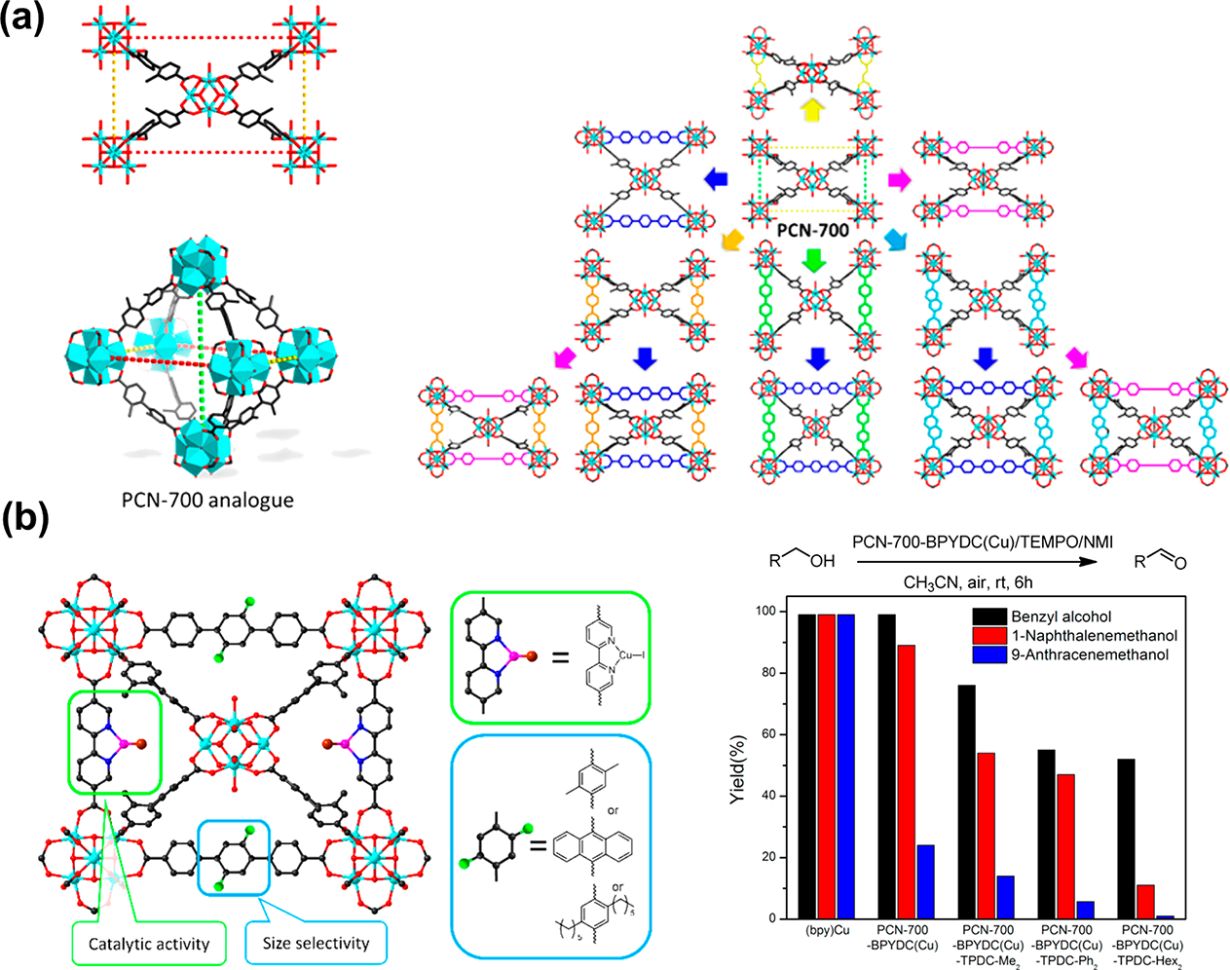
Construction of catalytic MOFs through postsynthetic linker installation. (a) PCN-700 features two types of missing-linker defects, which can accommodate carboxylate ligands with varied sizes. (b) Active sites were introducing to endow the MOF with catalytic activity. Reproduced with permission from ref (256). Copyright 2016 American Chemical Society.

Due to their diverse linker functionalities, complex pore environments, and cooperative catalytic sites, multicomponent MOFs feature outstanding capability in heterogeneous catalysis (Figure 54). Upon connectivity reduction, UiO-66, constructed from Zr_6_ clusters and 1,4-benzenedicarboxylate (BDC), can generate highly defected frameworks that open up coordination sites on Zr_6_ clusters, which can be used to incorporate other functional moieties. The Zhou group used the solvothermal reaction of ZrCl_4_, BDC, NiTCPP, and benzoic acid to prepare a mix-linker MOF NiTCPP⊂UiO-66.^734^ Through a similar approach, FeTCPPCl modified UiO-66 was obtained, showing a high catalytic activity for the oxidation of 2,2′-azino-bis(3-ethylbenzthiazoline-6-sulfonic acid) (ABTS) in the presence of hydrogen peroxide. The Yaghi group functionalized the UiO-66 with sulfonic acid (−SO_3_, S) and ammonium (−NH_3_^+^, N) and then incorporated Pt nanoparticles.^735^ In the gas-phase transformation of methylcyclopentane (MCP) to acyclic isomer, olefins, cyclohexane, and benzene, Pt⊂nUiO-66-S yielded the highest selectivity to C_6_-cyclic products without acyclic isomeric products (62.4% and 28.6% for cyclohexane and benzene, respectively), which is double of the nonfunctionalized Pt⊂nUiO-66. However, Pt⊂nUiO-66-N decreased the selectivity for C_6_-cyclic products to less than 50%, increasing the acyclic isomer selectivity to 38.6%. The mixed-linker Pt⊂nUiO-66-SN made benzene the dominant product. The varied catalytic activity proved the effect of systematic functionalities on the organization of Pt nanoparticles.

**Figure 55:**
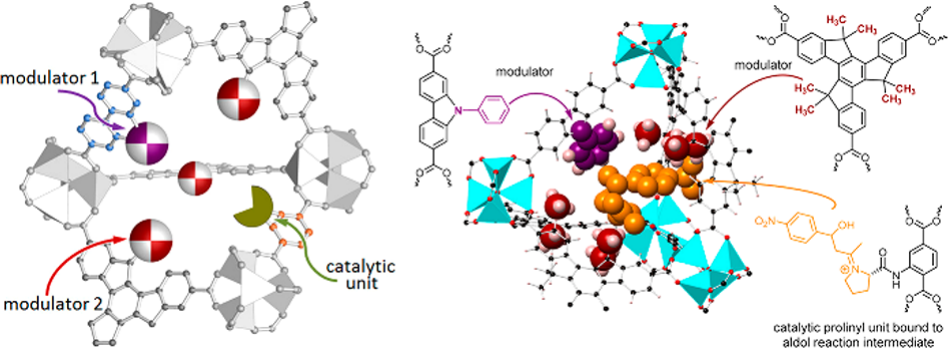
Illustration of the pore environment in MUF-77 equipped with catalytic unit and modulator groups. The potential contacts between the aldo intermediate (orange) and the modulator groups (violet and red) are shown in the right. Reproduced with permission from ref (711). Copyright 2017 American Chemical Society.

Extending dual-active sites into multiple active sites routes this chemistry to biomimetic catalytic systems. The Tsung group prepared a MOF composite where a zirconium-based MOF, UiO-66, hosts two ruthenium PNP pincer complexes and ruthenium PNN pincer complexes (PNP = 2,6-bis((di-*tert*-butylphosphino) methyl) pyridine, PNN = 6-((di*-tert*-butylphosphino)methyl)-2,2′-bipyridine).^736^ Varied functionalized linkers were utilized to compose UiO-66-X (X = CH_3_, F, Br, NO_2_, NH_2_, and NH_3_^+^) and a perfluorinated variant of UiO-66 (X = 4F) was also assessed as host materials. In mimic of the enzyme RuBisCo secondary-sphere interactions, hydrogenation of carbon dioxide to methanol was achieved in the RuPNP@UiO-66-X composites. Encapsulated RuPNP is capable of hydrogenation of carbon dioxide to formic acid. The Zr_6_ (OH)_4_O_4_ cluster converts formic acid to formate ester. Subsequently, RuPNN carries out the hydrogenation of formate ester to methanol. Results revealed that −NH_3_^+^ moieties in proximity with the encapsulated Ru complexes could assist the reaction and the outer-sphere from UiO-66-NH_3_^+^ is of vital importance. An unprecedented cumulative TON of 100 000 was recorded for the composite. Another instance for multiple reactive sites is that the Telfer group installed catalytically active Boc-protected H_2_bdc-prolynyl (H_2_bdc-Pro) and H_2_bpdc-prolynyl (H_2_bpdc-Pro) linkers into MUF-77 with tritopic truxene linkers installed as modulators.^711^ (Figure 55) Aldol reaction of acetone and *p*-nitrobenzaldehyde was showcased in the MUF-77 analogues. Remarkably, the participation of multiple functional groups can alter the kinetic rates and enantioselectivity of the reaction. One unanticipated observation is that the microenvironment around the prolinyl group in the MOF system can override the inherent enantioselectivity of the chiral ligand, affording products with reversed enantioselectivity. This work highlights MOFs’ capability in regulating the spatial environment around the catalysis.

Multivariate (MTV)-MOFs with ordered linker distributions can be precisely modulated in pore volume and environment by rational design of linkers.^737^ As a result, a multifunctional catalytic system can be fabricated. The Zhou group prompt the concept of linker installation, an efficient strategy to incorporate additional functionalities and confinement into the pore environment. Zhou and co-workers reported the installation of a series of ditopic linear linkers into PCN-700, in which the linker length varies from 9.1 to 25.9 Å (Figure 54).^256,738^ Matching the cluster-to-cluster distances on different edges, linear linkers with different lengths can be installed between adjacent clusters. Therefore, linear linkers with organometallic functionalities can also be installed. For instance, copper(II)-bipyridinium dicarboxylates (Cu-BPYDC) were installed successfully and showed remarkable catalytic activity in the aerobic oxidation of alcohol. Size exclusion from the confinement effect of the MOF pocket significantly influenced the reactivity. A drop in catalytic yield was observed during switching the substrate from benzyl alcohol to 1-naphthalenemethanol (∼100% to <90%), with a shaper drop further changing to 9-anthracenemethanol (<90% to <30%). In 2020, Zhou and co-workers successfully installed a ruthenium-based metalloligand into a mesoporous Zr-based MOF PCN-808, generating a highly efficient photocatalysts.^739^ The photoactive MOF enabled conducting catalysis of large substrates, attributed to its mesoporosity.

To date, diverse synthetic methodologies have been developed to engineer the chemical compositions, pore sizes, sequence, morphologies, and particle sizes of MOFs.^740−742^ All these parameters are highly associated with the catalytic performance of the materials. Presumably, MTV-MOFs may provide an advanced platform for cooperative catalysis with the involvement of multiple catalytic sites. Furthermore, the tailored pore environment of MOFs may endow the catalyst with superior selectivity and efficiency.

### 4.7 Homogeneous Supramolecular Catalysts

Homogeneous systems, such as supermolecules and coordination cages, have attracted significant attention due to their enzyme-like catalytic performance. Cyclodextrins (CDs), calix[*n*]arenes, and cucurbit[*n*]urils (CBs) are three most studied supramolecular systems, which can also serve as building blocks of MOFs. In addition, coordination cages, assembled through metal nodes and organic ligands, feature the capability to capture substrates and stabilize transition states. Herein, these supramolecular catalysts are discussed in terms of their structures and catalytic activity, which may shed light on designing enzyme mimetic MOFs with tailored pore environments.

#### 4.7.1 Cyclodextrin

**Figure 56:**
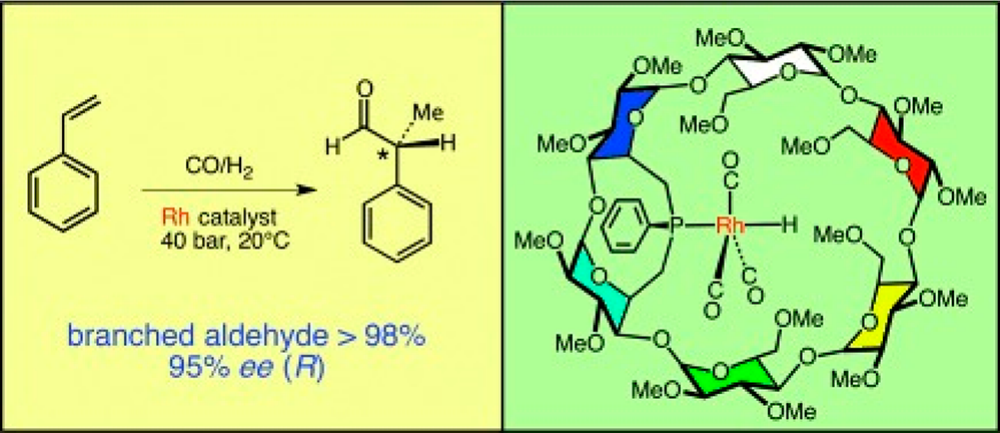
Regio- and enantioselective hydroformylation of Rh-monophosphane complexes confined within cyclodextrins. Reproduced with permission from ref (746). Copyright 2014 John Wiley and Sons.

CDs with well-defined cavities have drawn much attention. They are cyclic oligomers made of α-d-glucopyranoside monomers. The multiple hydroxyl groups allow CDs to be readily soluble in water, forming a defined, hydrophobic cavity. The confinement effects appear to influence the activity and selectivity in catalysis, similar to enzymes. There are three most common oligomers, α-, β-, and γ-CDs, with a cavity diameter ranging from 5.6 to 8.8 Å. They contain two rims formed by a network of hydroxyl groups. Breslow and co-workers did some pioneering work on the confinement effect on selectivity. They reported the regioselective chlorination of anisole confined in the cavity of a CD.^743^ Results show a higher selectivity toward para-substituted products. In comparison, ortho- and para-substituted products are both generated in the normal chlorination of anisole. The combined experimental results indicated that anisole’s ortho- sites are blocked and preserved in CDs. Their later work combined a catalytic nickel-pyridine-carboxaldoxime (Ni-PCA) group onto the hydroxy rim on one side of the CD, which showed four times elevated catalytic rate of the Ni-PCA complex in the catalytic hydrolysis of *p*-nitrophenyl acetate.^5^ A more recent example is Sollogoub’s work on a gold–carbene catalyst encapsulated in the cavity of a cyclodextrin. Through ligand exchange, the gold–carbene catalyst is open on one side. The limited opening of CDs reinforces the selectivity toward the substrate. Specifically, the smaller α-CD gold–carbene catalyst is responsible for five-membered ring products, while the bigger β-CD gold–carbene catalyst leads to six-membered ring products. Other metalloenzymes, including iron–porphyrin complexes, have been sandwiched between two β-CD by Kuroda and co-workers.^744,745^ The confined system showed high selectivity in the epoxidation of cyclohexene, that is, 55% for the β-CD sandwiched iron-porphyrin, <2% for the free iron–porphyrin. A reasonable explanation is that reaction Fe(II)=O species were shielded off by the cavity, whereas they decomposed without confinement. The Matt group extended the CDs to host noble metals by appending a monophosphine–rhodium complex to α-, β-CDs (Figure 56).^746^ Taking advantage of the steric bulkiness of the CDs, the confined rhodium center exclusively bound to a single PR_3_ ligand. The hydroformylation of styrene was proved to be highly regioselective and enantioselective toward the α- addition into the R conformation, affording 95% ee.

**Figure 57:**
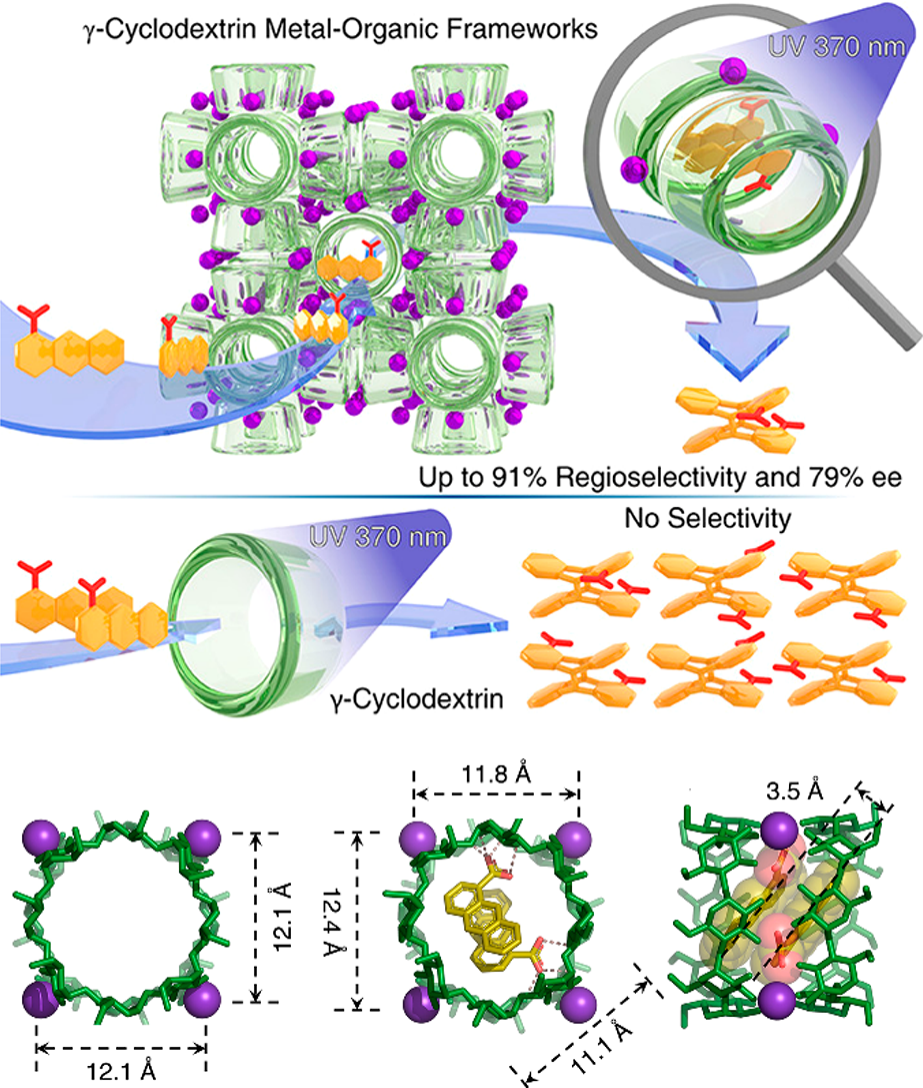
Regio- and enantioselective photodimerization confined within a cyclodextrin-based MOF. The substrates can form superstructures with cyclodextrin frameworks. Reproduced with permission from ref (749). Copyright 2021 American Chemical Society.

The Stoddart group successfully composed CD into MOFs (**CD-MOF-1**).^747,748^ With alkali metal ions, such as K^+^, Rb^+^, etc., γ-CDs are oriented to form confined reaction channels. Moreover, the introduction of metal cations facilitates substrate preorganization in a well-defined manner. CD-MOFs consist of positively charged frameworks on account of the metal cations. They are capable of absorbing anionic substrates postsynthetically into their interconnected tunnels through exchange with OH^–^ anions. By X-ray crystallography, the selective packing of these organic counteranions can be visualized. 1-Anthracenecarboxylate (**1-AC**^**–**^) was encapsulated into the porous tunnels of **CD-MOF-1** with an up to 85% yield (Figure 57).^749^ [4+4] Photodimerization of **1-AC**^**–**^ under UV-light irradiation with regioselectivity up to 91% and enantioselectivity of up to 79% ee. In comparison, the photodimerization of free solvated **1-AC**^**–**^ showed no regio-/enantioselectivity. The innate chirality of γ-CDs allows asymmetric catalysis to be achieved in a metal–organic framework. This work bridges the homogeneous and heterogeneous systems, enabling the building of solid-state superstructures based on crystallographic methodologies.

In 2013, Grzybowski and co-workers integrated a photocatalyst [Ru(bpy)_3_]Cl_2_ (bpy = 2,2′-bipyridine) into a Rb-CD-MOF via a cocrystallization approach.^750^ The [Ru(bpy)_3_]^2+^ was accommodated by the 1.7 nm cavity of the Rb-CD-MOF. Interestingly, the occlusion and confinement of CD-MOF prevent the photodegradation and leaching of the catalyst without affecting its activity. The OH^–^ and ROH groups densely decorated in the CD-MOF can also serve as electron donors to reduce [Ru(bpy)_3_]^3+^ back to the ground state [Ru(bpy)_3_]^2+^. The composite catalyst [Ru(bpy)_3_]Cl_2_@Rb-CD-MOF can photoreduce Pd^2+^, Au^3+^, and Ag^+^ salts into metal nanoparticles.

**Figure 58:**
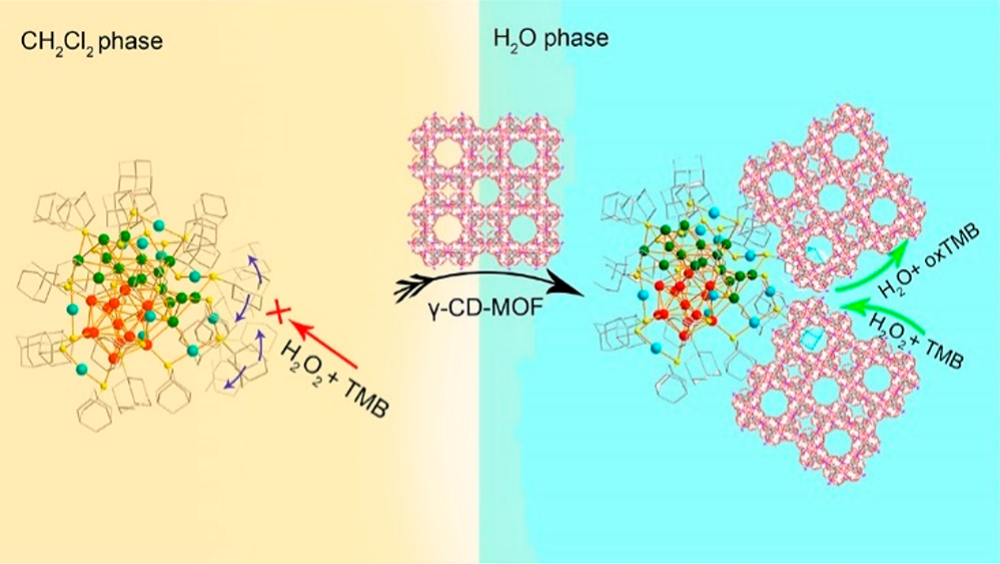
Endowing a gold nanocluster with water solubility and HRP-mimicking catalytic activity through bonding with CD-MOF-1. Reproduced with permission from ref (751). Copyright 2020 American Chemical Society.

In 2020, Wu group reported a dual-purpose strategy to bond an adamantanethiolate-protected gold nanocluster Au_40_ (S-Adm)_22_ with CD-MOF-1.^751^ As tested by the oxidation of 3,3′,5,5′-tetramethylbenzidine (TMB), the introduction of the CD-MOF-1 can not only improve the water solubility of the system but also endow the gold nanocluster with efficiency and activity for horseradish peroxidase (HRP)-mimicking catalysis (Figure 58).

#### 4.7.2 Calix[*n*]arene

Calix[*n*]arenes are a group of supramolecules with explicit and handy synthesis.^752−754^ The first-developed calix[*n*]arenes are oligomers of 2-methylene-1-phenol.^755^ The phenol groups lie on one rim, and the phenyls orient toward the other rim. Moreover, the methylene bridging groups can be switched into ether (−CH_2_OCH_2_−), thio (−S−), aza [−CH_2_N(R)CH_2_−], sulfoxide (−SO−), and sulfone (−SO_2_−). The Reinhoudt group did pioneering studies on the cavity of calix[*n*]arenes in biomolecular catalysis. They dscribed the synthesis of M-[12]aneN_3_ (M = Zn, Cu) attached calix[4]arenes. In intramolecular transesterification of diribonucleoside monophosphate UpU. The Cu_2_ and Cu_3_ calix[4]arenes showed a 160- and a 200-fold catalytic activity compared to the mononuclear Cu_1_-complex, emphasizing the importance of multiple metal centers’ synergistic effects in biomolecular catalysis. The Karakhanov group incorporated palladium(II) metal ions into a range of water-soluble calix[*n*]arenes as active centers for Wacker oxidation of linear alkenes.^756^ The transfer from the calix[4]arene to the calix[6]arene led to the variation of the substrate selectivity: the increase of cavity size correlates with the higher oxidation rate of alkenes with a longer carbon backbone. Palladium centers are active in Suzuki–Miyaura, Kumada–Tamao–Corriu, and Mizoroki–Heck cross-coupling reactions. The Matt group systematically studied calixarenyl-phosphines’ catalytic activity in Suzuki–Miyaura cross-coupling of phenylboronic acid with aryl halides.^757^ These catalysts showed significantly higher turnover frequency (TOF) compared to the free Pd triarylphosphine complexes due to the calix[4]arenes’ ability to trap MArX units that result in a highly crowded metal environment, leading to the formation of monoligated intermediates, these being more reactive than bis-ligated complexes. Moreover, the nickel-chelated calixarenyl phosphines showed the same enhanced catalytic rate originating from the cavity confinement effect in Suzuki–Miyaura and Kumada–Tamao–Corriu cross-coupling reactions.^758^ They suggested that the respective aryl-halide species in the transient [M(π–ArX)calix-phosphine] intermediate created a bulky environment around the active center through noncovalent interactions in the cavity, which promoted the formation of monoligand Pd(0) and Ni(0) intermediates. An example of asymmetric catalysis was the aldol reaction catalyzed by l-proline-calixarene-derived achiral thiourea host–guest complex.^759^ The thiourea-containing cavity of the calix[4]arene can stabilize the transition state by hydrogen bonding. Optimized catalytic results showed that under specific reaction conditions, especially in nonpolar solvents, an enantioselectivity of 99% ee can be obtained. Interestingly, the catalysts proved to be functional in water, which is otherwise an unfavored catalytic condition for most organometallic catalysts. However, a decreased enantioselectivity was observed in aqueous solution. Furthermore, the Yilmaz group designed a chiral calix[4]arenes-bearing prolinamide catalyst for asymmetric aldol reactions in water, affording a conversion of 98% with high enantioselectivity (90% ee) and diastereoselectivity (anti:syn = 91:9).^760^ Another strategy in asymmetric catalyst design is to introduce chirality into calix[*n*]arenes. Li and co-workers provided a number of synthetic routes for inherently chiral calix[4]arenes and tested them on asymmetric Michael addition reaction, which showed moderate enantioselectivities, with up to 15% ee.^761^ Recently, Karpus and co-workers designed another achiral calix[4]arene phosphonic acid which shows excellent catalytic activity, up to 95% yield, in asymmetric aza-Diels–Alder reactions. However, the enantioselectivities still need improvement, with the highest being 21% ee.^762^

**Figure 59:**
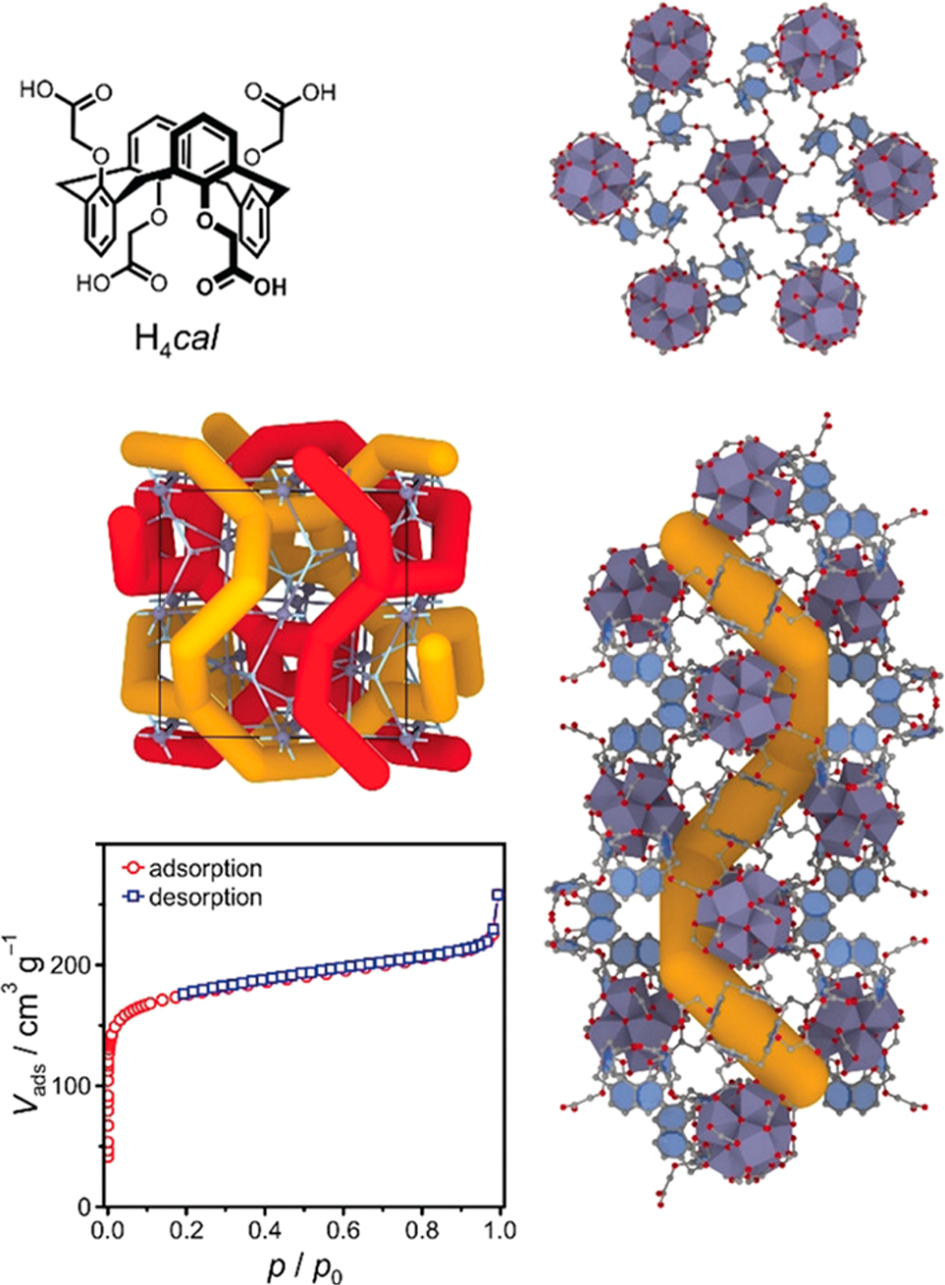
Structural illustration of the calix[4]arene linker and the (4,6)-connected MOF. The two nonintersecting pores are depicted in the framework in red and orange. The N_2_ sorption isotherm at 77 K is displayed. Reproduced with permission from ref (764). Copyright 2018 John Wiley and Sons.

To date, reports on calixarene-based MOFs are still few, and most of them merely focus on the structural design of the framework. For instance, in 2012, Bew, Burrows, Düren, and co-workers developed four MOFs using a calix[4]arene dicarboxylic acid and four distinct metal(II) salts, namely Cu^2+^, Zn^2+^, Cd^2+^, and Co^2+^.^763^ In the ligand, both the carboxylate groups were functionalized on the upper rim of the calix[4]arene. The resultant MOFs featured two types of pores associated with the integral frameworks and the calix[4]arene ligand. In 2018, the Schaate group reported a Zr-based calix[4]arene MOF, in which the 4-connected calix[4]arene ligand adopts *C*_2_ symmetry and the 6-connected Zr_6_ cluster shows *C*_3i_ symmetry.^764^ (Figure 59) The resultant (4,6)-connected framework features a BET surface area of 670 m^2^ g^–1^ and two types of identical and nonintersecting pores. The accessible calix[4]arene enables the encapsulation of NO_2_, generating colored charge-transfer complexes for visual detection of NO_2_. In 2020, Ling and co-workers reported a series of lanthanide(III) coordination polymers by using sulfonated calix[4]arene and calix[6]arene, which exhibit porosity according to the crystal structures.^765^ Besides, calixarenes can also serve as building blocks for nanoporous materials based on host–guest interactions^766^ and dynamic covalent bonds.^767^

A recent report from the Su group indicates that a cone-calix[4]arene bearing D-π–A units can sensitize a Pt@UiO-66-NH_2_ composite, boosting the hydrogen production activity of the catalyst.^768^ In 2018, Isaeva, Timofeeva, and co-workers incorporated calix[4]arenes with acidic functionalities into the MOF NH_2_-MIL-101(Al).^769^ Calix[4]arenes with differed Lewis acidity would lead to varied conversion and selectivity during the synthesis of 1,5-benzodiazepine, attributed to the hydrogen bonding interactions between the calixarene functional groups and amino groups in the MOF.

Given the abundant functionality, inner cavity, high structural symmetry, and engaging catalytic activity of calixarenes, more and more calixarene-based framework materials are expected in the future, which will diversify both the field of porous materials and supramolecular catalysts.

#### 4.7.3 Cucurbituril

**Figure 60:**
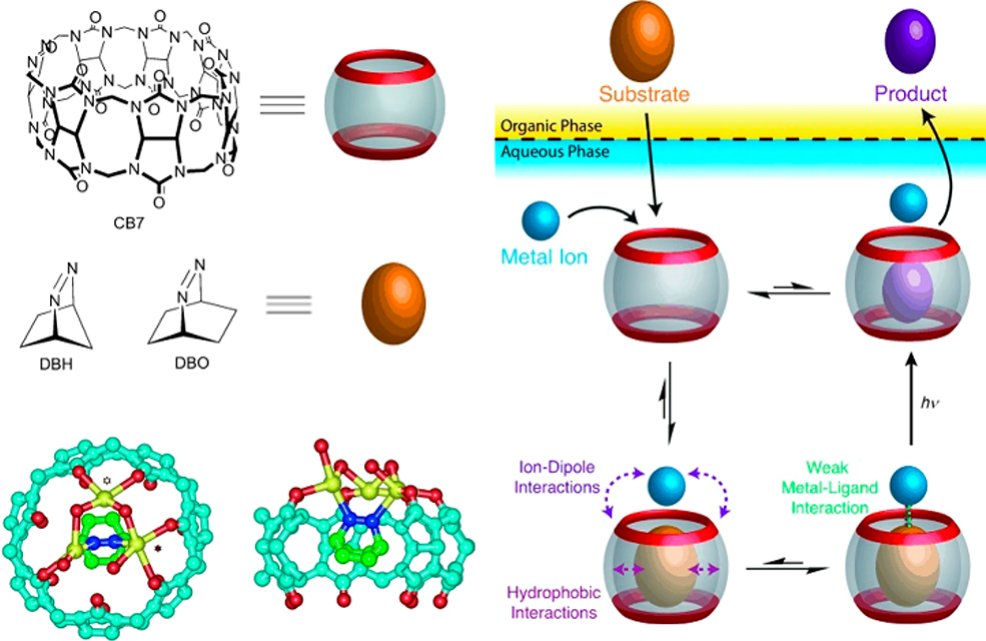
Dynamic self-assembly of a guest/host/metal–cation complex. The transition metal promoted the selective photoreactions within the cucurbiturils. Reproduced with permission from ref (773). Copyright 2011 John Wiley and Sons.

Cucurbiturils (CBs) are a class of molecular containers formed by copolymerization of formaldehyde, glyoxal, and urea.^770^ CBs were first discovered as CB[6] ([6] refers to the number of urea units in the overall shape). Progressively, the chemistry of CB has been expanded as different CB[*n*]s (*n* = 5–10) were prepared. One interesting feature of this supermolecule is the capability to bind both polar and nonpolar organic molecules due to the carbonyls sitting at the entrance of the cavity and inside the cavity. Mock and co-workers utilized CBs in catalyzing the cycloaddition of alkynes and azides.^771,772^ Confirmed by the recent computational results from Carlqvist and Maseras, the alkyne and azide substrates are encapsulated inside the host cavity due to the presence of ammonium groups. The 1,3-dipolar cycloaddition was accelerated by a factor of 5.5 × 10^4^ under the influence of cucurbituril. The entropy cost of bringing the two substrates together was lowered through the formation of a stable ternary complex between the reactants and CB[6], making the reaction unimolecular. Nau and co-workers report another important work on varied types of transition-metal incorporated CB[7]s, including Ti^4+^, Fe^3+^, Co^2+^, Ni^2+^, Cu^2+^, and Ag^+^. They performed promising activities in the chemoselective photoreactions of azoalkanes, where the transition metals played a critical role in the chemoselectivity of product formation (Figure 60).^773^ The Herrmann group designed a three-component supramolecular CB[*n*]-based system containing amino acids, Cu^2+^ cations, and CB[8], assembled into a nanoreactor.^774^ The combination of amino acids created a chiral environment, with Cu^2+^ being the active site. The Diels–Alder reaction of azachalcone with cyclopentadiene produced highly chiral products with the presence of the CB catalyst, which otherwise yielded racemic products in the absence of the catalyst. The Zhang group later reported the stabilization of 2,2,6,6-tetramethylpuperidin-1-oxyl cation (TEMPO^+^) by CB[7], and this effect resulted in higher conversion to the corresponding aldehyde in the biphasic oxidation of alcohols by the oxidant TEMPO and NaClO.^775^

One of the earliest reports of CB-based coordination polymers was from Fedin group in 2008, in which a series of tetranuclear rare earth clusters were sandwiched between two CBs.^776^ In 2015, Su, Song, and co-workers introduced CB[6]-based pseudorotaxanes into MOFs through a mixed-linker strategy, affording materials with luminescence.^777^ In 2016, Cao, Lü, and co-workers assembled a porous coordination polymer by using CB[6] and NaI, which can act as an absorbent to uptake iodine.^778^ Later, the Sun group reported a CB[7]-based metal–organic rotaxane framework with capability for capturing iodine and potassium cation.^779^ Recently, Janiak and co-workers developed a mechanochemistry approach to encapsulating decamethylcucurbit[5]uril into MIL-100(Fe), and the resultant hybrid material featured enhanced performance in Pb^2+^ removal and CH_4_ uptake.^780^

**Figure 61:**
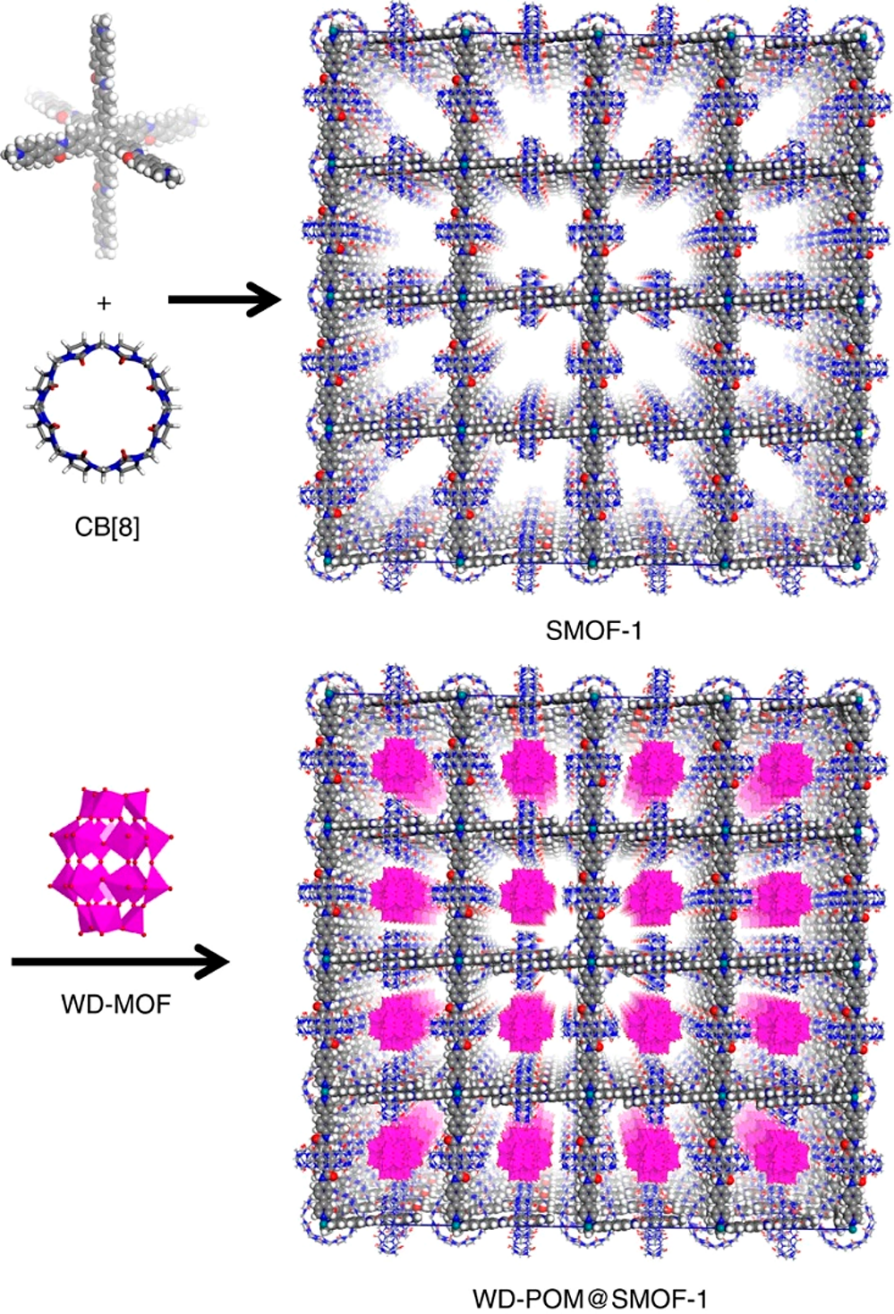
Structural illustration of the SMOF-1 and WD-POM@SMOF-1. Carbon, nitrogen, oxygen, and hydrogen are represented in gray, blue, red, and white, respectively. Reproduced with permission from ref (781). Copyright 2016 Springer Nature.

The application potentials of CB-based MOF have not been fully uncovered yet. One rare example of catalytic CB-based MOFs is reported by Li, Liu, Wang, and co-workers, who assembled CB[8] and a hexaarmed [Ru(bpy)_3_]^2+^-based ligand to form a supramolecular metal–organic framework named SMOF-1.^781^ Furthermore, anionic Wells–Dawson-type polyoxometalates (WD-POMs) can be absorbed into the SMOF-1 to produce a hybrid photocatalyst for visible-light-driven hydrogen production. (Figure 61)

#### 4.7.4 Self-Assembled Container Molecule

**Figure 62:**
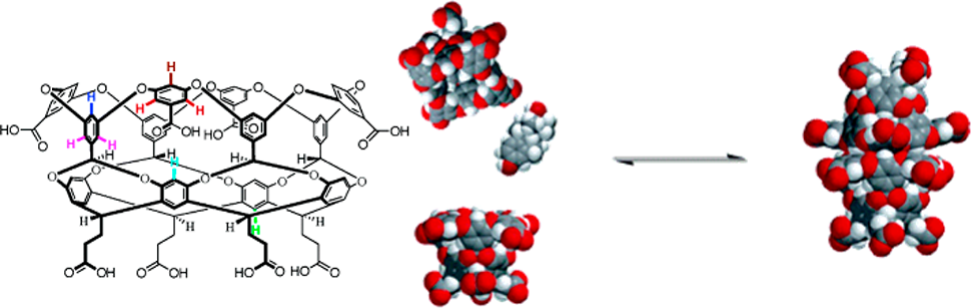
Self-assembly of cavitands to form a capsule to store steroids. Reproduced with permission from ref (783). Copyright 2004 American Chemical Society.

**Figure 63:**
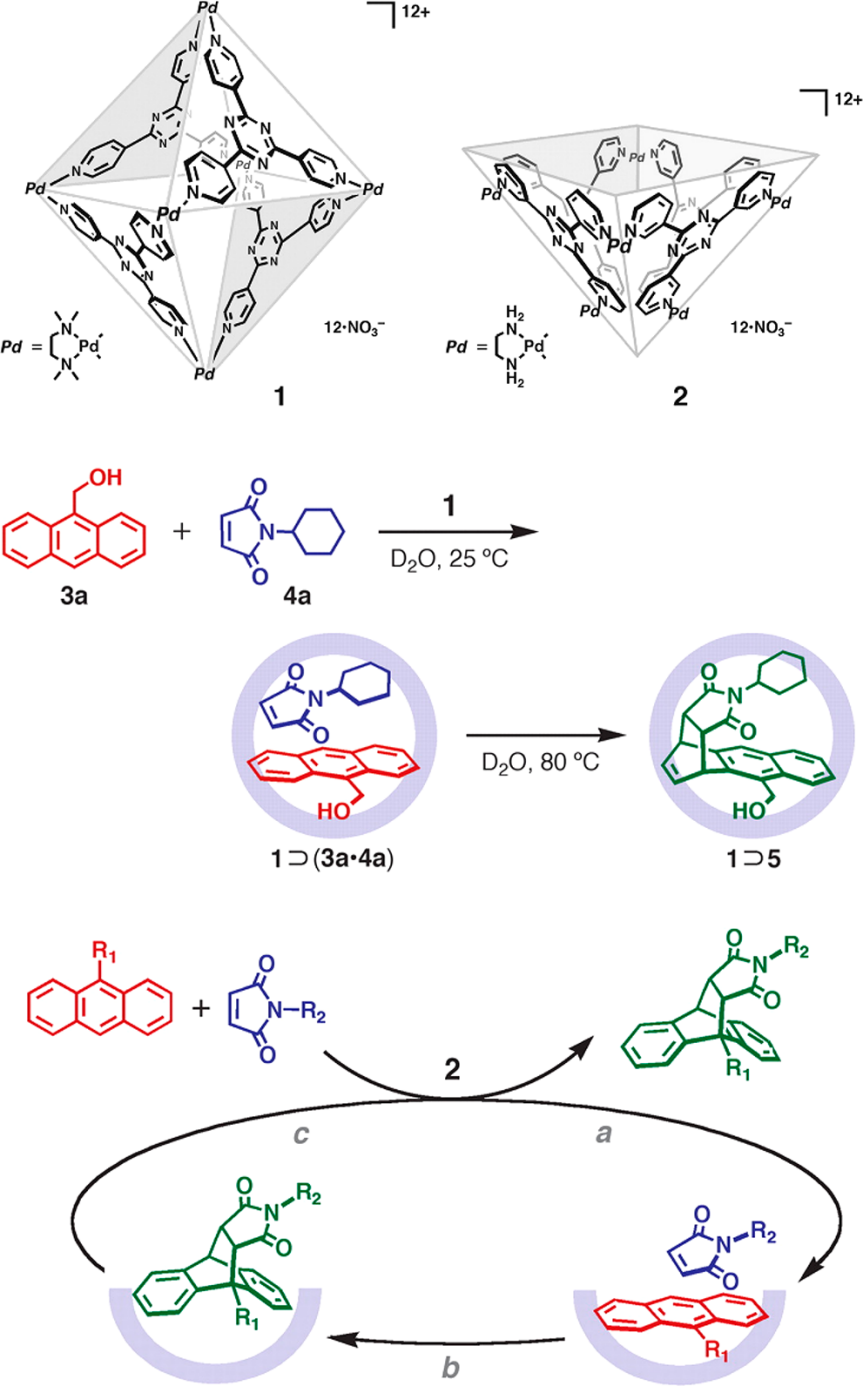
Distinct regioselectivity of Diels–Alder reaction confined within cage-shaped and bowl-shaped self-assembled container molecules. Reproduced with permission from ref (786). Copyright 2006 the American Association for the Advancement of Science.

Self-assembled container molecules are built by building blocks through various interactions, including metal–ligand dative bonds,^782^ covalent bonds (Figure 62),^783^ and hydrogen bonds.^784^ A strength of the noncovalent assembly (metal–ligand and hydrogen bonding) is the ease of preparation, modification, and dynamic guest exchange.^785^ Fujita and co-workers investigated the Diels–Alder reaction of anthracene and *N*-cyclohexylmaleimide in a water-soluble organometallic cage (Figure 63).^786^ Normally, the reaction of anthracene with dienophiles yields the thermodynamically favored 9,10-adduct. However, the steric constraints between the terminal phenyl of the anthracene and the cage led to the *syn*-addition reaction, resulting in a rare 1,4-regioselectivity. Moreover, the reaction turned out to be noncatalytic for bulky maleimide substrates, for example, *N*-propylmaleimide. Rebek and co-workers reported a hydrogen-bonding-based cage as the catalyst for the 1,3-dipolar cycloaddition of phenylacetylene and phenyl azide.^787^ The cage encapsulated the phenylacetylene and phenyl azide, orienting the two groups toward each other. The two reactants showed a 3.7 M concentration for seconds when encapsulated. The initial reaction rate was elevated to ∼6 × 10^–8^ M s^–1^, significantly larger than the original rate. Interestingly, the proximity effect also led to the formation of 1,4-triazole, following the preorganization of substates in the molecular “capsule”. Overall, the 1,3-dipolar cycloaddition in bulk solution yielded a 1:1 mixture of 1,2- and 1,4-cycloadducts.

The Fujita group reported the self-assembly of a chiral M_6_L_4_ cage to achieve an asymmetric [2+2] olefin cross-coupling reaction.^788^ By replacing the ethylenediamine end-caps on the Pd(II) nodes with enantiopure diamines, chirality was introduced into the cages. The deformation of triazine panel was observed in the cages to create the chiral cavities. Even though the chiral diamines are located outward of the cage, the reaction of fluoranthene and *N*-cyclohexylmaleimide results in the formation of the desired [2+2] coupled product with a 40% ee. Regio- and stereoselectivities have been demonstrated in bimolecular radical reactions as well in the same cage.^789^ The reactivity of the as-formed benzylic and semiquinone radicals was mediated by the cage, allowing the selective cross-coupling between the two radical species. The confinement effect was confirmed to cause a conformational change of substrates, leading to a lower activation energy for catalytic reactions. Fujita and co-workers reported the shift from *trans*-planar to *cis*-twisted conformational change of amides when encapsulated into the triazine-based octahedral molecular cages.^790^ The *cis*-twisting caused around five times rate enhancement compared to normal hydrolysis, which is remarkable because of the existence of π-conjugation stabilizing effects of amides.

The Reek group developed a tris(pyridyl) phosphine Rh catalyst assembled within three zinc(II)–porphyrin building units. A capsule cavity was formed in the assembled rhodium catalyst, which showed great catalytic performance improvement (∼100-fold in TOF) in the hydroformylation of 1-octene.^791,792^ Internal alkenes, *trans*-3-octene was also selected to study the regioselectivity.^793^ Both theoretical and experimental results pointed out that while confined in the cage, the rhodium–olefin complex has reduced rotational freedom, leading to regioselectivity. The change of ZnTPP catalyst to ZnPc made the dominant product go from 3-octanal to 2-octanal.^794^ This discovery suggested the strong correlation between the cavity structure and the catalytic selectivity, with ZnPc having an evidently larger cavity than ZnTPP. The Reek group also prepared another Rh catalyst encapsulated by two ZnTPPs installed onto chiral bipyridine phosphoramidite ligands and pushed the application toward the asymmetric hydroformylation of internal alkenes.^795^ By introducing a more rigid bimetallic zinc(II)-Schiff base chelator, bis[Zn(salphen)], a well-defined chiral space was formed between two 3-PyMonoPhos and two bis[Zn(salphen)], with a rhodium centering the molecular assembly.^795^ The developed catalyst led to higher regioselectivity and enantioselectivity, with ratios of 93:7 and 86:14 (*R*:*S*) in the formation of chiral 3-octanal from *cis*-2-octene and *trans*-2-octene, respectively. The enantioselectivity was greatly enhanced to 99:1 (*R*:*S*) with the introduction of another phosphoramidite ligand α, with an *ee* of 73:27 (*S*:*R*) in the hydroformylation of styrene.^796^ The less bulky 1-octene did not show any regioselectivity. Also, the substrate can impact the confinement effect. All previous studies indicate that the confinement effect is a mutually influenced outcome. The Reek and the De Bruin groups studied the zinc–porphyrin iron-capped cubic molecular flask for the catalysis of styrene cyclopropanation.^797,798^ The major deactivation pathways involve the formation of bimetallic species (zinc and iron). They successfully installed cobalt(II)-tetrapyridylporphyrin inside the cubic M_8_L_6_ cage as an “isolated” active site. The subsequent catalyst exhibited a 50% yield in the cyclopropanation of styrene and a 63:37 ratio of trans/cis products was obtained. Remarkably, the result is comparable to the best Co-porphyrin type catalyst developed by Zhang and co-workers (57% yield, 74:26 trans/cis ratio). The cubic molecular flask showed a preference for smaller styrene substrates, demonstrating the confinement effect.

**Figure 64:**
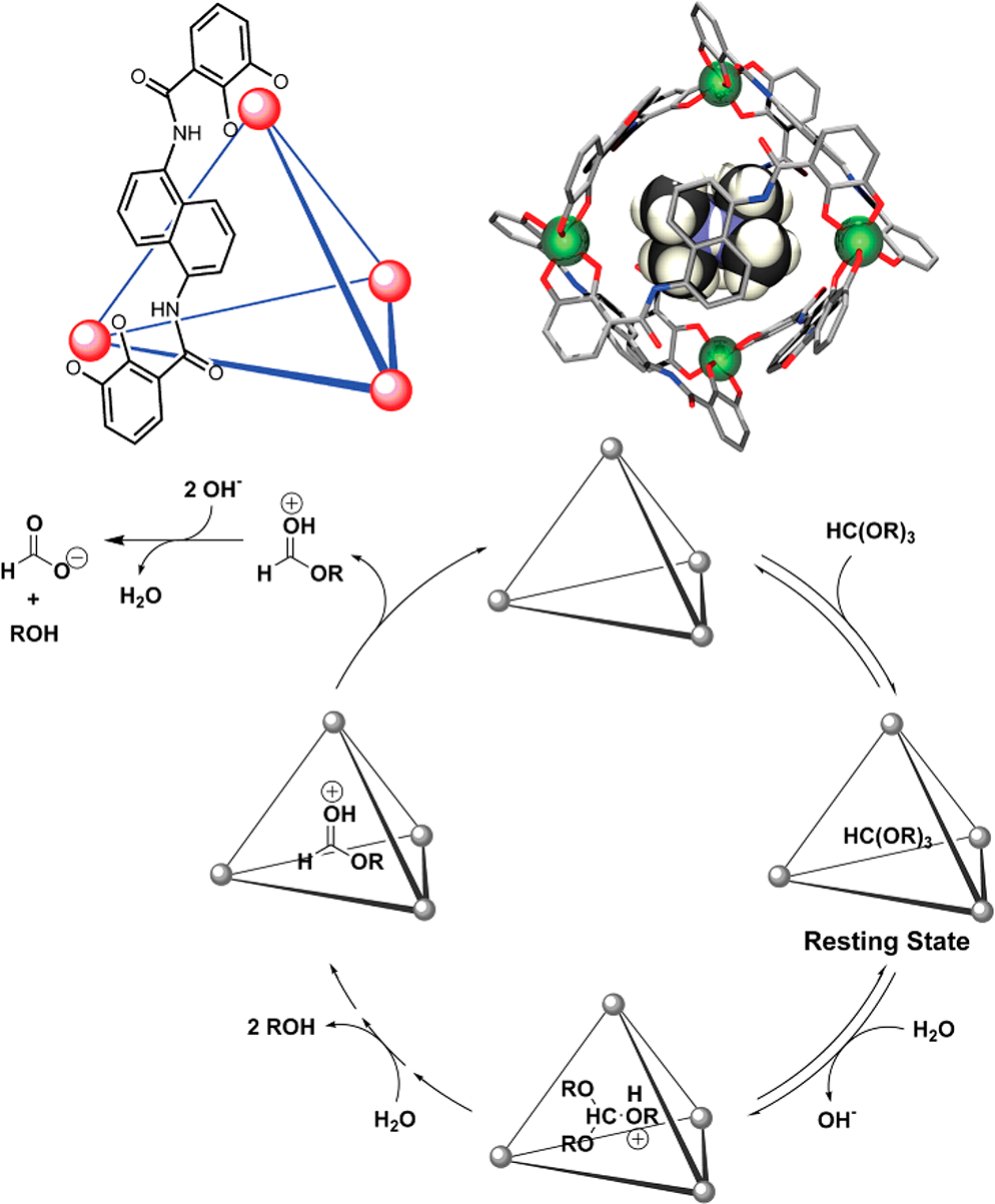
Structural illustration of the [Ga_4_L_6_]^12–^ cage and the catalytic mechanism for orthoformate hydrolysis. Reproduced with permission from ref (799). Copyright 2007 the American Association for the Advancement of Science.

Ionic cages introduce charges onto the cage, granting its ability to attract counterions. For example, the high anionic charge of the cage [Ga_4_L_6_]^12–^ leads to a high local pH, which can protonate the weak basic guest molecules and initiate the reaction (Figure 64).^799^ The Raymond group utilized a gallium-based cage, K_12_Ga_4_L_6_, in the acid-catalyzed Nazarov cyclization with pentadienols as substrates.^22^ The pentadienols turned from linear to U-shaped conformation in the cavity of the [Ga_4_L_6_]^12–^ cage, followed by the combination of a proton to initiate the reaction. The turnover from U-shaped *E*,*E*-pentadienol to cyclopentadiene showed a 2.1 × 10^6^ enhancement, demonstrating the enzymimetic feature of the cage. The cage also favors the subsequent electrocyclization of the dienyl cation intermediate due to ionic interaction, thus promoting the overall reaction. They also reported the cyclization of monoterpene citronellal in the same cage.^800^ The [Ga_4_L_6_]^12–^ cage led to the selective formation of *trans*-2-(1-propen-2-yl)-5,5-dimethylcyclohexan-1-ol, whereas the nonconfined Brønsted acid catalyst produced *trans*-2-(2-hydroxypropan-2-yl)-5,5-dimethylcyclohexan-1-ol. The hydrophobic cavity of [Ga_4_L_6_]^12–^ enables the application of the cage in aqueous solution, preferentially buffer solution. The Me_3_PAu^+^ encapsulated in the cavity was proved to be protected from water. During the gold-catalyzed cycloisomerization of 1,6-enynes, the hydrophobic cavity lowers the chance that the carbenium ion intermediates get captured by water. The same work was also the first example of a terpene cyclization by a water-soluble supramolecular catalyst at physiological pH. Analogues of the cage were also used to study the relationship between the host molecule and the selectivity in catalysis. Variation of the terminal groups does not evidently alter the reaction selectivity, while the change in the size of the cavity by changing the linker fragment from phenyl to naphthalenyl can impact the activity and product selectivity. The Raymond and Bergman groups reported the increased activity in the Au-mediated alkyl–alkyl cross-coupling reaction upon the encapsulation into the [Ga_4_L_6_]^12–^ cage.^801^ The anionic feature of the cage enables it to capture cationic species with compatible sizes. The halide dissociation process takes place before encapsulation, giving rise to a transient cationic gold(III) dialkyl complex, which was subsequently encapsulated into the cage. The nascent gold(III) complex was detected from a Michealis–Menten mechanism, indicating “saturation behavior”. Moreover, the confinement effect accelerates the reductive elimination of the gold(III) complex, thus forming more cross-coupled dialkyl products. Charges can affect the catalytic activity as well. The same group designed an isostructural cage with Si(IV) as nodes.^802^ The variation of [Ga_4_L_6_]^12–^ cage bears 8^–^ charges, noted as [Si_4_L_6_]^8–^. They are compared in catalytic activity through aza-Cope rearrangement reactions, where the two catalysts showed similar reaction rates, *k*_obs_ = 7 × 10^–4^ s^–1^ and 1.0 × 10^–3^ s^–1^ for [Ga_4_L_6_]^12–^ and [Si_4_L_6_]^8–^, respectively. However, in Nazarov rearrangement, there is a dramatic difference in reaction rates, with *k*_obs_= 1.5 × 10^–1^ s^–1^ and 2.2× 10^–4^ s^–1^ for [Ga_4_L_6_]^12–^ and [Si_4_L_6_]^8–^, respectively. Carefully inspecting the reaction mechanism of Nazarov rearrangement, the formation of carbocationic intermediate drew their attention. The anionic host molecule stabilizes the cationic intermediates. The decrease in anionic charges from 12^–^ to 8^–^ brought a large decrease in the rate constant (*k*_obs_).

## 5 Conclusion and Perspectives

### 5.1 Conclusion

Enzymes enable catalyzing thousands of reactions efficiently and selectively. However, many enzymes are prone to permanent denaturation in exposure to high temperature, strong acid and base, losing their delicate superstructures and catalytic ability. MOFs are unique crystalline materials with periodically aligned pores and tailorable functionality. Enzyme immobilization in MOFs has demonstrated its strength overall, in terms of specific enzymatic reactions loaded onto MOFs. It is irrefutable that better catalytic performances have emerged from the MOF–enzyme composites. MOFs provide a solution to address the instability issue of enzymes and also broaden the substrate scope. The diversity of MOFs provides a versatile platform where enzyme immobilization has been extended to numerous cases, and they altogether present guidelines to develop more advanced biocomposites. Besides, enzyme’s active sites can be incorporated into MOFs as building blocks, precluding aggregation and leaching that hamper the long-run stability and efficiency of catalysts. Remarkably, employing active sites as MOFs’ metal nodes and organic ligands creates opportunities to construct biomimicking MOFs with unprecedented topologies and varying pore environments. The densely decorated active sites and high crystallinity of MOFs also ensure clear structural identification. The confinement and templating effects imposed by the nanopores endow the materials with enzyme-like catalytic activity. Note that MOFs stand out among supramolecular catalysts in terms of their three-dimensional architectures, infinite active sites, long lifetime, and superior recyclability. Overall, the structural design and synthesis of bioinspired MOFs and MOF-based composites not only provide coveted catalysts for valuable chemical conversion but also shed light on the structure–activity relationship and mechanisms of enzyme mimic catalysts, which can be viewed as a milestone in building artificial enzymes through self-assembly.

### 5.2 Perspective

Multiple foreseeable challenges still exist in bioinspired MOF catalysis, uncovering novel opportunities to develop MOF–enzyme composites and active-site-embedded MOFs with superior catalytic performance.

(1) The precise control over biocomposites is vital for cascade reactions. Enzymatic spatial allocation in organelles and extracellular spaces are determinants for cascade reactions in organisms. However, it is nearly impossible in MOFs to emulate the exact specialties such as asymmetric environments alongside the membrane and disbalance in concentrations, which could be prerequisites for many reactions. In addition, our reliance on existing enzyme immobilization methods constraints the delicate composite design at the microscopic scale. Therefore, we believe spatial control over the composites ought to be the major research focus in enzyme immobilization. In treating a simple system involving one or two enzymes, the conventional methodologies could be harnessed to achieve high efficacy. In contrast, systems with more than three enzymes may require advanced structural designs to acquire control over the cascade. Herein, precise apportionment of enzymes to emulate organisms might be one possible solution, wherein the integrity and mutual interactions of enzymes are crucial for catalysis. To achieve this aim, it is promising to design and synthesize MOFs with hierarchy in pores and architectures, serving as supporting materials with well-defined enzyme position and structural integrity.

(2) Common species such as organic solvent and oxygen are detrimental to many enzymes and their corresponding active sites, setting up a barrier for practical applications. Although MOF supporters can improve enzymes’ stability toward acid, base, denaturant, and high temperature, studies on immobilizing extremely vulnerable enzymes are still limited. Porous frameworks are prone to concentrating organic solvent and oxygen under working conditions, further deactivating or destructing enzymes. It is intricated to develop MOFs or MOF-based composites that ought to assist enzymes in excluding destructive species. As a compromise strategy, MOFs bearing redox-active sites can play the role as sustainable sacrificial agents to protect enzymes. Furthermore, these studies will also advance the construction of MOFs embedded with air-sensitive active sites, including ones from dehydrogenase and nitrogenase, which are largely untapped materials with significant potentials.

(3) Allostery is a common phenomenon in enzymatic catalysis, wherein the enzyme activity can be regulated through binding effector molecules at a distance site. Inspired by allosteric enzymes, diverse homogeneous systems have been reported as switchable catalysts driven by stimuli like light,^803,804^ redox reactions,^805^ and cation binding.^806^ Current switchable MOF catalysts are mainly based on flexible and redox-active frameworks.^807,808^ We envision that allosteric MOF catalysts with unprecedented dynamic behavior can be synthesized by emulating protein superstructures. One plausible method may be capitalizing on abundant nonbonding interactions to construct MOFs with metastable states. When specific effectors are introduced into MOFs, the frameworks can be transformed into another state with distinct catalytic performance.

(4) As crystalline materials with well-defined structures, biomimetic MOFs can be prepared to trap reactive intermediates during catalysis. Owing to the structural complexity and difficulty in crystallization, the chemical structures and functions of some enzymes’ active sites are not fully understood. Many reactive intermediates studied in solution require trapping the species by freeze quenching in a solvent glass matrix. However, the framework confinement may ease the requirements due to the entropic contribution from confinement within a porous scaffold and be closer to natural systems. Another advantage is that MOF’s active site density is higher than most systems, meaning a higher concentration of reactive intermediates may be present. Typically, low concentrations are needed for soluble complexes to ensure enough distance to prevent intermediate interaction or collapse. In biomimetic MOFs, we may be able to identify and even visualize the intermediate species through a combination of crystallographic and spectroscopic techniques.

(5) Secondary coordination spheres and protein channels are highly associated with the reactivity and selectivity of enzymes, but it is challenging to mimic these superstructures in molecular catalysts.^809^ Herein, the modular structures of MOFs enable the introduction of functionalized ligands to tune hydrogen bonds and other secondary coordination sphere effects.^256,738^ Besides, the pore sizes of MOFs can be readily enlarged or minimized to regulate the mass transfer and substrate selectivity. Note that postsynthetic methodologies will help to diverge one MOF into a large group of MOFs with distinct structures and functions. In particular, improving our ability to tune MOF structures at multiple levels will be necessary for the advancement of catalysis, as it requires optimization of both the active centers and the pore environment of the framework.

Since the advent of machine learning, automated synthesis, and advanced spectroscopic techniques, we envision that research on MOF-based catalysts will be dramatically accelerated. These novel techniques are expected to not only revolutionize the trial-and-error mode in catalyst discovery but also provide principles to design bioinspired MOFs with enzyme-like performance. In addition, enzyme–MOF composites and enzyme mimicking MOFs will also feature great application potentials in diverse areas, including recognition, separation, drug delivery, energy conversion, and optics.

## Abbreviations

AZDC: 4,4′-azobenzene dicarboxylate
BDC: 1,4-benzenedicarboxylate
BPDC: biphenyl-4,4′-dicarboxylate
bpy: 4,4′-bipyridyl
BTC: 1,3,5-benzenetricarboxylate
CAL-B: *Candida antarctica* lipase B
CB: cucurbit[*n*]urils
CD: cyclodextrins
COF: covalent organic framework
CWA: chemical warfare agent
CytC: cytochrome C
DABCO: 1,4-diazabicyclo[2.2.2]octane
DCC: *N*,*N*′-dicyclohexylcarbodiimide
DUT: Dresden University of Technology
EGFP: enhanced green fluorescent protein
FITC: fluorescein isothiocyanate
FTIR: Fourier transform infrared spectroscopy
GDH: glucose dehydrogenase
GOx: glucose oxidase
HOF: hydrogen-bonded organic framework
HKUST: Hong Kong University of Science and Technology
HP: hierarchically porous
HRP: horseradish peroxidase
MAF: metal azolate framework
MIL: Material Institut Lavoisier
MOFs: metal–organic frameworks
NDC: 1,4-naphthalene dicarboxylate
NMR: nuclear magnetic resonance
NP: nanoparticle
NU: Northwestern University
PCN: porous coordination network
PDMS: polydimethysiloxane
PVP: polyvinylpyrrolidone
SBUs: secondary building units
TCPP: *meso*-tetrakis(4-carboxylatephenyl) porphyrin
TED: triethylenediamine
TEPA: tetraethylenepentamine
THF: tetrahydrofuran
TGA: thermogravimetric analysis
TOF: turnover frequency
UiO: Universitetet i Oslo
XO: xanthine oxidase
ZIF: zeolitic imidazolate framework
